# Convalescent Plasma for the Prevention and Treatment of COVID-19: A Systematic Review and Quantitative Analysis

**DOI:** 10.2196/25500

**Published:** 2021-04-07

**Authors:** Henry T Peng, Shawn G Rhind, Andrew Beckett

**Affiliations:** 1 Defence Research and Development Canada Toronto Research Centre Toronto, ON Canada; 2 St. Michael’s Hospital Toronto, ON Canada; 3 Royal Canadian Medical Services Ottawa, ON Canada

**Keywords:** COVID-19, SARS-CoV-2, antibodies, convalescent plasma, immunotherapy, prevention, treatment, review, quantitative, therapeutic, immunology, research, literature, knowledge, recommendation

## Abstract

**Background:**

The COVID-19 pandemic, caused by a novel coronavirus termed SARS-CoV-2, has spread quickly worldwide. Convalescent plasma (CP) obtained from patients following recovery from COVID-19 infection and development of antibodies against the virus is an attractive option for either prophylactic or therapeutic treatment, since antibodies may have direct or indirect antiviral activities and immunotherapy has proven effective in principle and in many clinical reports.

**Objective:**

We seek to characterize the latest advances and evidence in the use of CP for COVID-19 through a systematic review and quantitative analysis, identify knowledge gaps in this setting, and offer recommendations and directives for future research.

**Methods:**

PubMed, Web of Science, and Embase were continuously searched for studies assessing the use of CP for COVID-19, including clinical studies, commentaries, reviews, guidelines or protocols, and in vitro testing of CP antibodies. The screening process and data extraction were performed according to PRISMA (Preferred Reporting Items for Systematic Reviews and Meta-Analyses) guidelines. Quality appraisal of all clinical studies was conducted using a universal tool independent of study designs. A meta-analysis of case-control and randomized controlled trials (RCTs) was conducted using a random-effects model.

**Results:**

Substantial literature has been published covering various aspects of CP therapy for COVID-19. Of the references included in this review, a total of 243 eligible studies including 64 clinical studies, 79 commentary articles, 46 reviews, 19 guidance and protocols, and 35 in vitro testing of CP antibodies matched the criteria. Positive results have been mostly observed so far when using CP for the treatment of COVID-19. There were remarkable heterogeneities in the CP therapy with respect to patient demographics, donor antibody titers, and time and dose of CP administration. The studies assessing the safety of CP treatment reported low incidence of adverse events. Most clinical studies, in particular case reports and case series, had poor quality. Only 1 RCT was of high quality. Randomized and nonrandomized data were found in 2 and 11 studies, respectively, and were included for meta-analysis, suggesting that CP could reduce mortality and increase viral clearance. Despite promising pilot studies, the benefits of CP treatment can only be clearly established through carefully designed RCTs.

**Conclusions:**

There is developing support for CP therapy, particularly for patients who are critically ill or mechanically ventilated and resistant to antivirals and supportive care. These studies provide important lessons that should inform the planning of well-designed RCTs to generate more robust knowledge for the efficacy of CP in patients with COVID-19. Future research is necessary to fill the knowledge gap regarding prevention and treatment for patients with COVID-19 with CP while other therapeutics are being developed.

## Introduction

SARS-CoV-2, the cause of COVID-19, was declared a pandemic in early 2020 by the World Health Organization [1,2]. This is the third coronavirus to emerge in the past two decades, causing multinational outbreaks and carrying substantial morbidity and mortality [3]. COVID-19 is characterized by a spectrum of symptoms, ranging from mild subclinical infection with self-limiting respiratory tract illness (dry cough, fever, fatigue, difficulty breathing) to severe progressive manifestations (acute respiratory distress, hypercoagulation, hyperinflammation, multi-organ dysfunction, death) in high-risk patients with known comorbidities (advanced age, diabetes, obesity, cardiopulmonary disease) [4,5]. Case-fatality rates range from 4% to 50%, with higher mortality observed in the most critically ill [6]. Growing evidence also suggests that some patients with COVID-19, including those with milder symptoms, will have a prolonged course of recovery including fatigue, cognitive impairment, and cardiopulmonary dysfunction [7]. As such, COVID-19 represents an overwhelming universal health crisis [8], and the burden of this disease continues to threaten lives and livelihoods worldwide [9]. As SARS-CoV-2 and its emerging new mutant strains (which may be associated with an increased efficiency of viral replication, transmission, and virulence in humans) continue to spread globally, international research efforts are being accelerated to identify effective preventive and therapeutic approaches to mitigate its impact [10-12].

The magnitude and urgency of this public health emergency has prompted global scientific collaborations to seek rapid solutions via repurposing of previously approved broad-spectrum antivirals (remdesivir, ritonavir, hydroxychloroquine, interferon) [13,14] and therapeutic doses of corticosteroids (dexamethasone, hydrocortisone, methylprednisolone) [15,16] for high-risk patients while fast-tracking development of vaccines and other novel therapeutics [17]. To that end, great advances in understanding the biology of this new coronavirus and the natural history of the disease have been achieved [18,19]. Moreover, the unprecedented development of multiple COVID-19 vaccines capable of eliciting immunological protection, in less than a year from identification of the causative agent, has been a remarkable success and remains the best hope for ending this pandemic [20].

Despite this incredible progress on COVID-19, many challenges remain post vaccine development including ongoing vaccine deployment, large-scale production and distribution of billions of vaccine doses [21], and uncertainty over the effectiveness of current vaccines against more transmissible new variants [22]. These factors, combined with public hesitation around vaccination, have casted doubt on the likelihood of achieving worldwide herd immunity in the near future [23]. Consequently, other therapeutic strategies to impair virus infection or to counteract further disease spread are still needed, at least until more effective drugs are available or vaccines are distributed and administered to everyone [24].

In the absence of definitive treatment against this new human pathogen, clinical management of hospitalized, severely ill patients remains mainly supportive care, including oxygen and mechanical ventilation, and is based largely on preclinical studies or previous experience with severe acute respiratory syndrome–related coronavirus (SARS-CoV) [25]. Thus, an effective evidence-based therapeutic intervention is urgently needed to reduce the morbidity, mortality, and length of in-hospital stay for patients with COVID-19.

Passive immunotherapy with convalescent plasma (CP), hyperimmune γ-globulin, or artificially produced monoclonal antibodies are beneficial for treatment or prophylaxis of several infections, and these approaches are under investigation as potential therapeutic modalities for the management and prevention of COVID-19 [26]. Passive immunotherapy with human convalescent blood products, in particular CP, is a promising strategy for the prevention and treatment of COVID-19 [27-29]. Although further research is needed to determine the utility of immunotherapy with CP or monoclonal antibodies for the treatment of patients who are symptomatic and potentially for use as postexposure prophylaxis, initial findings in limited clinical trials suggest these interventions are safe and can be effective, particularly when administered early in the course of treatment [29]. Experience suggests that CP therapy could be used as an empirical treatment modality to prevent further progression or promote early recovery in patients who are critically ill with COVID-19 [30,31]. CP has been used safely for decades to treat infectious diseases where no specific treatment is available [32,33]. In the late 19th and early 20th century, CP was given to treat a wide range of viral infections, including diphtheria, polio, measles, mumps, and Spanish influenza A (H1N1) [34-36]. Although no randomized trials were conducted, a retrospective meta-analysis of studies on the use of CP during the Spanish influenza flu pandemic showed a significant decrease in mortality in patients who received CP versus those given plasma from unexposed donors [37]. After World War II, plasma became a valuable pharmaceutical component, which used it for diverse products to successfully treat everything from bleeding disorders to immune deficiencies to hypovolemic shock [38]. Since then, CP has been used in outbreaks of Ebola and other coronavirus diseases including SARS-CoV and Middle East respiratory syndrome–related coronavirus (MERS-CoV) infection with varying efficacy [33]. CP was proven to be efficacious in patients with severe 2009 pandemic H1N1 flu, reducing respiratory tract viral load, serum cytokine responses, length of hospital stay, and patient mortality [39]. CP therapy involves transfusing whole or fractionated plasma, collected from patients that have recently recovered from SARS-CoV-2 infection, to confer passive humoral immunity in people who are infected or at risk of infection [29,40]. Furthermore, CP therapy has advantages over other proposed treatment: it requires low technology (and therefore it can be produced where required independent of pharmaceutical companies), it is low cost and has strong biological plausibility, and it has potential for rapid development and deployment (production is easily scalable as long as there are sufficient donors) [41-43]. Accordingly, on March 24, 2020, the Food and Drug Administration (FDA) approved the use of CP therapy as an emergency investigational new drug to treat patients with serious or immediately life-threatening COVID-19 infections [44]. Subsequently, on August 23, 2020, the FDA issued an Emergency Use Authorization (EUA) for CP for treating COVID-19 [45]. According to the FDA regulation, the plasma must be collected from recovered patients who can donate blood, have had no symptoms for 14 days, and have had negative results on COVID-19 tests. Both single-donor and pooled immuno-globulin products currently prioritize collection of convalescent donor plasma with high levels of neutralizing antibodies. Based on the preliminary data from clinical trials and considering the United States National Institute of Health and FDA recommendation, remdesivir and CP are the most promising potential for COVID-19 treatment [46]. CP for treating COVID-19 is accessible via the regulatory pathways (investigational new drug regulatory pathway). Another is expanded access, also called “compassionate use” emergency Investigational New Drug Application (an investigational medical product), to treat patients [47]. It should be noted that, currently, Regeneron’s REGN-COV2 and Lilly’s LY- CoV555, both of which are cocktail therapies comprising receptor binding domain (RBD)–reactive antibodies, have also been granted EUA for COVID-19 by the FDA [48,49].

On the other hand, systematic reviews have been conducted for current medications that have been used for the treatment of COVID-19. A comparative analysis of three treatment modalities for COVID-19, chloroquine and hydroxychloroquine, CP, and remdesivir, found that each modality had both favorable and unfavorable characteristics, but none showed clear evidence of benefit for early outpatient disease or prophylaxis; in particular, chloroquine or hydroxychloroquine is no longer a viable option [50], while CP therapy appeared to show clinical advantages for inpatient use [14]. Moreover, meta-analysis of the safety and efficacy of various interventions including the three treatments and dexamethasone or lopinavir-ritonavir showed that dexamethasone and remdesivir might be beneficial for patients with COVID-19, but the certainty of the evidence was low to very low, so more trials are needed [51].

Studies are currently underway to evaluate use of CP as treatment for patients with severe COVID-19 and to prevent infection (prophylaxis) in certain high-risk patients exposed to COVID-19. Currently, CP is being given to small numbers of hospitalized patients with severe or life-threatening COVID-19 illness [52]. Several case reports suggest treatment is helpful, but larger studies are still needed. Although there is a lot that is unknown, CP may work best for patients earlier in the disease course [53,54]. Therapy using CP may also be beneficial for prophylaxis against SARS-CoV-2 in individuals who are at high risk; there is considerable interest to leverage CP for frontline health care workers, first responders, other caregivers, and vulnerable individuals with underlying medical conditions [55,56]. This strategy has been previously used in SARS-CoV and MERS-CoV outbreaks [57]. Although the evidence for CP therapy remains inconclusive, preliminary trials for CP suggest that there may be some benefits, and there is growing consensus that CP is an important first-line immunotherapy for emerging viral infections when other specific treatments are not available [58]. Currently, several countries and health institutions are collecting CP for either empirical treatment or clinical trials [55,59]. However, research to date is at a high risk of bias, and randomized control trials are desperately needed to determine the efficacy and safety of this therapeutic option.

There are many ongoing trials and reviews, perspectives, commentaries, and guidelines published every day related to all aspects of COVID-19 CP, ranging from donor selection, plasma collection, testing, and storage to clinical use. In this paper, we sought to review all aspects of CP use for COVID-19, from detection of the level and activity of CP antibodies to appraisal of the quality and meta-analysis of original clinical studies of CP therapy, to characterize the knowledge gap and provide recommendations for future directions.

## Methods

This systematic review was conducted in accordance with the PRISMA (Preferred Reporting Items for Systematic Reviews and Meta-Analyses) guidelines [60].

### Search Strategy

We searched relevant databases including PubMed, Web of Science, and Embase from June 19, 2020, for published and unpublished trials with no limitations on starting date, with the terms COVID-19 OR SARS-CoV-2 OR “coronavirus* 2019” AND convalescent plasma/ser*; we continued the search and updated the review during the manuscript preparation until October 22, 2020. Both plasma and serum or sera have been used in the literature. In this review, plasma is representative for both terms.

### Data Abstraction

Titles and abstracts were screened to determine relevance and, if deemed appropriate, the full article was reviewed. Additional publications were selected from the cross-references listed in the original papers and from the cited articles. Disagreements were resolved by consensus or with another review author. The same strategy was used for data extraction and study appraisal as described later.

### Study Eligibility Criteria

Experimental (randomized controlled trials [RCTs], quasi-RCTs, non-RCTs), quasi-experimental (controlled before-after studies, interrupted time series), and observational (cohort, case-control) studies are eligible if they examined CP or serum for prevention, diagnosis, or treatment of COVID-19.

Review articles were excluded unless they were focused on or directly related to CP (eg, passive immunotherapy) for COVID-19. Papers on antibody detection and immunity were also excluded unless specifically related to CP.

### Data Extraction and Study Appraisal

All literature search results were screened independently by two reviewers. The commentaries in support of the use of CP for COVID-19 were considered positive, those suggesting improvements in CP treatment were categorized as neutral, and precautions against CP were determined to be negative. The review type was determined according to a typology of reviews by Grant and Booth [61]. The quality appraisal of included clinical studies was conducted using the Effective Public Health Practice Project (EPHPP) Quality Assessment Tool [62]. Specifically, each clinical study was evaluated for the following components: sample selection, study design, identification and treatment of confounders, blinding of outcome assessors and participants, reliability and validity of data collection methods, and withdrawals and dropouts. The overall rate of each study was determined by assessing the six component ratings. Those with no weak ratings and at least 4 strong ratings were rated strong. Those with less than 4 strong ratings and 1 weak rating were considered moderate. Those with 2 or more weak ratings were rated weak.

### Analyses

Studies were analyzed separately according to their design (case report, case series, observational, or randomized trials). Clinical and methodological heterogeneities across the studies were assessed by examining the details of the patients, the baseline data, the interventions, and the outcomes to determine whether the studies were sufficiently similar.

For disease severity, severe COVID-19 is a clinical situation in which the patient has dyspnea, tachypnea (respiratory rate≥30 breaths/minute), blood oxygen saturation≤93% on room air, partial pressure of arterial oxygen to fraction of inspired oxygen ratio <300 PaO_2_/FiO_2_<300, or lung infiltrates >50% within 24-48 hours on chest x-ray [63]. Life-threatening disease is defined as respiratory failure, septic shock, or multiple organ dysfunction or failure [63].

Case and randomized controlled studies were combined in meta-analyses using Review Manager (Version 5.4, The Cochrane Collaboration). Data were pooled using an inverse variance method and analyzed using a random-effects model, as this approach accommodates clinical and statistical variations. Odds ratios (ORs) and 95% CIs were used as statistical measures for mortality, clinical improvement, and viral clearance as a dichotomous outcome. Mean and SD were the statistical measure used to describe length of hospital stay. In studies that reported data in medians and IQRs, mean and SD were estimated using the sample size in each study arm, the medians, and the first and third IQRs as demonstrated in the method published by Wan et al [64]. Heterogeneity was determined using the I^2^ statistic and the chi-square test. High values of both tests (I^2^>40%, a significant chi-square value with *P*<.05) demonstrate high levels of inconsistency and heterogeneity.

## Results

### Overall Findings

**Figure 1:**
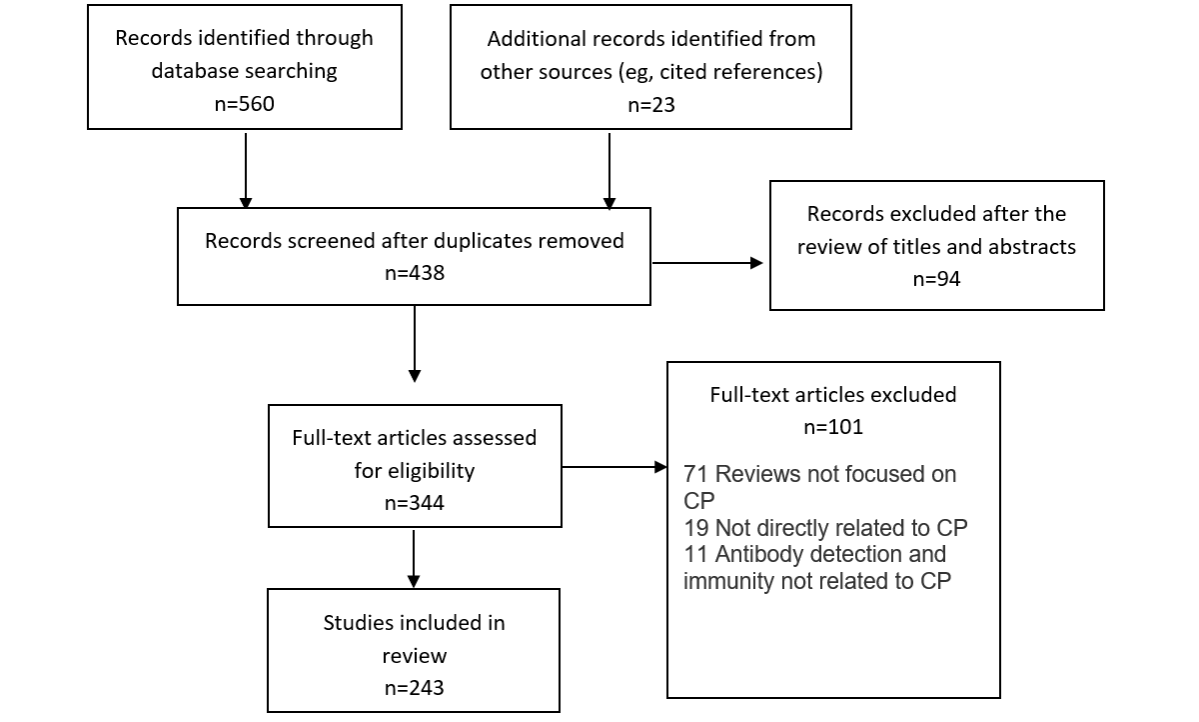
PRISMA (Preferred Reporting Items for Systematic Reviews and Meta-Analyses) flow diagram. The literature search was conducted on June 19 and updated on October 22, 2020. The screening, full-text review, and extraction were managed online using Covidence. CP: convalscent plasma.

As illustrated in Figure 1, we reviewed 438 titles and abstracts and identified 243 manuscripts relevant to five areas of focus or types: (1) original clinical studies; (2) commentary in the form of letter to the editor, correspondence or editorial, brief communication, opinions, perspectives, and viewpoints; (3) review of the use of CP; (4) protocol or guidance for clinical trials or production of CP; and (5) in vitro testing of CP.

A total of 243 articles were included in this review. As summarized in Table 1, they can be stratified as follows: 64 clinical studies (20 case reports, 31 case series, 11 case-control studies, and 2 RCTs), 79 commentary articles, 46 reviews, 19 guidance and protocols, and 35 in vitro testing of CP antibodies.

**Table 1:** Summary of literature.

Article type^a^ and group		Articles, n	Summary	References
**Clinical studies**		64		
	Case reports		A single severe or critically ill COVID-19 patient of different ages (6-100 years), either previously healthy or with comorbidities (cancers, organ transplantation, immunodeficiency, hypertension, diabetes, cerebral hemorrhage, cardiopulmary disease, or pregnancy), was successfully treated with one or two doses of CP^b^ (150-250 mL per dose; anti–SARS-CoV-2 IgG titer 1:13.3-1:700) in combination with antiviral or anti-inflammatory drugs (favipiravir and hydroxychloroquine, enoxaparin, methylprednisolone, remdesivir, lopinavir or ritonavir, prednisone), antibiotic therapy (azithromycin, ceftriaxone moxifloxacin, piperacillin, tienam), antifungal medication (fluconazole), or prophylactic low-molecular-weight heparin	Al Helali et al 2020 [65] Anderson et al 2020 [66] Bao et al 2020 [67] Cinar et al 2020 [68] Clark et al 2020 [69] Figlerowicz et al 2020 [70] Grisolia et al 2020 [71] Hahn et al 2020 [72] Hartman 2020 [73] Im et al 2020 [74] Jafari et al 2020 [75] Jiang et al 2020 [76] Karataş et al 2020 [77] Khan et al 2020 [78] Kong et al 2020 [79] Mira et al 2020 [80] Rodriguez et al 2020 [81] Soleimani and Soleimani 2020 [82] Xu et al 2020 [83] Zhang et al 2020 [84]
	Case series		31 clinical studies involving two or more COVID-19 patients of different ages (14-91 years) and disease severity (eg, hospitalized, moderate, severe, or life-threatening), either previously healthy or with comorbidities (cancer, hypertension, immunosuppression, organ transplantation) that were treated with various doses of CP (200 mL to 3 × 200 mL) in addition to supportive care, antiviral therapy, antibiotics, steroids, or anticoagulation treatment.	Ahn et al 2020 [85] Abdullah et al 2020 [86] Bradfute et al 2020 [87] Diorio et al 2020 [88] Enzmann et al 2020 [89] Erkurt et al 2020 [90] Fung et al 2020 [56] Gemici et al 2020 [91] Hartman et al 2020 [63] Ibrahim et al 2020 [92] Bobek et al 2020 [93] Jin et al 2020 [94] Joyner et al 2020 [95-97] Liu et al 2020 [98] Maor et al 2020 [99] Naeem et al 2020 [100] Olivares-Gazca et al 2020 [101] Pal et al 2020 [102] Rahman et al 2020 [103] Salazar et al 2020 [104] Shen et al 2020 [105] Tremblay et al 2020 [106] Wei et al 2020 [107] Wang et al 2020 [108] Wu et al 2020 [109] Xi et al 2020 [110] Ye et al 2020 [111] Zhang et al 2020 [112] Zeng et al 2020 [113]
	Observational (cohort, case-control studies)		11 cohort, case-control studies of a CP treatment group (6-316 patients) and a matched control (12-1430 patients) of severe or life-threatening COVID-19 patients to compare clinical and laboratory outcomes including all-cause mortality, total hospitalization days, and patients’ need for intubation between the two groups.	Abolghasemi et al 2020 [114] Duan et al 2020 [115] Hegerova et al 2020 [116] Liu et al 2020 [117] Perotti et al 2020 [118] Rasheed et al 2020 [119] Roger et al 2020 [120] Salazar et al 2020 [121] Xia et al 2020 [122] Xiao et al 2020 [123] Zeng et al 2020 [124]
	RCT^c^		Two RCTs of 86 hospitalized and 103 severe or life-threatening COVID-19 patients randomized at 1:1 ratio for standard of care therapy with and without CP. The primary outcome was mortality and time to clinical improvement.	Gharbharan et al 2020 [125] Li et al 2020 [126]
**Commentary (correspondence, editorial, letter to the editor, opinions, perspectives, viewpoints)**		79		
	Positive		These are commentaries that supported clinical use and evaluation of CP for COVID-19 treatment based on the unique immunomodulatory properties of CP and historical and current data for its safety and efficacy against coronaviruses including SARS-CoV-2 but suggested limitations, future clinical investigations, and a variety of aspects to be considered for the optimal use of CP for COVID-19 including CP donor selection, CP collection and testing, manufacturing turnaround time, cost and the logistics of storage, distribution, treatment population, and administration timing and dosing.	Alghamdi and Abdel-Moneim 2020 [127] Alzoughool and Alanagreh 2020 [128] Borlongan and Sanberg 2020 [129] Cantore and Valente 2020 [130] Casadevall and Pirofski 2020 [34] Casadevall et al 2020 [131] Chen et al 2020 [28] Cheraghali et al 2020 [132] Gazzaruso et al 2020 [133] Farhat et al 2020 [134] Focosi et al 2020 [135] Franchini 2020 [136] Franchini et al 2020 [137-139] Islam et al 2020 [140] Kesici et al 2020 [141] Knudson and Jackson 2020 [142] Kumar et al 2020 [143] McAllister et al 2020 [144] Montelongo-Jauregui et al 2020 [36] Morabito and Gangadharan 2020 [29] Nnaji et al 2020 [145] Pau et al 2020 [146] Perez-Cameo and Marin-Lahoz 2020 [41] Rabelo-da-Ponte et al 2020 [147] Roback and Guarner 2020 [148] Roberts et al 2020 [149] Rubin 2020 [47] Sabando Velez et al 2020 [150] Sahu et al 2020 [151] Sheikh and Baig 2020 [152] Sheridan 2020 [43] Syal 2020 [153] Teixeira da Silva 2020 [154] The Lancet Haematology 2020 [155] Tonn et al 2020 [156] Wong and Lee 2020 [157] Yoo 2020 [158] Zhao and He 2020 [159] Zhu et al 2020 [160]
	Neutral		This group of articles highlighted both pros and cons of CP therapy and alternative therapeutic options (eg, equine polyclonal antibodies) for COVID-19, and raised questions regarding neutralizing antibodies, donor selection, collection, testing and qualification of CP, time frame for transfusing CP to recipients, transfusion volume, quality of evidence for the safety, efficacy, and ethics of clinical trials of CP therapy.	Tamburello and Marando 2020 [161] Begum and Ray 2020 [162] Bloch 2020 [163] Brown 2020 [164] Casadevall et al 2020 [165,166] Cunningham et al 2020 [167] Dhanasekaran et al 2020 [168] Dzik 2020 [169] Estcourt and Roberts 2020 [170] Farrugia 2020 [171] Fleming and Raabe 2020 [172] Focosi 2020 [173] Garraud 2020 [174] Gniadek and Donnersberger 2020 [175] Han and Zhou 2020 [176] Langhi et al 2020 [177] Lanza and Seghatchian 2020 [178] Mahase 2020 [179] Mahase 2020 [180] Malani et al 2020 [58] Adiwinata Pawitan 2020 [181] Prajapati 2020 [182] Saverino 2020 [183] Stevens et al 2020 [184] Tedder and Semple 2020 [185] Van den Berg et al 2020 [186] Verkerke and Maier 2020 [187] Xi 2020 [188] Zeng et al 2020 [189] Zylberman et al 2020 [190]
	Negative		This group of commentaries suggested that the risks associated with CP use (eg, adverse effects and blood-borne pathogen transmission) outweighed its benefits or other therapeutics for COVID-19.	Caccamo et al 2020 [191] Ferreira and Mostajo-Radji 2020 [192] Joob and Wiwanitkit 2020 [193] Sanfilippo et al 2020 [194,195] Wiwanitkit 2020 [196]
**Review**		46	46 different types of reviews (a total of 10 review types with unique features in terms of prescribed and explicit methodologies) on CP for treatment of virus infectious diseases (eg, SARS^d^, MERS^e^, EBOV^f^, and H1N1) and COVID-19 with safety and efficacy as main outcomes and recommendations. Some reviews also covered other aspects related to CP use, such as SARS-CoV-2 immunology, mechanism of action, CP donor selection, CP collection, pooling technologies, pathogen inactivation systems, banking of CP, timing and dose of CP treatment, patient selection, risk-benefit analysis, and list of ongoing registered clinical trials.	
	Rapid review			Barone and DeSimone 2020 [197] Majbour and El-Agnaf 2020 [198]
	State-of-the-art review			Brown and McCullough 2020 [199] Focosi et al 2020 [27]
	Scoping review			Cao and Shi 2020 [200] Zheng et al 2020 [201]
	Review of the evidence			de Alwis et al 2020 [202] Fischer et al 2020 [203] Mucha and Quraishy 2020 [204]
	Systematic review and meta-analysis			Chai et al 2020 [205] Devasenapathy et al 2020 [206] Piechotta et al 2020 [207] Sarkar et al 2020 [208] Sun et al 2020 [209]
	Overview			Abdollahi et al 2020 [210] Annamaria et al 2020 [211] Anudeep et al 2020 [212] Bloch et al 2020 [55] Venkat Kumar et al 2020 [213] Gasparyan et al 2020 [214] Iftikhar et al 2020 [215] Li et al 2020 [216] Lindholm et al 2020 [217] Murphy et al 2020 [218] Sayinalp et al 2020 [219] Subbarao et al 2020 [220]
	Mixed studies review			Pawar et al 2020 [221]
	Systematic review			Bakhtawar et al 2020 [222] Chen and Xia 2020 [223] Rajendran et al 2020 [224] Valk et al 2020 [225] Wooding and Bach 2020 [57]
	Critical review			Focosi and Farrugia 2020 [226] Nagoba et al 2020 [227] Psaltopoulou et al 2020 [228] Tiberghien et al 2020 [59]
	Literature review			Choi 2020 [52] Khulood et al 2020 [229] Chua Vi Long et al 2020 [230] Ouyang et al 2020 [231] Piyush et al 2020 [232] Rojas et al 2020 [233] Selvi 2020 [234] Sharun et al 2020 [235] Sullivan and Roback 2020 [236] Yigenoglu et al 2020 [237]
**Protocol/guidance**		19	These are protocols for clinical trials to evaluate the safety and efficacy of CP in treating COVID-19 patients, guidelines or programs for CP donor selection, CP preparation, laboratory examination, storage, distribution, dose, frequency and timing of CP administration, targeted patients, parameters to assess response to the treatment and long‐term outcome, adverse events, and CP application in resource-limited countries and in pediatrics and neonates.	
	Preparation/production of CP			Accorsi et al 2020 [238]
	Protocol for a nonrandomized trial			Albalawi et al 2020 [239]
	Clinical study and application of CP			Al-Riyami et al 2020 [240]
	Conceptual framework			Albahri et al 2020 [241]
	Expert opinion, survey of group members, and review of available evidence			Bloch et al 2020 [242]
	COVID-19 CP program			Blackall et al 2020 [243] Budhai et al 2020 [244]
	Study protocol for RCTs			Chowdhury et al 2020 [245] Eckhardt et al 2020 [246] Janssen et al 2020 [247]
	Perspective document of the Working Party on Global Blood Safety of the International Society of Blood Transfusion			Epstein and Burnouf 2020 [248]
	Commentary			Epstein et al 2020 [249]
	Guidance for treating early to moderate COVID-19 patients with CP			Hassan et al 2020 [250]
	Initiative for provision of CP			Ipe et al 2020 [251]
	A pilot program of CP collection			Li et al 2020 [252]
	Strategy and experience			Pei et al 2020 [253]
	One arm proof-of-concept clinical trial protocol			Perotti et al 2020 [254]
	An apheresis research project proposal			Seghatchian and Lanza 2020 [255]
	Authority guide by Turkish Ministry of Health			Yilmaz et al 2020 [256]
**In vitro testing of convalescent plasma**		35		
	ELISA^g^ with virus antigens (eg, spike and NP^h^ protein sequences) or recombinant ACE-2^i^ as substrates		An ELISA could be a high-throughput competitive assay to detect different antibody types against SARS-CoV-2 in serum and plasma from convalescent patients; to estimate the neutralizing capacity of antispike protein antibodies to block interaction with the human ACE-2 required for viral entry; and to identify candidate sera for therapeutic use. A combination of antigenic targets (NP, spike protein, S-RBD^j^) may improve the accuracy of IgG detection in CP donors.	Amanat et al 2020 [257] Byrnes et al 2020 [258] Gattinger et al 2020 [259] Zhang et al 2020 [84] DomBourian et al 2020 [260]
	Pseudovirus capture assay, VN^k^ assay using SARS-CoV-2 strains and Vero-E6 cells		In vitro evaluation of CP potency for COVID-19 treatment could be measured by its binding capacity to the SARS-CoV-2 spike protein and neutralizing activity against pseudotyped and chimeric viruses and authentic SARS-CoV-2, which is useful to identify donors with high titers for CP for COVID-19 therapy. There were individual differences in the antibody level (neutralizing antibody titers <1:16 to >1:1024) and its changes over 12-60 days since onset of symptoms among representative convalescent patients.	Ding et al 2020 [261] Ianevski et al 2020 [262] Schmidt et al 2020 [263] Wang et al 2020 [264] Muruato et al 2020 [265]
	Immunoassays for anti–SARS-CoV-2 IgM, IgG, and IgA based on SARS-CoV-2 SP		CP collected from adults who met all criteria for donating blood had confirmed COVID-19 by positive SARS-CoV-2 PCR^m^ test and completed resolution of symptoms at least 14 days prior to donation showed a wide range of antibody levels. Total anti–SARS-CoV-2 NP antibody strength correlated with time from symptom resolution to sample collection and symptom duration. There was a decline in the IgG level over a short duration of 10 days. RBD^n^-specific serum IgG, IgM, and IgA COVID-19 convalescent patients continued to decline from 28 to 99 days after hospital discharge. Anti–SARS-CoV-2 spike protein IgG antibody strength correlated with age and hospitalization for COVID-19.	Ragnesola et al 2020 [266] Yang et al 2020 [267] de Assis et al 2020 [268] Dulipsingh et al 2020 [269] Ikegami et al 2020 [270] Ma et al 2020 [271]
	PCR-based tests		SARS-CoV-2 neutralizing antibodies were detectable as early as 10 days after onset of symptoms and continue to rise, plateauing after 18 days and were not altered by amotosalen and UV-A radiation to inactivate potentially contaminating infectious pathogens in CP. Detectable viral RNA in older COVID-19 patients screened for CP donation even 12-24 days after symptom resolution.	Danh et al 2020 [272] Hartman et al 2020 [273]
	VN assays based on pseudotyped and live SARS-CoV-2 virus, and anti–SARS-CoV-2 IgM, IgG, and IgA ELISA based on virus antigens and ACE-2		The levels of anti–SARS-CoV-2 IgM, IgG, and IgA and the neutralization capacity of CP showed a wide range and changed over time after the onset of COVID-19 symptoms and declined within the first 3 months following diagnosis, suggesting an optimal time period for CP collection. Both could be associated with donor’s age, sex, weight, COVID-19 severity, days between disease onset and plasma collection. There were various degrees of positive correlations (coefficients 0.21-0.87) between the VN and ELISA results. Some commercial ELISA can perform effectively as surrogate assays for predicting neutralizing antibody titres.	Abe et al 2020 [274] Beaudoin-Bussières et al 2020 [275] Benner et al 2020 [276] Boonyaratanakornkit et al 2020 [277] Gniadek et al 2020 [278] Patel et al 2020 [279] Harvala et al 2020 [280] Wendel et al 2020 [281] Zeng et al 2020 [282] Dogan et al 2020 [283] Jungbauer et al 2020 [284] Li et al 2020 [285] Ni et al 2020 [286] Robbiani et al 2020 [287] Salazar et al 2020 [288] Weidner et al 2020 [289]
	Biophysical antibody profiling		CP antibodies can elicit Fc-dependent functions beyond viral neutralization such as complement activation, phagocytosis, and antibody-dependent cellular cytotoxicity against SARS-CoV-2.	Natarajan et al 2020 [290]

^a^The articles were classified into five types: 64 clinical studies (20 case reports, 31 case series, 11 case-controlled and two RCTs), 79 commentary articles, 46 reviews, 19 guidance and protocols, and 35 in vitro testing of CP antibodies. The details are shown in Table S1 in Multimedia Appendix 1.

^b^CP: convalescent plasma.

^c^RCT: randomized controlled trial.

^d^SARS: severe acute respiratory syndrome.

^e^MERS: Middle East respiratory syndrome.

^f^EBOV: Ebola virus.

^g^ELISA: enzyme-linked immunosorbent assay.

^h^NP: nucleocapsid protein.

^i^ACE2: angiotensin converting enzyme 2.

^j^S-RBD: spike protein receptor-binding domain.

^k^VN: virus neutralization.

^l^SP: spike protein.

^m^PCR: polymerase chain reaction.

^n^RBD: receptor-binding domain.

All clinical studies are therapeutic use of CP focusing on safety and efficacy, and they are further reviewed in the following section. The commentaries cover various aspects of CP, ranging from critiques of clinical studies [131,137,148,163,176,189] and literature review [145,221] to the stability of antibodies in CP [156,291], relevant news [180], and a response letter [164], while a majority focused on the safety and efficacy of CP. Most commentaries were in favor of CP therapy for COVID-19, recognizing the need for more high-quality evidence from large and well-designed clinical trials to show its efficacy, and other issues (eg, CP collection) still need to be addressed. Some commentaries proposed alternative or complementary CP-based approaches to COVID-19 that possess fewer risks [178,182] but may not be immediately available for clinical use. Only a few commentaries put more emphases on the potential risks over benefits of CP therapy [191-194,196].

In a particular correspondence, a metadata analysis of the efficacy of CP treatment based on 9 clinical studies (mostly case series) suggested that CP reduced viral loads (risk ratio 0.13, 95% CI 0.09-0.18; *P*<.001; n=75) and C-reactive protein levels (ratio of mean [ROM] 0.11, 95% CI 0.01-0.86; *P*<.05; n=42), and improved the clinical status of patients with COVID-19 (ROM 0.53, 95% CI 0.36-0.79; *P*<.01; n=149) when compared to baseline (date of CP transfusion) [147]. In addition, the effects of CP on C-reactive protein levels and clinical improvement were not associated with the patient’s age and the use of antivirals, antibiotics, and hydroxychloroquine. Several commentary papers and reviews advocated for the rationale of developing fast access to CP collection and treatment of patients with COVID-19 [34,47,59,148,199,229]. Among the reviews, most were descriptive overviews of existing literature and recommendations for clinical use and trial without any search strategies. Few were conducted following the PRISMA guidelines [222,224,225]. It is noteworthy that 1 systematic review and meta-analysis was on the safety and efficacy of CP therapy for other severe respiratory viral infections to provide indirect evidence for CP therapy for COVID-19 [206], and another 2 systematic reviews and meta-analyses were on completed and ongoing clinical studies on the safety and efficacy of CP or hyperimmune immunoglobulin transfusion in the treatment of COVID-19 [207,208]. One review and meta-analysis included 20 studies (1 RCT, 3 controlled nonrandomized studies of interventions, 16 noncontrolled nonrandomized studies of interventions) with 5443 participants [207]. The meta-analysis of 4 controlled studies (1 RCT and 3 controlled nonrandomized studies of interventions) with 339 patients could not support any effects of CP treatment on all-cause mortality at hospital discharge, time to death, or improvement of clinical symptoms at 7 days. The review also investigated the safety of CP based on 14 studies (5201 participants, with 5000 participants from 1 noncontrolled nonrandomized studies of intervention) and found very low-certainty evidence for safety. The review was recently updated, which included 19 studies with 36,081 patients treated by CP, and made the same conclusion [205]. The other review included 7 studies, including 2 RCTs and 5 cohort studies, with a total of 5444 patients [208]. The meta-analysis indicated that CP therapy reduced mortality and increased viral clearance and clinical improvement. It confirmed the safety of CP transfusion with very low incidence of serious adverse events. However, the risk of bias and quality assessment in both reviews indicated that the evidence for the efficacy and safety of CP therapy was of low quality, suggesting the need for a large well-designed RCT. In addition, a survey has been conducted for current registered clinical trials of CP therapy for COVID-19, including a description of their characteristics such as study design, patient populations, outcomes, eligibility criteria for CP donors, CP collection, antibody titer, and CP dose [218].

Protocols, programs, and standards have been developed to select donors and collect, process, characterize, store, distribute, and apply CP to patients in need [238,240,242,250], and to conduct clinical trials [239,246,247,254]. Regional and national programs for COVID-19 CP have been established [243,244] as well as a multi-criteria decision-making frame for both CP donor and receipt selection [241].

Some key findings and implications from the in vitro testing studies of CP antibodies should be considered: a variety of methods have been developed to measure CP antibody titers including gold standard neutralization assay using living SARS-Cov-2 [261,262]; enzyme-linked immunosorbent assay (ELISA) using the antigens derived from the virus, mostly in a microplate platform [257,258] and a few in lateral flow [266], microsphere [267], and microarray platforms [268]; and other methods (eg, polymerase chain reaction [PCR] tests) [272,273]. A number of studies showed a wide range of levels and neutralizing activities of anti–SARS-CoV-2 [264,267,289]. The neutralizing antibody levels declined within the first 3 months following diagnosis, suggesting a short optimum time window for the collection of CP with high neutralizing antibody titers [280]. A significant decrease was also observed in the antibody binding to the spike protein of SARS-CoV-2 and neutralizing capacity of plasma from convalescent donors at 6 and 10 weeks after symptoms onset [261]. The short duration of neutralizing antibody titers within months may have important implications for immunity and ongoing efforts to deploy CP for prevention and therapy of COVID-19 [165]. There is a significant correlation to various extents between ELISA-measured immunoglobulin (IgG) titer and neutralizing antibody titer [87,257,274,276,278-280,283-285,288,289]. However, the ELISA-determined anti–SARS-CoV-2 IgG did not always inhibit the virus receptor binding [259]. Antibody binding to SARS-CoV-2 spike glycoprotein as measured by pseudovirus capture assay did not always translate into neutralization [261].

Highly sensitive and specific platforms for the detection of anti–SARS-CoV-2 antibodies are becoming increasingly important for evaluating potential CP donors and identifying individuals with seroconversion [292]. Various platforms demonstrate significant correlations with a SARS-CoV-2 plaque reduction neutralization assay, suggesting their use for screening of individuals who have recovered from SARS-CoV-2 infections. Notably, a novel multiplexed solid-phase chemiluminescence immunoassay has been developed and commercially available from Meso Scale Discovery for simultaneous detection of IgG binding to four SARS-CoV-2 antigens (trimeric spike, spike RBD, spike N terminal domain, and nucleocapsid antigen) and the quantification of antibody-induced angiotensin-converting enzyme 2 (ACE-2) and ACE-2–binding inhibition (pseudo-neutralization assay) [293].

In addition to neutralization and immune assays, biophysical and functional evaluation of CP showed that it may have diverse antiviral effects against SARS-CoV-2 beyond neutralization, namely, antibody-dependent cellular cytotoxicity, phagocytosis, and complement activation [290]. Moreover, CP could act not only on the viral infection but also on the antithrombin deficiency to reduce thromboembolic events [133].

### Findings of Clinical Studies

As summarized in Table 2, there are considerable heterogeneities among the clinical studies in terms of the populations, the amount of CP received, and a variety of comparators. The CP therapy studies differed in the following aspects: patient demographics (eg, age, gender, and comorbidities), donors’ selection (ie, age, gender, diagnosis of SARS-CoV-2 infection and of recovery, and anti–SARS-CoV-2 antibody titer required for plasma donation), plasma collection and biologic qualification (number, volume and frequency of donations, infectious disease markers, and pathogen inactivation), and treatment and disease characteristics (dose and timing of administration, stage of the disease at which to start CP treatment).

**Table 2:** Summary of original clinical studies of CP therapy for COVID-19. The studies were stratified according to the study design.

Study design and studies		Population	Details of CP^a^	Interventions and comparisons	Outcomes/main findings	Adverse events related to CP therapy
**Case report**						
	Al Helali et al 2020 [65]	A previously healthy male 55 years of age with severe COVID-19	Not reported	About 300 mL CP was transfused over 1 h in addition to other therapeutics: favipiravir, hydroxychloroquine, enoxaparin, paracetamol, diphenhydramine	A significant radiological and clinical improvement in a few days after CP transfusion and negative PCR^b^ test for COVID-19 in <48 h and discharged 12 days post transfusion	No significant adverse effects
	Anderson et al 2020 [66]	A pregnant critically ill female 35 years of age with COVID-19 and past medical history for type 2 diabetes mellitus, asthma, and class III obesity	Not reported	One unit of CP on the day of admission at ICU^c^ and supportive care and therapeutic agents	Discharged on day 14 with no further issues afterward and continuing antenatal care with both primary obstetric office and maternal fetal medicine specialists	Not reported
	Bao et al 2020 [67]	A critically ill man 38 years of age infected by SARS-CoV-2 and had cerebral hemorrhage	Not reported	150-200 ml CP of type A Rh positive was given twice 9 days after hospital admission in addition to antiviral and antibacterial treatment	Both SARS-CoV-2 nucleic acid tests were negative (24 h interval) 2 days after the transfusion, and the patient’s symptoms gradually stabilized	Not reported
	Cinar et al 2020 [68]	A male patient 55 years of age with severe COVID-19 and active myeloid malignancy, disseminated tuberculosis, and kidney failure	Collected using Trima Accel Automated Blood Collection System from a donor who had previously recovered from COVID-19 and met universal donation criteria, anti–SARS-CoV-2 IgG titer 6.6	200 mL of CP on fifth day of symptom onset and another 200 mL of CP at ICU, in combination with antiviral and anticytokine drugs	SARS-CoV-2 was negative, discharged from the hospital with full recovery	No adverse reaction or complication
	Clark et al 2020 [69]	Immunocompromised woman 76 years of age with persisting COVID‐19 following therapeutic lymphocyte depletion	Not reported	CP transfused at day 50 after symptom onset over 2 days (200 mL/day) in addition to treatment with lopinavir/ritonavir and prednisone	Rapid improvement in health condition, allowing definitively withdrawing oxygen, apyrexia ensued, and negative SARS-CoV-2 test; discharged on day 69	No adverse events
	Figlerowicz et al 2020 [70]	A girl 6 years of age with severe COVID-19	CP inactivated using methylene blue with anti–SARS-CoV-2 IgG at a titer of 1:700	CP transfused once in a 200-mL dose at 5 weeks from the beginning of the disease and treatment with antiviral drugs and immune modulators, antibiotics, and antifungal drugs	SARS-CoV-2 was negative for the next 3 weeks after CP therapy. The hematologic parameters did not improve after SARS-CoV-2 elimination.	No adverse events
	Grisolia et al 2020 [71]	A woman 29 years of age at 24 2/7 weeks of gestation	Not reported	The patient was transfused with 300 mL of CP on day 7 from onset of symptoms and another 300 mL of CP on day 12, and treated with antibiotics, low-molecular-weight heparin, hydroxychloroquine, and methylprednisolone	The patient’s clinical condition rapidly improved as shown by normalization of laboratory tests, body temperature, O_2_ saturation, and vital signs within 3 days of the second CP transfusion, discharged 13 days after admission	No adverse effects
	Hahn et al 2020 [72]	A previously healthy man in his 70s with severe COVID-19 admitted to ICU	Obtained from two blood donors with one being diagnosed with high-level anti–SARS-CoV-2 IgG antibody	A total of 900 ml of CP was transfused at a slow infusion rate on day 31 after admission and treatment with a respirator, muscle relaxants, and antibiotics	The patient became afebrile and was tested negative for SARS-CoV-2 the following day after CP therapy, gradually improved and was weaned from the ventilator and discharged alive from the ICU on day 63	Not reported
	Hartman et al 2020 [73]	A man 62 years of age with a history of moderate persistent asthma, sinus bradycardia, chronic obstructive pulmonary disease, and newly diagnosed COVID-19	Not reported	The patient received 217 mL of CP with no other interventions at the time estimated 7 days after onset of symptoms (cough and shortness of breath)	The patient showed rapid improvement in symptoms and electrocardiogram findings, and was discharged 36 hours after the transfusion	Not reported
	Im et al 2020 [74]	A man 68 years of age with severe COVID-19	A donor with ABO blood group A (Rh-positive) incompatible with the patient ABO blood group B (Rh-positive)	250 mL of CP at 16 days after symptom onset for 2 consecutive days with mechanical ventilation and ECMO^d^, steroid, heparinization, and antibiotic treatment	The patient showed clear improvement in respiratory distress and fever symptoms for 3 days after the CP transfusion; discharged without any detectable virus or other complications	No evident acute adverse effect
	Jafari et al 2020 [75]	A woman 26 years of age with a twin pregnancy at 36 weeks and 1 day gestation with confirmed COVID-19	Not reported	One unit of CP was transfused on the sixth day after hospital admission in addition to favipiravir and oxytocine	The patient showed dramatic clinical and radiologic improvements and was discharged 2 weeks after admission with no infection of the newborns	Not reported
	Jiang et al 2020 [76]	A kidney transplant female recipient 70 years of age with immunosuppression; severe COVID-19; and a history of chronic bronchitis, hypertension, and hyperlipidemia	Collected by apheresis from a donor who had recovered from SARS-CoV-2 infection for >14 days, with an ELISA^e^ antibody titer >1:1000	200 mL CP was administered at day 4 and 11 after admission in addition to treatment with moxifloxacin, piperacillin, methylprednisolone, tienam, and fluconazole	The patient’s body temperature became normal and chest CT^f^ was significantly better than at admission, and the patient was discharged on day 30	Not reported
	Karataş et al 2020 [77]	A man 61 years of age with a history of ASCT^g^ for lymphoma with persistent positive tests for SARS-CoV-2 RT-PCR^h^ and fever	Obtained using Trima Accel Automated Blood Collection System from a donor satisfying universal donation criteria and recovered from COVID-19 disease; ELISA IgG titer 13.3	CP transfusion on the 40th day of the infection (dose not specified)	After the CP transfusion, his fever resolved after 3 days. He was discharged from the hospital on the 78th day of hospitalization; viral shedding remained positive as demonstrated by RT-PCR	Not reported
	Kong et al 2020 [79]	A mild COVID-19 male 100 years of age with a 30-year record of hypertension, abdominal aortic aneurysm, cerebral infarction, prostate hyperplasia, and complete loss of cognitive function for the preceding 3 years	Collected via plasmapheresis from a donor who had recovered from COVID-19 for more than 2 weeks and had a SARS-CoV-2 S-RBD^i^–specific IgG titer >1:640	The patient received CP twice: 200 ml on the seventh day of hospitalization and 100 ml on the 11th day of hospitalization	Patient’s viral load decreased significantly, by a factor of ~18, 24 h after the first transfusion of convalescent plasma and then became undetectable after the second, discharged on day 13 of hospitalization	Not reported
	Mira et al 2020 [80]	A male patient 39 years of age with severe COVID-19 and XLA^j^, receiving monthly immunoglobulin replacement therapy	IgG antibodies against either the spike or nucleocapside viral proteins with a titer ≥1:320	200 mL, single dose, on day 23 after admission	After 24 h of infusion, fever ceased without subsequent reappearance and with progressive improvement of asthenia. After 48 h of infusion, no detectable virus in qPCR^k^ from nasopharyngeal exudate	Not reported
	Soleimani and Soleimani 2020 [82]	A woman 30 years of age (gravid 3, parity 2) at her 21 and 2/7 weeks gestation with ARDS^l^ caused by SARS-CoV-2 infection	Not reported	CP was administered in addition to lopinavir/ritonavir and azithromycin and early methyl prednisolone therapy	A mild clinical improvement and decrease in inflammatory markers; normal growth of the fetus	Not reported
	Xu et al 2020 [83]	A man 65 years of age with severe COVID-19	Collected from two convalescent patients; no details provided	CP was given at a 400-mL dose on day 1 and 2 after admission, and hydroxychloroquine was orally administrated for a week	On day 11 after CP transfusion, temperature returned to normal and mechanical ventilation was withdrawn, the RNA test remained positive in throat swab, and CT revealed severe pulmonary lesions	No apparent side effects
	Zhang et al 2020 [84]	A critically ill female 64 years of age with hypertension and diabetes	Collected by apheresis from a male 37 years of age with blood type O at 36 days after symptom onset and 17 days after discharge; CP IgG titer >1:320 by ELISA	200 mL CP on day 17 of hospitalization while receiving invasive mechanical ventilation	The patient did not require mechanical ventilation 11 days after plasma transfusion and was transferred from ICU to a general ward	No adverse event
**Case series**						
	Ahn et al 2020 [85]	A previously healthy man 71 years of age and a woman 67 years of age with a medical history of hypertension, both diagnosed with severe COVID-19	Obtained with Spectra Optia apheresis system from a male donor in his 20s who had recovered from COVID-19 for 21 and 18 days, respectively, and met the blood donor eligibility criteria for plasma donation. ELISA optical density ratio for anti–SARS-CoV-2 IgG was 0.586 and 0.532 (cutoff value 0.22)	A total 500 mL of CP was divided into two doses and given over 1 hour for each dose at 12-hour intervals after 22 days from the onset of symptoms in case 1 and 7 days in case 2	SARS-CoV-2 became negative in both cases: case 1 underwent a tracheostomy and currently was successfully weaned from the mechanical ventilator; case 2 was successfully extubated and discharged from the hospital on day 24	No adverse reaction occurred after the administration of CP
	Abdullah et al 2020 [86]	A male 46 years of age and a male 56 years of age, both with hypertension and severe COVID-19	Collected from a recovered moderate COVID-19 patient after performing necessary investigations for donor plasma (hemoglobin level and viral screen) but not antibody tests	Deteriorated despite supportive care and antiviral therapy: 200 mL of CP at day 3 of hospitalization (day 7 after symptom onset) in case 1; day 10 of hospitalization (day 13 after symptom onset) in case 2	Improve clinically 4 days and 70 h after CP, discharged from the hospital 16 and 21 days after admission with three consecutive negative RT-PCR tests each with at least 24 h apart	Not reported
	Bradfute et al 2020 [87]	12 hospitalized COVID-19 patients (8 males and 4 females) with a median age of 52 (range 39-91) years, 9 obese patients, 10 patients in the ICU, and 2 on the general ward	Collected by apheresis from donors ≥28 days after positive PCR test, with complete recovery from COVID-19 and a median of neutralizing antibody titer of 1:40 (range, undetectable to 1:160)	Patients received one unit (200 mL) CP at a median of 8.5 (range 6-16) days after the onset of symptoms and a median 3.5 (range 1-10) days after hospitalization	Temporal increases in neutralizing antibody titers and IgG/IgM levels, gradual decreases in viral loads, with two deaths within 14 days after CP transfusion	No serious adverse events
	Diorio et al 2020 [88]	Four critically ill children with COVID-19; 14-18 years; female; varied antibody titer levels pretransfusion	Collected from donors proven positive for SARS‐CoV‐2 by a laboratory test; and either ≥14 days from symptom resolution with a repeat negative test for SARS‐CoV‐2 or ≥28 days from symptom resolution without the repeat test. RBD-specific IgG titer <1:160 to >1:6000	200-220 mL of CP at 7-14 days after symptom onset	1 died; 2 showed no clinical improvement; 1 recovered	No emergent adverse events related to CP infusion
	Enzmann et al 2020 [89]	16 critically ill COVID-19 patients with most (12 patients) underlying cardiovascular disease	Not reported	Not reported	In-hospital mortality rate was 31% and median length of hospital stay was 19 (8-36) days	No apparent adverse effects
	Erkurt et al 2020 [90]	26 (8 females and 18 males) severe COVID-19 patients (mean age 67.4, SD 15.5 years)	Collected via apheresis ≥14 days after complete recovery from the eligible blood donors who had mild or moderate COVID-19 with positive antibodies	200 mL of CP was administered at a mean 13.87 (SD 6.5) days after admission in addition to supportive treatment, hydroxychloroquine, azithromycin, and favipiravir	The patients who did not need mechanical ventilation improved with CP treatment, while 6 of 17 patients on mechanical ventilation were dead	No severe adverse reactions
	Fung et al 2020 [56]	4 immune-suppressed patients (males: two were aged 42 years and one was aged 62 years; female: one aged 65 years) with or at risk of progression to severe or life-threatening COVID-19	Collected per FDA^m^ guidance from donors with confirmed COVID-19 and resolution of symptoms within 14-28 days and a negative PCR test or >28 days without a PCR test; ELISA anti–SARS-CoV-2 spike protein IgG titer >1:400	Approximately 200 mL of CP was transfused at 4-27 days following symptom onset	All patients were clinically improved, with 2 discharged home and fully recovered, and 2 discharged to skilled nursing facilities	No adverse reactions
	Gemici et al 2020 [91]	40 consecutive patients (median age 57.5 years and 72.5% male) with severe COVID-19	Collected from eligible blood donors recovered from COVID-19 with negative laboratory results and symptom free for ≥14 days	Patients received a median of 2 (range 1-3) units of CP at median time of 5 days from the diagnosis in addition to antiviral therapy	90% of patients who received CP outside ICU totally recovered at a median of 9 days after the transfusion, and half of the patients treated in ICU were free of mechanical ventilation	No TRALI^n^ or severe allergic reactions
	Hartman et al 2020 [63]	16 (7 female) severe and 15 (3 female) life-threatened patients	Collected from a local donor recruitment and referral program	Dose and timing not reported	Respiratory support requirements began on or about day 7 following CP transfusion, especially in the severe patients	Not reported
	Ibrahim et al 2020 [92]	38 hospitalized, severely (n=16) or critically ill patients (n=22) with confirmed COVID-19 (mean age 63, SD 12 years; 18 female); 31.5% had three or more comorbidities, with 68% having hypertension and 47% having diabetes	Collected by apheresis from adults who were confirmed positive and had recovered from SARS-CoV-2 with negative PCT test for the virus and had total anti–SARS-CoV-2 titer >1:320	ABO-compatible CP was given in two consecutive 200-mL infusions (mean 18.7, SD 9.0) days following symptom onset. Another unit of CP was given to those with undetectable anti–SARS-CoV-2 antibodies.	24 (63%) recovered and were discharged from the hospital, and 14 (37%) died. The survival patients received CP earlier in their course of disease (mean 15.3, SD 6.9 days) and hospital stay (mean 8.4, SD 6.8 days) compared to those who died with mean durations of 24.5 (SD 9.6) days and 16.6 (SD 9.5) days, respectively.	No adverse effects except for a transient transfusion reaction (fever and hematuria) within 2 h of CP infusion in 1 patient
	Bobek et al 2020 [93]	2 critically ill Hungarian patients (males 59 and 72 years of age) with COVID-19, hypertension, and cardiovascular disease	Collected by plasmapheresis from recovered COVID-19 patients who had been asymptomatic for at least 2 weeks, negative PCR tests, and IgG-type antibody detectable by ELISA	3 × 200 mL of CP with the first dose administered on the fourth day of the patient’s ICU mechanical ventilation	Both showed improved oxygenation and inflammatory decreased markers, and were weaned from mechanical ventilation within 2 weeks	No severe adverse effects
	Jin et al 2020 [94]	3 patients (males 10, 24, and 40 years of age) with XLA, hospitalized for COVID-19	CP containing antispike protein titer 1≥:320	Two units of 200 mL ABO-compatible CP were given on days 16, 22, or 44 of illness when there was minimal improvement on other therapies	Various clinical and laboratory improvements including increases in antibody titers; discharged within days after CP transfusion	Not reported
	Joyner et al 2020 [95]	5000 hospitalized adults (median age of 62) with 81% having severe or life-threatening COVID-19 and 66% admitted to ICU	ABO-compatible CP	CP dose of 200-500 mL	The incidence of SAEs^o^ was less than 1%, and the mortality rate at the seventh day after CP transfusion was 14.9%	Of 36 SAEs, 7 and 11 incidents of TACO^p^ and TRALI, respectively, were judged as related to CP transfusion
	Joyner et al 2020 [96]	20,000 hospitalized adults (aged 20-80 years) with severe or life-threatening COVID-19	ABO-compatible CP with no minimum neutralizing antibody titer level donated by recently recovered COVID-19 survivors	CP dose of 200-500 mL	141 SAEs classified as transfusion reactions were reported (<1% of all transfusions); 38 thromboembolic or thrombotic events and cardiac events were related to the transfusion. The mortality rate at the seventh day after transfusion was 13.0%.	Of 141 SAEs, there were 36 reports of TACO, 21 reports of TRALI, and 21 reports of severe allergic transfusion reaction
	Joyner et al 2020 [97]	35,322 hospitalized patients with (or at risk of) severe or life- threatening acute COVID-19 and a diverse representation of gender, age, weight status, race, and ethnicity	Collected from recently recovered COVID-19 survivors without symptoms for ≥14 days, and the antibody levels in the units collected were unknown at the time.	All patients were treated with at least one unit (~200 mL) of CP with the option to administer additional doses if clinically justified in addition to adjunctive COVID-19 medications	A gradient of 7- and 30–day mortality associated with higher IgG levels in CP and early CP transfusion within 3 days of COVID-19 diagnosis	Reported in Joyner et al 2020 [96]
	Liu et al 2020 [117]	3 critically ill male patients with COVID‐19 (42, 56, and 58 years of age; two healthy; one with hypertension)	Collected from COVID-19 survivors who had fully recovered and tested negative for the virus and a total anti–SARS-CoV‐2 IgG titer of 160	Patients were transfused with 200-225 mL CP between 20 and 30 days after disease onset at the critical illness stage in addition to standard care	No therapeutic effect of CP was observed in any of the patients	Not reported
	Maor et al 2020 [99]	49 patients (median age 64.0, IQR 50.5-76.0 years; 35 males) with moderate and severe COVID-19 and comorbidities (diabetes and hypertension) in one-third of the patients	Collected by apheresis procedure from recovered COVID-19 patients eligible for plasma donation and >14 days since the last negative PCR test; neutralizing antibody titer 1:20-1:2560	The first dose of 200 mL CP was transfused at a median of 10.0 (IQR 4.0-14.0) days after PCR diagnosis, followed by a second unit of 200 mL 24 h later, in addition to various standard of care	At day 14 after the first CP dose, 24 patients improved, 9 died, and 13 were ventilated. More patients improved when treated with CP containing higher antibody levels or earlier.	No serious adverse events except that one developed a rash that responded to antihistamine therapy
	Naeem et al 2020 [100]	3 kidney transplant recipients with COVID‐19 treated with CP (1 female 65 years of age admitted to the general medicine service and a female aged 35 years and a male 36 years of age in the ICU)	Collected from donors at local and regional blood centers	One or two units of CP were given on day 2, 4, or 7 after hospital admission, in addition to immunosuppressant/ antiviral/antibiotic	All showed clinical improvement and were discharged 9, 16, and 25 days after hospital admission with no evident infectious complications	1 patient experienced acute chest pain and dyspnea but improved over the following 12-24 h.
	Olivares-Gazca et al 2020 [101]	10 male severe COVID-19 patients with a median age of 53 (range 27-72) years and comorbidities (diabetes, hypertension)	Obtained by apheresis from 5 donors (2 females) with a median age of 35 (range 24-52) years and two negative PCR tests in a 24-h interval 10 days after the resolution of COVID-19 symptoms	Each patient received 200 mL of ABO-compatible CP and other therapies (eg, steroids or hydroxychloroquine)	Improvement in overall respiratory function and clinical condition over a period of 8 days, with 6 discharged and 2 died	No side effects
	Pal et al 2020 [102]	17 critically ill patients (mean age 56, range 24-81 years; 10 males) with COVID‐19 and most patients had multiple medical comorbidities, including 6 with hematological malignancies	Collected from donors 18-56 days following full recovery from COVID-19 with anti–SARS-CoV-2 spike protein IgG titers 1:400-1:6400 as measured by ELISA	A single unit of 200 mL CP was given at an average time of 12 (range 4‐41) days from illness; 3 patients received two units roughly 8 days apart in addition to other COVID-19 treatment and chemotherapy as required	All patients showed a decline in oxygen needs and ventilatory support with most effects seen in patients when CP was administered early in their disease course	No adverse events except a fever during transfusion in 1 patient, resulting in infusion of only 100 mL
	Rahman et al 2020 [103]	13 SOT^q^ recipients (median age 51, range 20‐75 years; 8 males) with severe COVID-19 and comorbidities (eg, hypertension and diabetes)	Collected from eligible blood donors with anti–SARS-CoV‐2 spike protein antibody titers ≥1:320 as measured by ELISA	All patients received two ABO-compatible units of CP, for a total of 500 mL, at a median time of 8 days from symptom onset and additional therapies (hydroxychloroquine alone or in combination with azithromycin, steroids, anticoagulation, and immunosuppression)	8 patients had de-escalating oxygenation support by day 7 post CP. 9 patients were discharged, 1 still hospitalized, and 3 patients died ~3 months after the CP transfusion.	No apparent transfusion-related adverse reactions
	Salazar et al 2020 [104]	25 patients (median age 51 years) with severe or life-threatening COVID-19 and one or more underlying chronic conditions	Obtained from donors eligible according to standard blood donor criteria, confirmed SARS-CoV-2 infection and symptom free for 14 days, and tested negative for SARS-CoV-2 by RT-PCR; ELISA IgG titer ranged from 0 to 1350	One 300-mL dose of CP at a median time of 10 days from symptom onset and concomitant anti-inflammatory and antiviral treatments, and 1 patient received a second dose 6 days after the initial transfusion	By day 14 of CP transfusion, 19 (76%) patients had clinical improvement and 11 were discharged	No adverse events within 24 h after transfusion. 1 patient developed a morbilliform rash 1 day after transfusion that lasted for several days.
	Shen et al 2020 [105]	5 critically ill patients (age range 36-65 years; 2 female) with laboratory-confirmed COVID-19, rapid progression, and continuously high viral load despite antiviral treatment	Obtained from 5 patients who recovered from COVID-19; anti–SARS-CoV-2 IgG titer >1:1000 as determined by ELISA and a neutralization titer >40	ABO-compatible CP was administered at a dose of 200-250 twice (400 mL in total) between 10 and 22 days after admission	Improvement in their clinical status as indicated by declined viral load, body temperature reduction, improved Pa_O2_ /F_iO2_, and chest imaging	Not reported
	Tremblay et al 2020 [106]	24 patients with cancer and severe or life-threatening COVID-19 (median age 69, range 31-88 years; 14 males), some having other comorbidities (eg, hypertension in 15 patients)	Collected via plasmapheresis, spike protein-directed ELISA antibody titers ≥1:320	Two units (250 mL) of ABO-compatible CP were transfused at 3 (IQR 2-7) days from admission in addition to cancer‐directed treatment and COVID-19–specific therapies (hydroxychloroquine, azithromycin, remdesivir, and tocilizumab)	Marked variability in both the timing and degree of improvement or worsening of oxygen requirement; 13 discharged; 10 deaths	3 patients experienced febrile nonhemolytic transfusion reactions
	Wang et al 2020 [108]	5 critically ill COVID-19 patients (median age 56, IQR 50-62 years) admitted to ICU with a persistent (>30 days) positive nucleic acid test for SARS-CoV-2 and underlying chronic comorbidities, including hypertension and diabetes	Collected from the recently cured patients whose antibody titers were above 1:640	200 mL of cross-matching CP was transfused over 15 min initiated at a median of 37 (IQR 34-44) days from the onset of symptoms. In total, 3 patients received 400 mL and the other 2 received 1200 mL; all received antibiotics, antiviral, and anti-inflammatory agents.	Within 6 days after CP therapy, all patients became negative for two consecutive nucleic acid tests. Additionally, 4-9 days following the CP, 3 patients showed resolution of pulmonary lesion. 2 recovered and 3 died.	No adverse reactions
	Wei et al 2020 [107]	2 COVID-19 patients (males aged 50 and 81 years, the latter with type 2 diabetes mellitus, hypertension, and aortic dissection) with long-term positive viral infection	Not reported	One or two 200-mL doses of CP were administered >8 weeks after symptom onset; other therapeutics: interferon, arbidol, chloroquine phosphate, and ritonavir-boosted danoprevir	Substantial improvement as confirmed by CT scan and discharged after three consecutive negative nucleic acid tests	Not reported
	Wu et al 2020 [109]	27 adult patients with prolonged infection for a median of 44 (IQR 30-47) days between symptom onset and last positive test of SARS-CoV-2 before CP therapy (median age 64, IQR 57-72 years; 55.5% males), some with chronic diseases	Collected from donors (without transfusion-related infectious diseases who recovered from COVID-19) >3 weeks after symptom onset and >10 days after discharge; neutralizing antibody titer >1:160	The patients were treated with a median of 400 (IQR 200-600) mL CP at a median of 45 (IQR 3549) days after symptom onset and other therapeutics: antivirals, antibiotics, corticoid, or immunoglobulin	The patients showed pulmonary imaging improvement (within 5-8 days) and viral clearance (18 patients) 15 days after the CP transfusion, and 3 died within 60 days	No transfusion-related adverse reactions
	Xi et al 2020 [110]	3 severe COVID-19 patients with comorbidities (hypertension, liver injury, and hepatitis B)	Collected from 2 recovered patients with high levels of IgG (>30 AU/mL) and IgG titer >1:80	50 mL twice with a 2-day interval and other treatments with noninvasive mechanical ventilation and antiviral, antibacterial drugs, and traditional Chinese medicine	The CT images, blood gas analysis, and symptoms improved after CP therapy. All recovered after 16-18 days of hospitalization.	No adverse event
	Ye et al 2020 [111]	6 laboratory‐confirmed critically ill COVID‐19 patients (mean age 58, SD 16.4 years; 3 male)	Collected from patients at least 3 weeks following disease onset, two consecutive negative RT-PCR tests, and seropositive for anti–SARS‐CoV-2 IgG and IgM	One to three doses of ABO-compatible CP (200 mL/dose) at 6-31 days after admission. Each transfusion was administered over a 30‐minute period.	A resolution of ground‐glass opacities and consolidation in 5 out of 6 patients and an elimination of the virus in 2 in the following days of CP therapy	No adverse events
	Zhang et al 2020 [112]	4 critically ill patients infected with SARS-CoV-2 (age: 31-73 years; 2 male)	Prepared from recovered patients without details	One to eight doses of CP (200-2400 mL in total) 11-41 days after admission in addition to antiviral therapy	The time from transfusion to negative RT-PCR test results ranged from 3 to 22 days. 3 were discharged from the hospital, and 1 remained in ICU up to the time of this writing	No adverse events
	Zeng et al 2020 [113]	8 patients (4 males, median age 65 years) with severe or critical COVID-19; 5 patients had coexisting chronic diseases	Collected from seven donors (median age of 37 years) who had mild or moderate COVID-19 with no comorbidities and were at a median day of 11 from discharge; neutralizing antibody titer 1:255-1:1576	ABO-compatible and cross-matched CP were administered at one (3 patients) or two doses of 100-200 mL of CP within 24 h between 9 and 34 days following the onset of symptoms	6 of 8 patients showed an improvement in oxygen support status within 5 days from CP treatment, partial resolution of pulmonary lesions, and decreased viral load	No adverse events
**Observational (cohort, case-control) studies**						
	Abolghasemi et al 2020 [114]	115 CP treatment group with an average age of 54.4 years, and 74 control group– matched by age, gender, underlying diseases (hypertension and diabetes), and COVID-19 severity	Selected from clinically and laboratory-confirmed recovered patients of COVID-19 who were between the ages of 18-60 years and had no remaining symptoms of COVID-19 infection for at least 14 days; ELISA antibody titer cutoff index >1.1	One unit of 500 mL was infused in <3 days of hospital admission (≤7 days since illness onset), followed by another unit if the patient did not show any improvement after 24 h	More discharged patients (98.2 % vs 78.7 %), shorter hospital stay (9.54 vs 12.88 days), and less requirement for intubation (7% vs 20%) in the CP group than the control group	No adverse effect
	Duan et al 2020 [115]	10 severe COVID-19 patients (6 males and 4 females) with a median age of 52.5 years in comparison with a historic control group of 10 patients matched by age, gender, and severity of the diseases	Collected by apheresis using a Baxter CS 300 cell separator from 10 donor patients who recovered from COVID-19 at 3 weeks after illness and 4 days after discharge and two consecutively negative results of sputum SARS-CoV-2 by RT-PCR assay (1-day sampling interval) neutralization activity of >1:640	One dose (200 mL) of CP at the median time of 16 days from onset of illness in combination with antiviral, antibiotic or antifungal treatment, or glucocorticoid therapy	Improved clinical symptoms and paraclinical criteria within 3 days after CP, varying degrees of absorption of lung lesions for all patients within 7 days, as compared to 3 deaths, 6 cases in stabilized status, and 1 case of improvement in the control group (*P*<.001)	No SAEs or safety events; 1 patient showed an evanescent facial red spot
	Hegerova et al 2020 [116]	20 patients (median age 60, range 29-95 years) with severe or critical COVID-19 treated with CP under an expanded access protocol, as compared with 20 matched controls with regard to age, number of comorbidities, and severity of illness	Collected from patients aged from 29 to 79 years who recovered from COVID-19 (symptom free) for >28 days without hospitalization, most showing anti–SARS-CoV-2 IgG	One unit of ABO-compatible CP was administered early at the median time of 2 (IQR 1-4.3) days from hospitalization and additional therapies (eg, azithromycin and hydroxychloroquine)	Improved laboratory and respiratory parameters in patients following CP infusion, similar to those in controls but with lower mortality (2 vs 6 deaths)	No adverse events
	Liu et al 2020 [117]	39 hospitalized patients (mean age 55, SD 13 years; 25 males) with severe to life-threatening COVID-19 received CP transfusion in comparison with a cohort of retrospectively matched controls (n=156)	Collected by plasmapheresis from donors with antispike antibody titers ≥1:320 as measured by ELISA	Two units (250 mL each unit) of ABO-type matched were infused over 1-2 hours at the median time of 4 days after admission in addition to a variety of inpatient pharmacotherapies	More likely improvements in supplemental oxygen requirements by posttransfusion day 14, improved survival, compared to control patients, especially for nonintubated patients	No significant transfusion-related morbidity or mortality
	Perotti et al 2020 [118]	46 moderate to severe COVID-19 patients (mean age 63, SD 12 years), with 19 (41%) having two or more comorbidities, in comparison with a control cohort of 23 consecutive patients	Collected using a Trima Accel blood collection device from eligible COVID-19 recovered patients with 2 consecutive negative tests for SARS‐CoV‐2, followed by pathogen reduction; neutralization titers ≥1:80	24 patients received one unit of plasma, 21 received two units, and 1 patient received 3 units after having symptoms for 2 weeks, with most having been treated with antibiotics, hydroxychloroquine, and anticoagulants	3 out of 46 patients (6.5%) died within 7 days (at 1, 4, and 6 days), lower than 30% in the control, and showed improved respiratory function (PaO_2_ /FiO_2_), chest radiogram, laboratory parameters (CRP^r^, Ferritin, LDH^s^, viral load), and weaning from mechanical ventilation	Five serious adverse events occurred in 4 patients.
	Rasheed et al 2020 [119]	49 early-stage (no more than 3 days in ICU) critically ill COVID-19 patients randomized to receive CP or not (21 and 28 patients, respectively, matched in terms of age, sex, and comorbidities)	Collected from healthy donors younger than 50 years who recovered from moderate COVID-19 and had a IgG index ≥1.25 as measured by ELISA	400 mL of CP were transfused over 2 hours in addition to standard of care in the control group	CP-treated patients showed reduced duration of infection in about 4 days, a lower death rate (1/21 vs 8/28), and higher levels of SARS-CoV-2 IgG and IgM 3 days after CP transfusion compared to the control group	No adverse events except that 1 patient developed mild skin redness and itching that lasted for 1 hour after CP; resolved by antihistamine injection
	Roger et al 2020 [120]	64 patients with symptom onset ≤10 days prior to admission and supplemental oxygen (but not invasive ventilation) within 48 h of hospitalization versus a matched control group of 177 patients for all cause in-hospital mortality and rate of hospital discharge at day 28	The SARS-CoV-2 antibody content in CP was assessed retrospectively with 13% of the units below the cutoff for a positive antibody index	3 of 64 patients received one and the remainder received two units of CP at a median of 7 (IQR 5-9) days after symptom onset	No significant difference in the risk of in-hospital mortality or overall rate of hospital discharge between the two groups, except for a significantly increased hospital discharge rate among patients 65 years or older	2 patients had TRALI reactions associated with the first unit of CP, and 1 had TACO approximately 3 h after transfusion of the second unit of CP
	Salazar et al 2020 [121]	136 severe or life-threatening COVID-19 patients treated with CP versus 215 propensity score-matched patients to assess the efficacy of CP transfusion compared to standard of care	Collected from donors who had been asymptomatic for more than 14 days and had negative SARS-CoV-2 RT-PCR tests at the time of plasmapheresis; antispike IgG antibody titers ≥1:1350 as measured by ELISA	The majority of patients received one and some patients reviewed two units of CP due to worsening COVID-19 conditions	Patients treated by CP with IgG titer ≥1:1350 within 72 h of hospital admission had decreased mortality within 28 days	Reported in Joyner et al 2020 [95]
	Xia et al 2020 [122]	1568 severe or critical COVID-19 patients, most with comorbidities, among whom 1430 patients (median age of 63 years; 50% male) only received standard treatment and 138 patients (median age of 65 years; 56% male) also received ABO-compatible CP	Not reported	200-1200 mL of CP were transfused at a median of 45 days of symptom onset (1 week to ≥8 weeks from symptom onset to CP therapy)	Compared to that in the standard treatment group, there was a reduced mortality rate (2.2% vs 4.1%), lower admission to ICU (2.4% vs 5.1%), and improved respiratory symptoms of severe patients as evaluated by SCSS^t^	No significant differences in cardiac, liver, and renal functions before and after CP therapy, except for a decrease in total bilirubin and 3 patients with minor allergic reactions (pruritus or erythema) during the transfusion
	Xiao et al 2020 [123]	18 patients with severe and critical COVID-19 divided into two groups with no significant differences in age, gender, and basic clinical data: one with CP transfusion (n=6) and the other without CP transfusion (n=12)	Collected from donors between age 18-55 years who had fully recovered from COVID-19 without symptoms for 2 weeks and ≥4 weeks from symptom onset; anti–SARS-CoV-2 IgG titers >1:160	200~500 mL (4~5 mL/kg body weight) of CP were transfused	No difference between the two groups of patients in terms of ventilator and ECMO weaning time, time for viral clearance, and hospitalization	Not reported
	Zeng et al 2020 [124]	21 critically ill patients with COVID-19 and respiratory failure: 6 patients (median age of 61.5 years; 5 males) in the CP group versus 15 patients (median age of 73 years; 11 males) in a control group with no significant differences in demographic and clinical features	200-400 mL obtained from each young adult individual who had recovered from COVID-19 for 1-2 weeks and was negative for SARS-CoV-2 RNA and IgM testing, and positive for IgG testing before donation	A median volume of 300 mL CP was transfused at a median of 21.5 days after viral shedding was first detected	All CP-treated patients tested negative for SARS-CoV-2 RNA within 3 days after infusion versus 26.7% in the control group, but 5 patients eventually died with a longer survival period, suggesting treatment should be initiated earlier	No immediate or noticeable adverse effects
**RCT^u^**						
	Gharbharan et al 2020 [125]	86 hospitalized patients (median age of 63 years; 72% male) randomized at 1:1 for standard of care therapy with and without CP	Collected from donors confirmed with an RT-PCR SARS-CoV-2 infection and were asymptomatic for at least 14 days; neutralizing antibodies titer ≥1:80 determined by a SARS-CoV-2 plaque reduction neutralization test	One unit of 300 mL ABO-compatible CP was transfused on the day of inclusion followed with the second plasma unit after 5 days for patients with persistent positive RT-PCR tests	There was no difference in day-60 mortality, hospital stay (*P*=.68), or day-15 disease severity (*P*=.58) between CP-treated patients and patients on standard care. The study was discontinued due to high neutralizing antibody titers at hospital admission in the majority of the study population.	No plasma-related serious adverse events were observed
	Li et al 2020 [126]	103 patients (median age 70 years; 60 males, 58.3%) with severe and life-threatening COVID-19 randomized to receive CP in addition to standard treatment (n=52) or standard treatment (antiviral medications, antibacterial medications, steroids, human immunoglobulin, Chinese herbal medicines, and other medications) alone (control; n = 51)	Collected based on routine plasma collection procedures via plasmapheresis from adults aged 18-55 years that were suitable for blood donation, initially diagnosed with COVID-19 but with 2 negative PCR results from nasopharyngeal swabs (at least 24 h apart) prior to hospital discharge, discharged for ≥2 weeks from the hospital, and had no persisting COVID-19 symptoms. CP S-RBD–specific IgG titer ≥1:640 correlating to serum neutralization titre of 1:80	ABO-compatible CP was transfused at approximately 4-13 mL/kg of recipient body weight and at approximately 10 mL for the first 15 minutes, which was then increased to approximately 100 mL per hour with close monitoring	More clinical improvement occurred within 28 days in the CP group than in the control group among those with severe disease (91.3% vs 68.2%; *P*=.03) but not for those with life-threatening disease (20.7% vs 24.1%; *P*=.83). There was a higher negative conversion rate of viral PCR at 72 hours in the CP group than in the control group (87.2% vs 37.5%; *P*<.001).	2 patients in the CP group experienced adverse events within hours after transfusion that improved with supportive care

^a^CP: convalescent plasma.

^b^PCR: polymerase chain reaction.

^c^ICU: intensive care unit.

^d^ECMO: extracorporeal membranous oxygenation.

^e^ELISA: enzyme-linked immunosorbent assay.

^f^CT: computed tomography.

^g^ASCT: autologous stem cell transplantation.

^h^RT-PCR: real-time polymerase chain reaction.

^i^S-RBD: spike protein receptor-binding domain.

^j^XLA: X-linked agammagloblulinemia.

^k^qPCR: quantitative polymerase chain reaction.

^l^ARDS: acute respiratory distress syndrome.

^m^FDA: Food and Drug Administration.

^n^TRALI: transfusion-related acute lung injury.

^o^SAE: serious adverse event.

^p^TACO: transfusion-associated circulatory overload.

^q^SOT: solid organ transplant.

^r^CRP: C-reactive protein.

^s^LDH: lactate dehydrogenase.

^t^SCSS: six-category scale score.

^u^RCT: randomized controlled trial.

### Patient Demographics

A total of 36,379 patients, with most patients (n=35,322) from a single study [97], have been treated with CP in all clinical studies included in this review. There is a patient heterogeneity across the clinical studies in terms of age (ranging from infant [81] and 6 [70] to 100 years [79]), gender, and different underlying diseases, in particular hypertension and diabetes [114,122,124,294]. Some case studies investigated CP therapy for COVID-19 in patients who were immune compromised or deficient [56,80,94,100,103,125].

A few studies reported the antibody titers of patients before CP transfusion, which varied from undetectable IgG RBD antibody levels (<1:50 serum dilution) to extremely high levels (1:25,600) [88]. Studies suggested that patients with low antibody levels may benefit more from CP therapy [88,125].

### Donor Selection and CP Antibody Titer

Most individuals with previous laboratory-diagnosed SARS-CoV-2 infection developed measurable antibody responses and neutralizing antibodies. There is evidence for a significant decline in neutralizing antibody levels over time [280].

Studies suggest that the efficacy of CP depends on the antibody levels of the donor plasma and CP, with high antibody levels possibly conferring immediate immunity to recipients [122]. One key factor associated with CP therapy is the neutralizing antibody titer, and when the infused plasma has a high antibody titer, it may be of the greatest benefit [88,97,99,113]. Hence, it may be a prerequisite to find eligible donors who have high levels of neutralizing antibody.

Prior smaller studies have reported a variety of titer cut-offs [105,115]. The FDA has recommended that CP with a virus neutralizing antibody titer of ≥1:160 be used for therapeutic transfusion [295]. Recently, the FDA has updated its EUA to limit the authorization to the use of high titer CP for the treatment of hospitalized patients with COVID-19 early in the disease course and to those hospitalized who have impaired humoral immunity and cannot produce an adequate antibody response, and include additional tests to be used in the manufacture of COVID-19 CP [296]. Studies have reported the levels of CP antibody titer, ranging from no minimum neutralizing antibody titer level [96] to 1:640 [115], and an even wider range of RBD-specific IgG titer, from <1:160 to >1:6000 within the same study [88].

There was substantial heterogeneity in the antibody response among potential CP donors, but sex, age, and hospitalization emerged as factors that can be used to identify individuals with a high likelihood of having strong antiviral antibody levels [297]. In vitro testing of CP showed a tendency of higher neutralizing antibody titers from donors with increased disease severity, of advanced age, and of male sex; however, the clinical relevance of this difference needs to be investigated [109,270,276,277,283,284]. Moreover, pooling CP samples from many donors may prove more effective for increasing and standardizing anti–SARS-CoV-2 neutralizing antibody titers [19].

In addition, CP collection efforts should be organized around the temporal dynamics of the immune response to viral clearance and a rise in neutralizing antibody titer, with a recommended window for plasma collection beginning at 4 weeks after the resolution of symptoms and narrowing rapidly by 12 weeks [165].

### Timing and Dose

One key factor associated with CP efficacy is the optimal treatment time point [115]. The phase of the disease that this treatment modality may be most beneficial is still a matter of some debate, with early versus intermediate-late stages of the cytokine storm reaction being associated with acute respiratory distress syndrome or other severe disease complications [298].

There was no therapeutic effect from CP treatment on severely or critically ill patients with COVID-19 more than 2 weeks after the onset of disease as reported by Liu et al [117]. However, CP therapy has been limited to patients with severe or critical COVID-19. The majority of patients were severe or critically ill with COVID-19, with only a few mild cases [79,90,109].

Similar to most viral illnesses, viremia in COVID-19 peaks in the first week of infection, and the primary immune response develops by days 10-14, which is followed by virus clearance. Therefore, transfusion of CP at the early stage of disease theoretically should be more effective [114,121,124]. CP appears to be of greater clinical benefit when administered early in the course of disease than delaying transfusion under the development of severe disease [63,108]; in principle, the course of disease does not exceed 3 weeks [67]. Studies have found that, regardless of COVID-19 severity at time of transfusion, patients that received CP earlier in their course of disease showed lower mortality, more rapid viral clearance, and shorter hospital stays [92,113].

Based on the current findings, CP treatment should be given to patients with COVID-19 at the right phase or severity of illness and at the right time point. It is known that most patients with mild COVID-19 can recover without treatment, and CP may be an improper therapy for those patients. For patients with end-stage COVID-19, treatment with CP may be unable to avert a poor outcome, as demonstrated by the current findings [108,124,294]. Therefore, CP treatment may be more beneficial if used in patients who are potentially critically ill with COVID-19 at an early stage of the disease. Thus, early recognition of patients with COVID-19 who are likely to become critically ill is important for timely treatment with CP [124].

This is in line with one of the first published RCTs of CP, in which Li and colleagues [126] found that clinical improvement was limited to those without life-threatening disease, with 91% improvement in the plasma group compared to 68% in the control arm [294]. A large multicenter study involving 35,322 patients found significant reductions in 7- and 30-day mortality with early use of CP containing high levels of SARS-CoV-2–specific IgG antibodies in a subset of patients [97].

Transfusion volume ranged from 2x50 mL [110] to 8x300 mL [112]. Total antibody dose could be calculated as the transfused volume of CP multiplied by SARS-CoV-2 neutralizing antibody titer. CP dose has also been recognized as a key characteristic that may influence CP-associated outcomes [187]. One study showed that patients transfused with 400 mL of CP tended to turn faster to viral clearance than those who received 200 mL of CP [113].

### Safety

All studies that assessed adverse events have reported no or minimal adverse events [102,206]. Of major interest is one of the first large trials published so far—concerning the safety of 5000 recipients—that has identified only limited and nonunexpected transfusion complications [95]. The case series study focused on the safety of CP transfusion in COVID-19 reported that, out of 5000 patients, there were 7 transfusion-associated circulatory overload (TACO), 11 transfusion-related lung injury (TRALI), and 3 severe allergic reactions. However, the reported low incidence of serious adverse effects might be due to an extremely short time frame of observation (4 hours) [194]. The latest update of the study involving 20,000 hospitalized adults with severe or life-threatening COVID-19 further demonstrated low adverse events because of the treatment, with 36 TACO, 21 TRALI, 21 severe allergic reactions, and 38 transfusion-related thromboembolic events [96]. Consistently, other studies reported no to minimal adverse events. Half of the case reports that assessed the safety of CP did not indicate any adverse events or complications related to its use. One case series study reported 5 serious adverse events in 4 out of 46 patients [118]. The controlled studies reported 15 adverse events out of 695 patients. Overall, among a total of 20,749 patients reported with safety data, the incidence of adverse events related to CP transfusion was less than 0.8%, comparable or even lower than the incidence of adverse events related to plasma transfusions in other clinical settings [299]. There has been no evidence so far of antibody-mediated enhancement of disease in patients with COVID-19 treated with CP despite the concern that this might be a possibility in the presence of reactive but nonneutralizing antibodies against SARS-CoV-2 [170].

Although it is not yet clear whether the SARS-CoV-2 virus is transmitted by blood [300], donor selection criteria in compliance with existing policies and routine procedures should be met and pathogens reduction by solvent- or detergent–based treatments or light-based methods (especially for noncovered or detected in screening tests) should be performed in each donated plasma product as a standard for any plasma production [157,230]. Ultraviolet light and riboflavin used in the pathogen reduction process could effectively reduce SARS-CoV-2 in plasma and blood products without decreasing the quality of the blood products [301]. More studies have shown that the pathogen reduction processes did not alter neutralizing antibodies [156,272].

### Outcomes

These were measured by SARS-CoV-2 negative PCR tests, improvements of clinical symptoms assessed by respiratory distress and fever, computed tomography, time to death, length of hospital stay, and mortality at discharge.

All case reports showed either viral load decrease/clearance or different extents of improvements of clinical symptoms with no mortality. Preliminary evidence from case reports and case series is favorable, as significant clinical and biochemical improvement and hospital discharge have been reported.

COVID-19 severity and underlying diseases affected the outcome of CP treatment. A patient with lymphoma who underwent autologous stem cell transplantation showed persistent SARS-CoV-2 viral shedding for 74 days, even with the administration of CP [77]. On the other hand, 1 study reported that 2 patients with long-term positive viral infection for 8 weeks showed substantial improvement after treatment with CP and ritonavir-boosted danoprevir [107]. Similarly, another study showed that CP therapy could rapidly reduce viral loads in more than half of 27 patients with prolonged positivity of SARS-CoV-2 for a median of 44 days after symptom onset [109]. It should be noted that most of these patients had mild COVID-19 symptoms.

Studies demonstrated that CP could effectively improve the respiratory symptoms of severe patients and help them wean from oxygen support. However, patients in extremely critical or life-threatening conditions could not benefit from CP [63,122,124,294].

The case series reported a mortality rate of 24.4% in 35,666 patients, mainly from 1 study with 35,322 patients [97]. The case-control and randomized controlled studies included a total of 2289 patients in the control group and 695 patients in the CP group, and reported a total of 219 (9.6%) and 63 (9.1%) deaths in each group, respectively. The number of patients and the mortality rates varied remarkably among these studies, from 6 [124] to 1430 patients [122] and from 0% [115] to 93.3% [124], respectively. The mortality at discharge [114] or at 28-day posttransfusion [121,294] have been reported as a primary outcome. Some studies showed improved survival for the CP group compared to its control [115,117,122], more clinical improvements [115,117], and viral clearance [115,124]. The efficacy of CP on mortality, length of hospital stay, clinical improvement, and viral clearance was further analyzed by meta-analysis of controlled studies, as presented later.

### Quality Assessment of Clinical Studies

As indicated in Table 3, 52 clinical studies showed overall weak quality, 9 had moderate quality, and 1 had strong quality. Patients often had underlying medical conditions (hypertension, diabetes). Case reports and series with limited number of patients were considered weak for selection of participants (high risk of selection bias). Some studies included only males with a total of 3 patients [117] or only pediatric patients with fewer than 4 children [70,88] and therefore were judged to be weak for sample selection. Studies that targeted a specific group (eg, older populations, median age >60 years) were rated with moderate selection bias [122,124,125,294], while studies that selected patients with a broad range of ages and balanced gender and comorbidities [114,121] were ranked as strong.

**Table 3:** Quality assessment components and their rankings for clinical studies evaluated using the Effective Public Health Practice Project tool.

Studies	Patient selection	Study design	Confounders	Blinding	Data collection methods	Withdraws/ dropouts	Overall
Al Helali et al 2020 [65]	Weak	Weak	Weak	Weak	Weak	Strong	Weak
Anderson et al 2020 [66]	Weak	Weak	Weak	Weak	Weak	Strong	Weak
Bao et al 2020 [67]	Weak	Weak	Weak	Weak	Weak	Strong	Weak
Cinar et al 2020 [68]	Weak	Weak	Weak	Weak	Moderate	Strong	Weak
Clark et al 2020 [69]	Weak	Weak	Weak	Weak	Weak	Strong	Weak
Figlerowicz et al 2020 [70]	Weak	Weak	Weak	Moderate	Moderate	Strong	Weak
Grisolia et al 2020 [71]	Weak	Weak	Weak	Weak	Moderate	Strong	Weak
Hahn et al 2020 [72]	Weak	Weak	Weak	Weak	Moderate	Strong	Weak
Hartman et al 2020 [73]	Weak	Weak	Moderate	Weak	Weak	Moderate	Weak
Im et al 2020 [74]	Weak	Weak	Weak	Weak	Weak	Strong	Weak
Jafari et al 2020 [75]	Weak	Weak	Weak	Weak	Weak	Strong	Weak
Jiang et al 2020 [76]	Weak	Weak	Weak	Weak	Moderate	Strong	Weak
Karataş et al 2020 [77]	Weak	Weak	Weak	Weak	Weak	Strong	Weak
Kong et al 2020 [79]	Weak	Weak	Weak	Moderate	Moderate	Strong	Weak
Mira et al 2020 [80]	Weak	Weak	Moderate	Weak	Moderate	Strong	Weak
Soleimani and Soleimani 2020 [82]	Weak	Weak	Weak	Weak	Weak	Strong	Weak
Xu et al 2020 [83]	Weak	Weak	Weak	Weak	Weak	Strong	Weak
Zhang et al 2020 [84]	Weak	Weak	Weak	Weak	Moderate	Strong	Weak
Ahn et al 2020 [85]	Weak	Weak	Weak	Weak	Strong	Strong	Weak
Abdullah et al 2020 [86]	Weak	Weak	Weak	Weak	Weak	Strong	Weak
Bradfute et al 2020 [87]	Moderate	Weak	Weak	Weak	Moderate	Strong	Weak
Diorio et al 2020 [88]	Weak	Weak	Weak	Weak	Strong	Strong	Weak
Enzmann et al 2020 [89]	Weak	Weak	Weak	Weak	Weak	Strong	Weak
Erkurt et al 2020 [90]	Moderate	Weak	Weak	Weak	Moderate	Strong	Weak
Fung et al 2020 [56]	Weak	Weak	Weak	Weak	Moderate	Strong	Weak
Gemici et al 2020 [91]	Moderate	Weak	Weak	Weak	Moderate	Strong	Weak
Hartman et al 2020 [63]	Moderate	Weak	Weak	Weak	Weak	Strong	Weak
Ibrahim et al 2020 [92]	Moderate	Weak	Weak	Weak	Strong	Strong	Weak
Bobek et al 2020 [93]	Weak	Weak	Weak	Weak	Moderate	Strong	Weak
Jin et al 2020 [94]	Weak	Weak	Weak	Weak	Moderate	Strong	Weak
Joyner et al 2020 [95]	Strong	Weak	Weak	Weak	Weak	Strong	Weak
Joyner et al 2020 [96]	Strong	Weak	Weak	Weak	Moderate	Strong	Weak
Joyner et al 2020 [97]	Strong	Weak	Moderate	Weak	Strong	Strong	Weak
Liu et al 2020 [98]	Weak	Weak	Weak	Weak	Moderate	Strong	Weak
Maor et al 2020 [99]	Moderate	Weak	Weak	Weak	Strong	Strong	Weak
Naeem et al 2020 [100]	Weak	Weak	Weak	Weak	Moderate	Strong	Weak
Olivares-Gazca et al 2020 [101]	Weak	Weak	Weak	Weak	Moderate	Strong	Weak
Pal et al 2020 [102]	Moderate	Weak	Weak	Weak	Moderate	Strong	Weak
Rahman et al 2020 [103]	Weak	Weak	Weak	Weak	Moderate	Strong	Weak
Salazar et al 2020 [104]	Weak	Weak	Weak	Weak	Strong	Strong	Weak
Shen et al 2020 [105]	Moderate	Weak	Weak	Weak	Strong	Strong	Weak
Tremblay et al 2020 [106]	Moderate	Weak	Weak	Weak	Moderate	Strong	Weak
Wang et al 2020 [108]	Weak	Weak	Weak	Weak	Strong	Strong	Weak
Wei et al 2020 [107]	Weak	Weak	Weak	Weak	Weak	Strong	Weak
Wu et al 2020 [109]	Moderate	Weak	Weak	Weak	Strong	Strong	Weak
Xi et al 2020 [110]	Weak	Weak	Weak	Weak	Weak	Moderate	Weak
Ye et al 2020 [111]	Moderate	Weak	Weak	Weak	Strong	Strong	Weak
Zhang et al 2020 [112]	Moderate	Weak	Weak	Weak	Moderate	Strong	Weak
Zeng et al 2020 [113]	Weak	Weak	Weak	Weak	Strong	Strong	Weak
Abolghasemi et al 2020 [114]	Strong	Moderate	Moderate	Weak	Strong	Strong	Moderate
Duan et al 2020 [115]	Moderate	Moderate	Weak	Weak	Strong	Strong	Weak
Hegerova et al 2020 [116]	Moderate	Moderate	Weak	Weak	Strong	Strong	Weak
Liu et al 2020 [117]	Moderate	Moderate	Moderate	Moderate	Strong	Strong	Moderate
Perotti et al 2020 [118]	Moderate	Moderate	Moderate	Weak	Strong	Moderate	Moderate
Rasheed et al 2020 [119]	Moderate	Strong	Strong	Weak	Moderate	Strong	Moderate
Roger et al 2020 [120]	Moderate	Moderate	Moderate	Weak	Strong	Strong	Moderate
Salazar et al 2020 [121]	Strong	Moderate	Moderate	Weak	Strong	Strong	Moderate
Xia et al 2020 [122]	Moderate	Moderate	Moderate	Weak	Moderate	Strong	Moderate
Xiao et al 2020 [123]	Weak	Moderate	Moderate	Weak	Moderate	Strong	Weak
Zeng et al 2020 [124]	Moderate	Moderate	Moderate	Weak	Moderate	Strong	Moderate
Gharbharan et al 2020 [125]	Moderate	Strong	Strong	Weak	Strong	Strong	Moderate
Li et al 2020 [126]	Moderate	Strong	Strong	Moderate	Strong	Strong	Strong

With respect to the study design, case reports and series were considered to be weak; case-control studies and RCTs were determined to be moderate and strong, respectively. The confounders for case reports and series studies were ranked weak given the uncontrolled nature of these studies involving other therapeutic treatments and supportive care and the use of other treatment regimens, including antiviral medications along with CP transfusion. Two different analytical methods were used to control for confounding in 1 case series study [97] subsequently determined to be of moderate risk for confounders. This component was ranked to be strong for RCTs and moderate for case-control studies, except for 1 study by Duan et al [115] given the uncertain characteristics of participants selected into the intervention group and the use of a historical control group.

As CP treatment was not blinded to either outcome assessors or study patients in most studies, the blinding component was judged to be weak except for the RCT by Li et al [126], where the evaluation of clinical outcomes was performed by an investigator who was blind to the treatment.

If there was no detailed CP therapy in terms of CP collection, neutralizing antibody or anti–SARS-CoV-2 IgG titers, timing and dose of the treatment, and valid measures of clinical outcomes, the data collection methods of the study were deemed to be weak. Some case reports did not provide any information for CP donators, antibody titers, and adverse events [66,67].

There were no dropouts in the case reports and case series. One case series study where all patients were followed up for only 7 days [118] was ranked as moderate. In the RCT reported by Gharbharan et al [125], all 86 patients had been followed for at least 15 days after inclusion, and 75 and 32 patients for at least 30 and 60 days, respectively.

Both RCTs were terminated prematurely due to the concerns over the potential benefit of CP in the study population with high neutralizing antibody titers (≥1:160) at baseline [125] and the lack of patients with COVID-19 to reach the planned recruitment target of 200 patients [294], resulting in an underpowered study sample size.

### Meta-analyses

**Figure 2:**
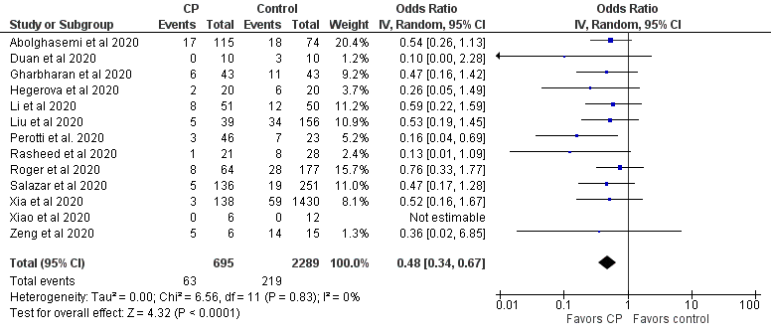
Efficacy of CP treatment on mortality in COVID-19 patients. Data from 13 controlled clinical trials were pooled using an inverse variance method and analyzed using a random-effects model. Odds ratios and 95% CIs were used as statistical measures for mortality as a dichotomous outcome. CP: convalescent plasma.

**Figure 3:**
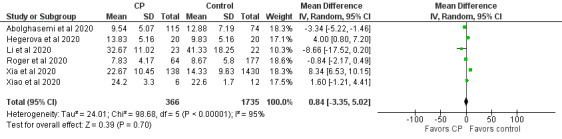
Efficacy of CP treatment on length of hospital stay in COVID-19 patients. Data from 6 controlled clinical trials were pooled using an inverse variance method and analyzed using a random-effects model. Means and SDs were the statistical measures used to describe the length of hospital stay. CP: convalescent plasma.

**Figure 4:**
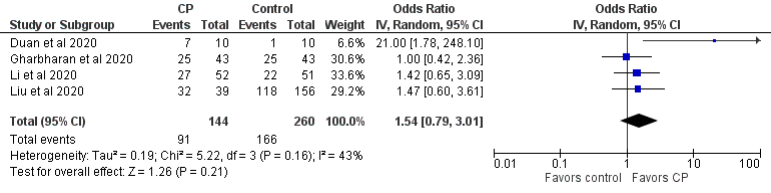
Efficacy of CP treatment on clinical improvement in COVID-19 patients. Data from 4 controlled clinical trials were pooled using an inverse variance method and analyzed using a random-effects model. Odds ratios and 95% CIs were used as statistical measures for clinical improvement as a dichotomous outcome. CP: convalescent plasma.

**Figure 5:**
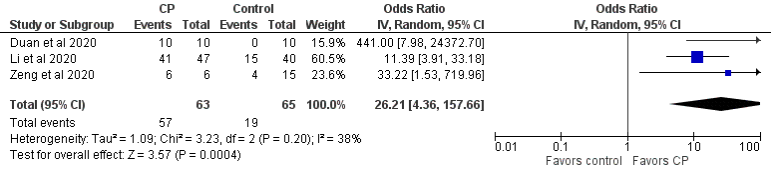
Efficacy of CP treatment on viral clearance in COVID-19 patients. Data from 3 controlled clinical trials were pooled using an inverse variance method and analyzed using a random-effects model. Odds ratios and 95% CIs were used as statistical measures for viral clearance as a dichotomous outcome. CP: convalescent plasma.

Figures 2-5 summarize the statistical analyses of pooled results from the controlled clinical studies addressing the efficacy of CP treatment for COVID-19. We found 13 controlled articles (2 RCTs and 11 cohort studies) assessing mortality, with a total of 695 and 2289 patients in the CP and control groups, respectively. CP reduced the mortality by half in COVID-19 (OR 0.48, 95% CI 0.34-0.67; I^2^=0), as demonstrated in the forest plot (Figure 2).

However, fewer studies were available to assess the effects of CP treatment on the length of hospital stay, clinical improvement, and viral clearance. We identified only 6 studies (1 RCT and 5 cohort studies) reporting the length of hospital stay, with a total of 366 and 1735 patients in the CP and control groups, respectively (Figure 3). These studies had significant heterogeneity (*P*<.001; I^2^=95%) and, when combined, did not show any effects of CP treatment on the length of hospital stay (mean difference 0.84, 95% CI –3.35 to 5.02 days).

Similarly, 4 studies (2 RCTs and 2 cohort studies) assessed the clinical improvement with the number of patients in both CP and control groups. As depicted in Figure 4, a larger portion of the patients in the CP group showed improved clinical status compared to that in the control, but the difference was not statistically significant (OR 1.54, 95% CI 0.79-3.01; I^2^=43%).

Based on the 3 studies (1 RCT and 2 cohort studies) with a total of 63 and 65 patients in the CP and control groups, respectively, we found that the use of CP increased the viral clearance significantly (OR 26.21, 95% CI 4.36-157.66; I^2^=43%) as shown in Figure 5.

Except for the high heterogeneity among the studies on assessing the length of hospital stay (I^2^=0.98; *P*<.001), the heterogeneity among the studies assessing the clinical improvement and viral clearance was mild (I^2^=43%, *P*=.16 and I^2^=38%, *P*=.20, respectively). Furthermore, since the included studies on the efficacy of CP treatment for mortality are homogenous (I^2^=0; *P*=.99), the overall effect on the mortality from the meta-analysis seems to be conclusive.

### Mechanisms of Action

The biological basis for efficacy of CP entails the transfer of specific antiviral immunoglobulins (antibodies) and other bioactive substances in the plasma of patients in the convalescent phase of COVID-19 infection [233,302]. In theory, administration of CP containing high levels of polyclonal neutralizing antibodies (comprised mainly of IgG, with smaller amounts of IgM, IgA) can confer immediate pathogen-specific protection by inhibiting viral infection in a susceptible person [303]. However, findings suggest considerable variation in antibody titers and the duration of protective anti–SARS-CoV-2 IgG and IgM immunity observed in recovered CP donors [304,305]. A recent population-based study of humoral immune responses to SARS-CoV-2 demonstrated that >90% of people who recovered from COVID-19 were seropositive on virus-specific pan-immunoglobulin assays by day 25, and hospitalized patients seroconverted more frequently than nonhospitalized people. Furthermore, anti–SARS-CoV-2 antibody titers remained stable in recovered patients for the next 2 months, suggesting a durable immunoglobulin response [306]. Aside from CP, pooled human immunoglobulins may also be prepared from plasma as a concentrated antibody-containing solution to be administered as intravenous, subcutaneous, or intramuscular immunoglobulin. These pooled plasma-derived immunoglobulin products benefit from the polyclonal response of each individual donor and from the interindividual variability in such responses [307]. In addition, purified, high-titer hyperimmune immunoglobulin formulations can be obtained from vaccinated or convalescing donors, which have known levels of plasma-derived neutralizing antibodies that may prove valuable against COVID-19 [33,207,225].

Although not fully elucidated, the protective mechanisms of CP are based on direct and indirect antiviral activities, including antibody neutralization of viral infectivity [233,307]. In the case of SARS-CoV-2 pathogenesis, the viral spike glycoprotein is critical to the dissemination and pathogenesis of the virus [308]. The spike protein mediates binding of SARS-CoV-2 to host cell ACE-2 surface receptors, thereby acting as the first step in cellular entry and infection. Several lines of evidence from studies of SARS-CoV and CoV-2 show that infected hosts produce neutralizing antibodies directed against the RBD of the homotrimeric spike protein and can block infection by preventing viral entry and subsequent replication [309]. Other beneficial immune effects of CP are thought to include enhanced antibody-dependent cellular cytotoxicity, complement activation, and phagocytosis, along with restoration of the vascular endothelial glycocalyx [34,200]. Moreover, a majority of convalescent patients display robust antiviral SARS-CoV-2–specific T cell responses, with enhanced in vivo priming and expansion of CD8+ cytotoxic T cells and a higher frequency of CD4+ memory T cells in those who recovered from severe COVID-19, which may provide long-term antiviral protection even if antibodies wane [310]. Therefore, T cells could help to control SARS-CoV-2 infection and serve as correlates of protective antiviral immunity [311].

As new strains of SARS-CoV-2 with several dominant mutations in the spike protein have been identified recently, crucial questions associated with the possible reinfection of recovered patients and the efficiencies of vaccines designed based on early epidemic strains have arisen [22]. Recent findings show that sera collected from convalescent COVID-19 patients in early 2020 vaccinated with RBD-based vaccines efficiently neutralize viral variants of D614G and B.1.1.7 but weakly neutralize those of 501Y.V2, suggesting a warning to recovered patients and developed vaccines [312]. These results show that, as mutations accumulate in the RBD, spike proteins may acquire an antigenic shift that enable SARS-CoV-2 variants with loss-of-neutralization potency in vitro against emerging variants and eventually resist the current vaccines. Therefore, intensive monitoring of virus mutations and timely adjustments to the spike sequences of designed vaccines and updated antibody cocktail therapies, targeting highly conserved regions, are required to control the viral pandemic [313].

## Discussion

### Main Findings

This systematic review summarizes a variety of evidence on the use of CP for treatment of COVID-19. Though the focus of this review was to identify and assess the quality of clinical studies reporting CP treatment for COVID-19, the broad search strategy identified a large number of studies related to various aspects of CP use, highlighting substantial research in this field. The data on this topic is being rapidly generated and reported. Most are commentary and review articles and protocol or guidance descriptions on the theme of CP treatments for COVID-19. The main findings according to each group of articles dealing with COVID-19 CP were:

1. Clinical studies: Overall, there were significant variations among the studies regarding the study design and population, the timing of initiation of CP transfusion, dosage and neutralizing antibody titer, and concomitant therapy. The quality of the current evidence on the use of CP for COVID-19 was low. However, there is a widespread belief that CP should be used given that no other efficacious treatment is currently available.
2. Commentary articles: This category mainly consisted of commentary and letter to the editor in addition to a few editorials and perspectives that collectively supported the use of CP for COVID-19 and suggested further clinical trials.
3. Review: The volume and the pace of the clinical trials launched to evaluate the safety and efficacy of CP against COVID-19 reflects the need for high-quality evidence for the therapy to be practiced by clinicians.
4. Protocol and guideline: This category of literature showed the importance for establishment of a CP production and storage transfusion program in a public health care network and a decision-making framework; the requirements applicable to plasma donors; and the standards for preparation, qualification, storage, distribution, and control of product use.
5. In vitro testing of CP: A variety of tests have been developed to measure the levels of CP antibodies. Generally, two methods have been most used to determine antibody titers of CP: ELISA for IgG and IgM, and neutralization assay for neutralizing antibodies. ELISA-based antibody titers can correlate well with neutralizing titers.

Our meta-analysis of controlled studies showed significant reduction in mortality by CP therapy in comparison to controls. Similar meta-analysis of the efficacy of CP therapy on different types of infectious disease found a 44% reduction in the mortality of patients with COVID-19 [208]; a 25% reduction in other severe acute respiratory infections [33]; and a 32% reduction in SARS-CoV infection, severe influenza, and Ebola infection [209]. In contrast, the meta-analysis from 4 RCTs on CP treatment for influenza infection (n=572 patients) showed no convincing effects on deaths [206]. Another recent systematic review of 1 RCT and 3 controlled nonrandomized studies of CP therapy in patients with COVID-19 reported a potential reduction in mortality, time to death, and improvement of clinical symptoms but was unable to provide any opinion regarding the efficacy of CP treatment for COVID-19 due to paucity in quantitative synthesis [207].

Our meta-analysis showed no effect of CP on the length of hospital stay (mean difference), which is consistent with another meta-analysis of 3 RCTs for the effect of CP on the length of hospitalization in other severe respiratory viral infections, as reported by Devasenapathy et al [206]. Other systematic reviewers reported mixed results of both reduced length of hospital stay and no effects on the length of hospitalization in SARS-CoV infection, severe influenza, and Ebola infection [209], suggesting that the effectiveness of CP in reducing hospital length of stay might be dependent on early administration of the therapy, and its use as prophylaxis is more likely to be beneficial than treating severe disease [33]. However, the optimal timing and dosage of CP therapy remains to be defined.

The insignificant effect of CP on the improvements of clinical COVID-19 symptoms is comparable to another systematic review and meta-analysis of 5 studies with a total of 259 patients with COVID-19, showing more clinically improved patients treated with CP than no CP treatment but was not statistically significant (OR 2.06, 95% CI 0.8-4.9; I^2^=44%) [208]. In contrast, the meta-analysis of 9 controlled and uncontrolled studies showed improved clinical status of patients with COVID-19 when compared to baseline (ROM 0.53, 95% CI 0.36-0.79; *P*<.01; n=149) [147].

The significant increase in the viral clearance is also consistent with the other meta-analysis of 2 studies with a total of 144 patients, suggesting that the use of CP helps in viral clearance significantly [208], and with the meta-analysis of 9 controlled and uncontrolled studies showing reduced viral loads [147].

Various tools have been developed for quality assessment involving slightly different components and ranking criteria [314]. We used the EPHPP tool as it can be used for all types of clinical studies. This is a generic tool used to evaluate a variety of intervention study designs such as RCTs, before-and-after, and case-control studies [62]. A study has shown differences in quality assessment for RCTs between the EPHPP and the Cochrane Collaboration Risk of Bias tool [315]. Overall, clinical studies and systematic reviews have confirmed that CP caused few or no serious adverse events with low-quality evidence.

Consistent with other reviews [207,208], our quality appraisal showed that the present studies on the efficacy of CP are generally of low quality, although there are certain agreements and discrepancies between our assessment and others on the overall quality of case and randomized controlled studies on the use of CP for COVID-19, as different assessment tools have been used. Only 1 high-quality (low risk of bias in the underlying study results) RCT by Li et al [126] was identified in our assessment using the EPHPP tool, which is in agreement with the assessment in the systematic review by Sarkar et al [208], but was rated to be unclear in another systematic review by Piechotta et al [207], even though both reviews used the same Cochrane risk-of-bias tool (RoB 2.0) for the RCT.

The overall quality of the case-control studies in our assessment lies in between the risk of bias assessed by other two systematic reviews conducted by Piechotta et al [207] and Sarkar et al [208]. Specifically, the study by Duan et al [115] was considered weak in our quality assessment but was critical as assessed by Piechotta et al [207] and moderate risk of bias by Sarkar et al [208] in their reviews. The case-control study reported by Liu et al [117] was of moderate quality in our assessment but was critical and had a low risk of bias as assessed by Piechotta et al [207] and Sarkar et al [208], respectively, using the same Risk of Bias in Non-randomized Studies–of Interventions. The case-control study reported by Zeng et al [124] was moderate in our assessment, agreeing with the assessment in the systematic review by Sarkar et al [208], but was rated to be a critical risk of bias in the systematic review by Piechotta et al [207]. In addition to controlled and randomized studies, EPHPP could be used to assess the quality of case reports and series studies [62]. The overall quality of all case reports and series were weak based on our assessment.

Considering the promising evidence from existing clinical data, there is a clear need for RCTs on large patient numbers to evaluate the efficacy of CP therapy. Apart from sample size and the noncomparative, nonrandomized study design, numerous limitations hamper the interpretation of the aforementioned studies, such as the superimposition of effects mediated by other antiviral treatments, antibiotics, and glucocorticoids administered concomitantly with CP. As a whole, these studies indicate that patients receiving transfusions earlier than 14 days post infection may benefit from CP treatment [228,230].

### Limitations

There are 2 systematic reviews and meta-analysis to appraise the literature on CP therapy for patients with COVID-19. However, this review covers the latest literature as of the date of our manuscript submission and provides insights about various aspects for the subject on the use of CP for COVID-19 that needs further investigation. The primary limitation of this review is that most data identified are nonrandomized (only 2 out of 64 clinical studies were randomized, with only 1 being of high quality), and therefore, confounding is highly inevitable. Furthermore, study populations, interventions, and measured outcomes have important clinical and methodological heterogeneity, which reflects an overall low to moderate quality of evidence identified by the appropriate quality assessment tool.

Publication bias may be another potential limitation given that the majority of early clinical studies on COVID-19 lacked original data, and those that did were rushed and did not include the appropriate measures to reduce bias [316]. Among the 243 papers included in this review, 32.5% (n=79) were commentaries, 18.9% (n=46) were reviews, and 7.8% (n=19) were protocols that did not contain any new data. We then evaluated the quality of the original clinical studies using the validated tool and found that more than 80% (52/64) were at risk of bias, mainly because of few participants, unrepresentative patient selection, poor study design, no control of confounders, and no blinding.

### Future Directions

We summarized various aspects of the evidence on the use of CP in patients with COVID-19. However, important gaps in knowledge remain. Notably, the following areas require further investigation.

Well-designed prospective observational studies, preferentially RCTs, with well-defined characteristics for both CP donors and recipients are warranted to answer questions concerning the effects on mortality or other important clinical outcomes such as improvement in symptoms and respiratory status. The placebo or control should include standard-of-care or normal fresh frozen plasma. The plasma exchange has shown therapeutic effects for severe COVID-19 acute respiratory distress syndrome with multiple organ failure [317].

In vitro testing showed variable or diverse neutralizing antibody titers among individual donors, suggesting that an adequate pooling strategy of plasma units from different donors could reduce the variability of neutralizing antibody titers of CP and compensate deviations of individual antibody titers [289]. Clinical studies on the safety and efficacy of pooled CP should be conducted.

The COVID-19 pandemic has substantially reduced the national ability to provide blood products for medical care in an emergency [318], which further highlights the need to secure a stockpile of blood products with a long shelf life (eg, freeze-dried plasma) to be self-sufficient in a national crisis. Current CP protocols specify that, once thawed, CP may be stored for up to 5 days at 4 °C, similar to that of fresh frozen plasma. A recent study has demonstrated long-term stability of anti–SARS-CoV-2 spike antibodies in donor CP for 42 days when stored under refrigerated conditions [291]. There will be a need to stockpile freeze-dried CP for future waves of the pandemic for several years. Additionally, global concern over the potential for future waves of infection to occur before effective vaccines or drug therapies are available has many looking at other biological sources for large-scale production of neutralizing SARS-CoV-2 antibodies. Taking this into consideration, we are developing COVID-19 convalescent freeze-dried plasma. As this is a pooled plasma product of 10 donors, we also hypothesize that convalescent freeze-dried plasma will have higher anti–SARS-CoV-2 neutralizing antibody titers and activity than single donor CP. As well, this product may be administered in a hypertonic solution for those patients who cannot tolerate large volume CP transfusions.

### Conclusions

There is still limited evidence but accumulating interest in CP treatment for COVID-19. The theoretical reasons for the likely efficacy of passive immunization, the urgent need felt by clinicians worldwide for effective treatment options for COVID-19, and the promising results offered mainly by retrospective clinical studies must be balanced against the lack of efficacy in the RCTs of CP and hyperimmune globulin therapy in severe influenza and COVID-19.

CP may be of greatest benefit for patients who are early in their illness and have not yet generated endogenous antibodies, and when the infused CP has a high antibody titer. Recurring observations suggested that treatment with CP within 4-5 days of symptom onset might be more effective than later treatment.

Our systematic review and analysis emphasizes the low quality of clinical studies. These studies could provide important lessons that should inform the planning of adequately powered and properly designed RCTs to evaluate the promise of CP therapy for patients with COVID-19.

Future research is necessary to fill the obvious knowledge gaps regarding CP treatment for patients with COVID-19. In brief, we offered recommendations around the need for a large-scale properly designed RCT, the potential prophylactic use of CP, selection criteria for both CP donors and recipients, development of antibodies with higher potency than CP, and freeze-dried CP as a long-term strategy against the pandemic.

## Appendix

Multimedia Appendix 1 Supplemental Table 1.

## Abbreviations

ACE-2: angiotensin-converting enzyme 2
CP: convalescent plasma
ELISA: enzyme-linked immunosorbent assay
EPHPP: Effective Public Health Practice Project
EUA: Emergency Use Authorization
FDA: Food and Drug Administration
Ig: immunoglobulin
MERS-CoV: Middle East respiratory syndrome–related coronavirus
OR: odds ratio
PCR: polymerase chain reaction
PRISMA: Preferred Reporting Items for Systematic Reviews and Meta-Analyses
RBD: receptor binding domain
RCT: randomized controlled trial
ROM: ratio of mean
SARS-CoV: severe acute respiratory syndrome–related coronavirus
TACO: transfusion-associated circulatory overload
TRALI: transfusion-related lung injury
