# Correction: Convalescent Plasma for the Prevention and Treatment of COVID-19: A Systematic Review and Quantitative Analysis

**DOI:** 10.2196/31554

**Published:** 2021-06-30

**Authors:** Henry T Peng, Shawn G Rhind, Andrew Beckett

**Affiliations:** 1 Defence Research and Development Canada Toronto Research Centre Toronto, ON Canada; 2 St. Michael’s Hospital Toronto, ON Canada; 3 Royal Canadian Medical Services Ottawa, ON Canada

In “Convalescent Plasma for the Prevention and Treatment of COVID-19: A Systematic Review and Quantitative Analysis” (JMIR Public Health Surveill 2021;7(4):e25500), some symbols (<, >, and ~) were missing in 13 places in the paper due to an XML conversion error. The following corrections have been made:

- In Table 1, row “Pseudovirus capture assay...,” column “Summary,” the passage “(neutralizing antibody titers 1:16 to 1:1024)” has been corrected to “(neutralizing antibody titers <1:16 to >1:1024)”.
- In Table 2, row “Al Helali et al…,” column “Outcomes/main findings,” the passage “few days after CP transfusion and negative PCR^b^ test for COVID-19 in 48 h” has been corrected to “few days after CP transfusion and negative PCR^b^ test for COVID-19 in <48 h”.
- In Table 2, row “Jiang et al…,” column “Details of CP,” the passage “Collected by apheresis from a donor who had recovered from SARS-CoV-2 infection for 14 days, with an ELISA^e^ antibody titer 1:1000” has been corrected to “Collected by apheresis from a donor who had recovered from SARS-CoV-2 infection for >14 days, with an ELISA^e^ antibody titer >1:1000.”
- In Table 2, row “Kong et al…,” column “Outcomes/main findings,” the passage “Patient’s viral load decreased significantly, by a factor of 18” has been corrected to “Patient’s viral load decreased significantly, by a factor of ~18.”
- In Table 2, row “Fung et al…,” column “Details of CP,” the passage “without a PCR test; ELISA anti–SARS-CoV-2 spike protein IgG titer 1:400” has been corrected to “without a PCR test; ELISA anti–SARS-CoV-2 spike protein IgG titer >1:400.”
- In Table 2, row “Joyner et al…,” column “Interventions and comparisons,” the passage “All patients were treated with at least one unit (200 mL) of CP with the option to administer” has been corrected to “All patients were treated with at least one unit (~200 mL) of CP with the option to administer.”
- In Table 2, row “Rahman et al…,” column “Outcomes/main findings,” the passage “…1 still hospitalized, and 3 patients died 3 months after the CP transfusion.” has been corrected to “...1 still hospitalized, and 3 patients died ~3 months after the CP transfusion.”
- In Table 2, row “Shen et al…,” column “Details of CP,” thee passage “Obtained from 5 patients who recovered from COVID-19; anti–SARS-CoV-2 IgG titer 1:1000 as determined by ELISA and a neutralization titer 40” has been corrected to “Obtained from 5 patients who recovered from COVID-19; anti–SARS-CoV-2 IgG titer >1:1000 as determined by ELISA and a neutralization titer >40.”
- In Table 2, row “Wei et al…,” column “Interventions and comparisons,” the passage “One or two 200-mL doses of CP were administered 8 weeks after symptom onset” has been corrected to “One or two 200-mL doses of CP were administered >8 weeks after symptom onset.”
- In Table 2, row “Abolghasemi et al…,” column “Details of CP,” the passage “ELISA antibody titer cutoff index 1.1” has been corrected to “ELISA antibody titer cutoff index >1.1.”

The correction will appear in the online version of the paper on the JMIR Publications website on June 30, 2021, together with the publication of this correction notice. Because this was made after submission to PubMed, PubMed Central, and other full-text repositories, the corrected article has also been resubmitted to those repositories.

