# Supplementary material for: Convalescent Plasma for the Prevention and Treatment of COVID-19: A Systematic Review and Quantitative Analysis
Source: JMIR Public Health Surveill. 2021 Apr 7;7(4):e25500. doi: 10.2196/25500 (PMC8245055; doi:10.2196/25500)
Supplement: Multimedia Appendix 1 [file publichealth_v7i4e25500_app1.docx]

**Convalescent Plasma for the Prevention and Treatment of COVID-19: A Systematic Review and Quantitative Analysis**

Henry T. Peng, PhD^1^, Shawn G. Rhind, PhD^1^, Andrew Beckett, MD^2^

^1^Defence Research and Development Canada, Toronto Research Centre, Toronto, Ontario, Canada

^2^St. Michael’s Hospital, Toronto, Ontario and Royal Canadian Medical Services, Ottawa, Canada

**Supplemental Table S1.** Summary of literature in details. The articles were classified into five types: 64 clinical studies (20 case reports, 31 case series, 11 case-controlled and two RCTs), 79 commentary articles, 46 reviews, 19 guidance and protocols, and 35 in vitro testing of CP antibodies.

| **Article type** | **Number of articles** | **Group** | **Summary** | **References** |
| --- | --- | --- | --- | --- |
| Clinical studies | 64 |  | A 55-year-old previously healthy male with severe COVID-19 was successfully treated with CP after treatment with favipiravir and hydroxychloroquine, enoxaparin | Al Helali et al. 2020 [[1](#_ENREF_1)] |
|  |  |  | A 35-year-old critically ill obstetric patient with COVID-19 was successfully treated with remdesivir and convalescent plasma | Anderson et al. 2020 [[2](#_ENREF_2)] |
|  |  |  | A 38-year-old critically ill man infected by SARS-CoV-2 and suffered from cerebral hemorrhage was treated with 150 ml plasma of type A Rh positive COVID-19–convalescent patient | Bao et al. 2020 [[3](#_ENREF_3)] |
|  |  |  | A myelodysplastic COVID-19 patient with disseminated tuberculosis was treated with CP in combination with antiviral and anti-cytokine drugs | Cinar et al. 2020 [[4](#_ENREF_4)] |
|  |  |  | A 76-year-old woman with persisting COVID‐19 following therapeutic lymphocyte depletion was treated with CP in combination of lopinavir/ritonavir, prednisone | Clark et al. 2020 [[5](#_ENREF_5)] |
|  |  |  | A six-year-old severe COVID-19 girl transfused with CP (inactivated using methylene blue) with anti SARS-CoV-2 IgG at a titer of 1:700 once in a 200 mL dose | Figlerowicz et al. 2020 [[6](#_ENREF_6)] |
|  |  |  | A 29-year-old woman at 24 2/7 weeks of gestation was treated with CP after antibiotic therapy (ceftriaxone and azithromycin) and prophylactic low-molecular-weight heparin | Grisolia et al. 2020 [[7](#_ENREF_7)] |
|  |  |  | A patient with severe COVID-19 on prolonged mechanical ventilation was successfully treated with CP | Hahn et al. 2020 [[8](#_ENREF_8)] |
|  |  |  | A 62-year-old man with history of moderate persistent asthma, sinus bradycardia, chronic obstructive pulmonary disease and newly diagnosed COVID-19 was successfully treated with CP | Hartman 2020 [[9](#_ENREF_9)] |
|  |  |  | A 68-year old man with COVID-19 who received 250 mL of ABO-incompatible CP for 2 consecutive days with mechanical ventilation | Im et al. 2020 [[10](#_ENREF_10)] |
|  |  |  | A 26-year-old woman with a twin pregnancy at 36 week and one day gestation with confirmed COVID-19 received one plasma transfusion obtained from cured COVID-19 patients in addition to favipiravir | Jafari et al. 2020 [[11](#_ENREF_11)] |
|  |  |  | A 70-year old kidney transplant female recipient with severe COVID-19 was treated with CP and moxifloxacin, piperacillin, methylprednisolone, tienam, fluconazole | Jiang et al. 2020 [[12](#_ENREF_12)] |
|  |  |  | A 61-year-old man with a history of autologous stem cell transplantation (ASCT) for lymphoma was treated with CP with an anti-SARS-CoV-2 IgG titer of 13.3 | Karataş et al. 2020 [[13](#_ENREF_13)] |
|  |  |  | A 41-year-old male with acute myeloid leukemia (AML) and COVID-19 treated with remdesivir and CP | Khan et al. 2020 [[14](#_ENREF_14)] |
|  |  |  | A 100-year-old male diagnosed with COVID-19 and other health problems was successfully treated with CP (SARS-CoV-2 S-RBD-specific IgG titer of > 1:640) | Kong et al. 2020 [[15](#_ENREF_15)] |
|  |  |  | A 39-year-old male severe COVID-19 patient with x-linked agammagloblulinemia receiving monthly immunoglobulin replacement therapy was treated with 200-mL CP (antibodies against either the spike or nucleocapside viral proteins IgG with a titer ≥1/320) on day 23 after admission | Mira et al. 2020 [[16](#_ENREF_16)] |
|  |  |  | A 3.1-kg term 9-week-old female with congenital heart disease and COVID-19 refractory to remdesivir was treated successfully with CP | Rodriguez et al. 2020 [[17](#_ENREF_17)] |
|  |  |  | A 30- year-old woman (gravid 3, parity 2) at her 21 and 2/7 weeks gestation with ARDS caused by SARS-CoV-2 infection was treated with CP in addition to lopinavir/ritonavir and azithromycin and early methyl prednisolone therapy | Soleimani and Soleimani 2020 [[18](#_ENREF_18)] |
|  |  |  | A 65-year-old Chinese man with severe COVID-19 received CP transfusion twice and oral hydroxychloroquine administration for a week | Xu et al. 2020 [[19](#_ENREF_19)] |
|  |  |  | A 64-year-old critically ill female with hypertension and diabetes received CP (IgG titer>1:320) while receiving invasive mechanical ventilation | Zhang et al. 2020 [[20](#_ENREF_20)] |
|  |  | Case series | Two COVID-19 patients (a healthy 71-year-old man and a 67-year-old woman with a medical history of hypertension) presented severe pneumonia with ARDS showed a favorable outcome after use of CP in addition to systemic corticosteroid | Ahn et al. 2020 [[21](#_ENREF_21)] |
|  |  |  | Two male cases (46- and 56-year-old) with hypertension and severe COVID-19 were transfused with 200 mL of CP despite supportive care and antiviral therapy | Abdullah et al. 2020 [[22](#_ENREF_22)] |
|  |  |  | 12 hospitalized COVID-19 patients (8 males and 4 females) with a median age of 52 years (range, 39–91 years) were transfused with CP to evaluate neutralizing antibody levels in CP or in recipients and clinical outcomes. | Bradfute et al. 2020 [[23](#_ENREF_23)] |
|  |  |  | Four critically ill children with COVID-19, 14–18 years were treated with 200–220 mL of CP in addition to SARS-CoV-2 directed therapies | Diorio et al. 2020 [[24](#_ENREF_24)] |
|  |  |  | 16 critically ill COVID-19 patients appeared refractory to other therapies or supportive care, CP was thus pursued. | Enzmann et al. 2020 [[25](#_ENREF_25)] |
|  |  |  | 26 severe COVID-19 patients received 200 mL CP in addition to supportive treatment, hydroxychloroquine, azithromycin and favipiravir | Erkurt et al. 2020 [[26](#_ENREF_26)] |
|  |  |  | Four immunosuppressed patients (two 42-year-old and one 62-year-old males, one 65-year-old female) with or at risk of progression to severe or life-threatening COVID-19 were transfused with CP collected as per FDA guidance | Fung et al. 2020 [[27](#_ENREF_27)] |
|  |  |  | 40 consecutive patients with severe COVID-19 received a median of 2 units of CP as well as antiviral therapy | Gemici et al. 2020 [[28](#_ENREF_28)] |
|  |  |  | 16 severe and 15 life-threatened COVID-19 patients received CP transfusion | Hartman et al. 2020 [[29](#_ENREF_29)] |
|  |  |  | 38 hospitalized, severely or critically ill patients with confirmed COVID-19 were treated with CP to assess its safety and efficacy | Ibrahim et al. 2020 [[30](#_ENREF_30)] |
|  |  |  | Two critically ill patients with COVID-19 infection were treated with 3 × 200 mL CP in addition to antiviral agents and a full scale of supportive care. | Ilona et al. 2020 [[31](#_ENREF_31)] |
|  |  |  | Three patients with X-linked agammaglobulinemia hospitalized for COVID-19 failed supportive treatment but recovered after CP therapy | Jin et al. 2020 [[32](#_ENREF_32)] |
|  |  |  | 5,000 hospitalized adults with severe or life threatening COVID-19, with 66% in the intensive care unit, were treated with ABO-compatible CP with safety as the primary outcome | Joyner et al. 2020 [[33](#_ENREF_33)] |
|  |  |  | 20,000 hospitalized adults with severe or life-threatening COVID-19 were treated with approximately 200 to 500 mL of  ABO-compatible CP with safety as the primary outcome | Joyner et al. 2020 [[34](#_ENREF_34)] |
|  |  |  | 35,322 hospitalized patients with (or at risk of) severe or life threatening acute COVID-19 were treated with CP with seven and thirty-day mortality as main outcomes | Joyner et al. 2020 [[35](#_ENREF_35)] |
|  |  |  | Three critically ill male patients with COVID-19 were treated by CP in addition to standard care | Liu et al. 2020 [[36](#_ENREF_36)] |
|  |  |  | 49 patients with moderate and severe COVID-19 were treated with CP in addition to various standard of care | Maor et al. 2020 [[37](#_ENREF_37)] |
|  |  |  | Three kidney transplant recipients with COVID-19 were treated with CP in addition to immunosuppressant/antiviral/antibiotic (one admitted to the general medicine service, two in ICU) | Naeem et al. 2020 [[38](#_ENREF_38)] |
|  |  |  | Ten male patients with severe COVID-19 and a median age of 53 years (range 27-72) received ABO-compatible CP and other therapies e.g., steroids hydroxychloroquine | Olivares-Gazca et al. 2020 [[39](#_ENREF_39)] |
|  |  |  | 17 critically ill patients with COVID‐19, including six with haematological malignancies, were treated with CP with marked clinical improvement in addition to other COVID-19 treatment and chemotherapy as required | Pal et al. 2020 [[40](#_ENREF_40)] |
|  |  |  | 13 solid organ transplant (SOT) recipients with severe COVID-19 received CP with additional therapies (hydroxychloroquine alone or in combination with azithromycin, steroids, anticoagulation, and immunosuppression) | Rahman et al. 2020 [[41](#_ENREF_41)] |
|  |  |  | 25 severe COVID-19 patients treated with CP anti-inflammatory and anti-viral treatments to evaluate safety and clinical status at day 14 post-transfusion | Salazar et al. 2020 [[42](#_ENREF_42)] |
|  |  |  | Five critically ill patients with COVID-19 treated with CP (IgG titer>1000, neutralization titer>40) between 10 and 22 days after admission in addition to various antiviral agents and steroids | Shen et al. 2020 [[43](#_ENREF_43)] |
|  |  |  | 24 patients with cancer and severe or life-threatening COVID-19 treated with CP in addition to cancer‐directed treatment and COVID-19 specific therapies (hydroxychloroquine, azithromycin, remdesivir, tocilizumab) | Tremblay et al. 2020 [[44](#_ENREF_44)] |
|  |  |  | Two COVID-19 patients with long-term positive viral infection for > 8 weeks treated with CP in addition to recombinant human interferon, arbidol, chloroquine phosphate, ritonavir-boosted danoprevir | Wei et al. 2020 [[45](#_ENREF_45)] |
|  |  |  | Five critically ill COVID-19 patients with a persistently positive nucleic acid test for SARS-CoV-2 received CP therapy | Wang et al. 2020 [[46](#_ENREF_46)] |
|  |  |  | 27 patients with mild COVID-19 symptom, but prolonged positivity of SARS-CoV-2 for a median 44 (30–47) days between symptom onset and last positive test of SARS-CoV-2 RNA before CP therapy, | Wu et al. 2020 [[47](#_ENREF_47)] |
|  |  |  | Three severe patients received CP in addition to antiviral therapy, antibacterial therapy and traditional Chinese medicine | Xi et al. 2020 [[48](#_ENREF_48)] |
|  |  |  | Six critically ill patients with COVID-19 received the transfusion of ABO‐compatible CP besides anti‐viral drug arbidol, leading to improvement in patient’s symptoms and ameliorating radiologic abnormalities | Ye et al. 2020 [[49](#_ENREF_49)] |
|  |  |  | Four critically ill patients with SARS-CoV-2 infection received CP in addition to supportive care (antiviral drugs, mechanical ventilation) | Zhang et al. 2020 [[50](#_ENREF_50)] |
|  |  |  | Eight patients (four males and four females) with critical or severe COVID-19 were administered one or two transfusions of CP | Zeng et al. 2020 [[51](#_ENREF_51)] |
|  |  | Observational (cohort, case-control) studies | 115 CP treatment group and 74 control group to compare outcomes including all-cause mortality, total hospitalization days and patients’ need for intubation between the two groups | Abolghasemi et al. 2020 [[52](#_ENREF_52)] |
|  |  |  | 10 severe COVID-19 patients treated with CP as an addition to maximal supportive care and antiviral agents in comparison with a control | Duan et al. 2020 [[53](#_ENREF_53)] |
|  |  |  | 20 patients with severe or critical COVID-19 were treated with one unit of ABO-compatible CP under an expanded access protocol, as compared with 20 matched controls | [Hegerova et al](http://dx.doi.org/10.1182/blood.2020006964). 2020 [[54](#_ENREF_54)] |
|  |  |  | 39 hospitalized patients with severe to life-threatening COVID-19 received CP transfusion in comparison with a cohort of retrospectively matched controls | Liu et al. 2020 [[55](#_ENREF_55)] |
|  |  |  | 46 moderate to severe COVID-19 patients treated with CP to assess safety and 7-day hospital mortality in comparison with a control cohort of 23 consecutive patients | Perotti et al. 2020 [[56](#_ENREF_56)] |
|  |  |  | 31 out of 49 early-stage critically-ill COVID-19 patients received CP, while the rest 28 namely control group, did not receive, to compare clinical and laboratory outcomes | Rasheed et al. 2020 [[57](#_ENREF_57)] |
|  |  |  | 64 patients received CP at a median of 7 days after symptom onset and were compared to a matched control group of 177 patients for all cause in-hospital mortality and rate of hospital discharge at day 28 | Roger et al. 2020 [[58](#_ENREF_58)] |
|  |  |  | 316 severe and/or life-threatening COVID-19 patients treated with CP versus 215 propensity score-matched patients to assess the efficacy of CP transfusion compared to standard of care | Salazar et al. 2020 [[59](#_ENREF_59)] |
|  |  |  | 138 patients received ABO-compatible CP versus 1,430 patients in standard-treatment group to  evaluate the effectiveness, safety, and indications of the CP transfusion therapy for severe or critical COVID-19 patients | Xia et al. 2020 [[60](#_ENREF_60)] |
|  |  |  | 18 patients with severe and critical COVID-19 were divided to two groups with no significant differences in age, gender and basic clinical data except one with CP transfusion (n=6) and the other without CP transfusion (n=12) | Xiao et al. 2020 [[61](#_ENREF_61)] |
|  |  |  | Six critically ill patients with COVID-19 treated with CP to evaluate its efficacy in comparison with 15 patients in a control group | Zeng et al. 2020 [[62](#_ENREF_62)] |
|  |  | RCT | 86 hospitalized patients randomized at 1:1 ratio for standard of care therapy with and without CP. The primary outcome was day-60 mortality. | Gharbharan et al. 2020 [[63](#_ENREF_63)] |
|  |  |  | 103 patients were enrolled and randomized to receive CP in addition to standard treatment (n=52) or standard treatment alone (n=51), the primary outcome of time to clinical improvement within 28 days | Li et al. 2020 [[64](#_ENREF_64)] |
| Commentary (Correspondence, Editorial, Letter to the Editor, Opinions, Perspectives, Viewpoints) | 79 | Positive | Viewpoint of CP therapy as an effective, safe, and feasible therapeutic option for COVID-19 based on historical and current data for its treating coronaviruses including SARS-CoV-2, mechanism of action and possible drawbacks | Alghamdi and Abdel-Moneim et al. 2020 [[65](#_ENREF_65)] |
|  |  |  | Based on the evidence of CP efficiency in treating human coronaviruses, in favor of clinical use and evaluation as a method for treating COVID-19. | Alzoughool and Alanagreh, 2020 [[66](#_ENREF_66)] |
|  |  |  | Potential use of CP and stem cells for treating COVID-19 based on their unique immunomodulatory properties and emerging science and clinical trials | Borlongan et al. 2020 [[67](#_ENREF_67)] |
|  |  |  | Comparison of mortality rates between two groups of COVID-19 ICU patients: group 1 treated in a local hospital without CP and group 2 from three published studies involving CP showed that CP therapy reduced the death rate with an odds ratio value of 0.03223 (95% confidence interval 0,0018 – 0,5777). | Cantore and Valente 2020 [[68](#_ENREF_68)] |
|  |  |  | Comments on the first RCT of CP in COVID-19 that showed clinical improvement in severely ill patients compared to standard treatment, limited by the sample size due to lack of patient enrolment | Casadevall 2020 [[69](#_ENREF_69)] |
|  |  |  | Based on previous use of CP against coronaviruses and risks and benefits analysis, recommending emergency use of convalescent sera to treat individuals with early symptoms and prevent disease in those exposed and preparations as soon as possible. | Casadevall 2020 [[70](#_ENREF_70)] |
|  |  |  | Encouraging evidence for the usefulness of CP in treating COVID-19 from a large observational study that showed reduction in mortality at days 7 and 14. | Casadevall and Tobian [[71](#_ENREF_71)] |
|  |  |  | Based on previous use of CP against coronaviruses, supporting studies of the safety and efficacy of CP transfusion in COVID-19 patients | Chen 2020 [[72](#_ENREF_72)] |
|  |  |  | Based on previous use, immediate availability of CP and five clinical trials of CP therapy for COVID-19, with limitations in mind, supporting the efficacy of CP therapy for COVID-19 especially in patients with moderate to severe symptoms | Cheraghali 2020 [[73](#_ENREF_73)] |
|  |  |  | Suggestions of a potential role for antithrombin in the treatment of COVID-19 with CP | Gazzaruso et al. 2020 [[74](#_ENREF_74)] |
|  |  |  | The importance of published experience and the pending establishment of efficacy to support the use of CP as standard treatment instead of experimental therapy for COVID-19 | Farhat et al. 2020 [[75](#_ENREF_75)] |
|  |  |  | A variety of aspects to be considered for the optimal use of CP donations for COVID-19 including manufacturing turn-around time, safety, efficacy. cost and the logistics of storage, distribution and administration | Focosi et al. 2020 [[76](#_ENREF_76)] |
|  |  |  | Based on data from previous use of CP against coronaviruses and COVID-19 CP case series, supporting CP transfusion in COVID-19 patients, particularly at an early stage of the disease | Franchini 2020 [[77](#_ENREF_77)] |
|  |  |  | Suggestions for production of CP including donor selection (completely recovered by at least 14 days, titers ≥1:160), serologic tests for the titer of anti–SARS-CoV-2 neutralizing antibodies (neutralization test preferred to ELISA), CP collection (plasmapheresis procedures) | Franchini et al. 2020 [[78](#_ENREF_78)] |
|  |  |  | Comments on CP donation protocol and ongoing multicentre interventional single‐arm trial for CP transfusion in critically ill COVID‐19 patients | Franchini et al. 2020 [[79](#_ENREF_79)] |
|  |  |  | Key points in an operational protocol for donation of anti‐COVID‐19 CP in Italy | Franchini et al. 2020 [[80](#_ENREF_80)] |
|  |  |  | Summary of six observational studies (a total of 33 patients), requirements for CP collection (e.g., titers>1:320), storage  (e.g., 1°C and 6°C for up to 40 days) and transfusion (e.g., ABO compatibility), recommendation of CP therapy for patients severely ill with COVID-19 upon hospitalisation | Islam et al. 2020 [[81](#_ENREF_81)] |
|  |  |  | Recommendations for therapeutic plasma exchange with CP to be performed earlier and in patients with severe COVID-19 | Kesici et al. 2020 [[82](#_ENREF_82)] |
|  |  |  | A brief summary of initial experience establishing a CP program involving donor recruitment, CP testing and transfusion (dose and ABO compatibility), assessment of the effectiveness of CP therapy | Knudson and Jackson 2020 [[83](#_ENREF_83)] |
|  |  |  | Summary of previous studies and existing/ undergoing clinical trials of CP therapy in support of its emergency use for COVID-19 | Kumar et al. 2020 [[84](#_ENREF_84)] |
|  |  |  | History and current evidence supporting the use of CP for COVID-19 treatment based on the results from a number of clinical studies | McAllister et al. 2020 [[85](#_ENREF_85)] |
|  |  |  | Historical use of CP for infectious diseases, current and future perspectives of CP therapy for COVID-19 | Montelongo-Jauregui et al. 2020 [[86](#_ENREF_86)] |
|  |  |  | Opinions of CP and hyperimmune globulin therapy for COVID-19: potential benefits and risks | Morabito and Gangadharan 2020 [[87](#_ENREF_87)] |
|  |  |  | Comments on the review by Valk et al. 2020 and implications for the use of CP in South Africa | Nnaji et al. 2020 |
|  |  |  | Perspectives of the National Institutes of Health on COVID-19 treatment with CP based on scientific rationale, historical precedents and current scientific evidence | Pau et al. 2020 [[88](#_ENREF_88)] |
|  |  |  | Discussion about CP donor availability and use of serosurveys to identify CP donors and targeted populations at high risk of exposure to COVID-19 | Perez-Cameo and Marin-Lahoz 2020 [[89](#_ENREF_89)] |
|  |  |  | Metadata analysis of the efficacy of CP treatment based on 9 clinical studies suggesting that CP reduced viral loads, C-reactive protein levels and improved the clinical status of COVID-19 patients, when compared to baseline | Rabelo-da-Ponte et al. 2020 [[90](#_ENREF_90)] |
|  |  |  | Comments on the limitations of a clinical study by Shen et al. and recommendations for collection and use of CP and future clinical investigations of its therapeutic efficacy | Roback and Guarner 2020 [[91](#_ENREF_91)] |
|  |  |  | Existing evidence, design of clinical studies, assessment of the titre of antiviral antibodies using a series of recently‐developed assays, two ongoing RCTs | Roberts et al. 2020 [[92](#_ENREF_92)] |
|  |  |  | Perspectives of current and past clinical studies of CP therapy, supply and demand, timing and dosing, and future immunotherapy for COVID-19 | Rubin 2020 [[93](#_ENREF_93)] |
|  |  |  | Recommendations based on current evidence for the compassionate use of CP in patients with severe COVID-19 in developing countries with adaptations to their conditions and a thorough risk-benefit evaluation for each patient, and more research in the field. | Sabando Velez et al. 2020 [[94](#_ENREF_94)] |
|  |  |  | Past experience with CP therapy during previous SARS and Ebola outbreaks, current evidence and ongoing trials for CP therapy for COVID-19, recommendations for neutralizing antibody titers, treatment population, and low risks, more use of CP for the treatment of severely ill patients and earlier use in the course of illness and/or for prophylaxis | Sahu et al. 2020 [[95](#_ENREF_95)] |
|  |  |  | Suggested CP use as a stopgap option amidst pandemic while the efforts by authorities were needed to protect high risk individuals and consider its urgent preparation and the emergent use | Sheikh and Baig 2020 [[96](#_ENREF_96)] |
|  |  |  | World-wide efforts on deploy CP and hyperimmune globulin for COVID-19 treatment | Sheridan 2020 [[97](#_ENREF_97)] |
|  |  |  | Recommendations of localized herd immunity and CP to impede the spread of and fight against COVID‐19 | Syal 2020 [[98](#_ENREF_98)] |
|  |  |  | Brief note of CP therapy for COVID-19 and other coronavirus infections, and a possible trial in India | Teixeira da Silva 2020 [[99](#_ENREF_99)] |
|  |  |  | Positive results of CP therapy for other virus infections and COVID-19, upcoming clinical trials for different populations, requirement of CP donation | The Lancet Haemato-logy 2020 [[100](#_ENREF_100)] |
|  |  |  | No impairment of psoralen and ultraviolet light pathogen inactivation on the stability and neutralising capacity of SARS-CoV-2-specific antibodies in CP | Tonn et al. 2020 [[101](#_ENREF_101)] |
|  |  |  | Positive views of the role of CP in infectious diseases in particular the collection, production and usage of CP in Hong Kong | Wong and Lee 2020 [[102](#_ENREF_102)] |
|  |  |  | Combination of CP therapy with other treatment mechanisms, precautions for its side effects, requirements for institutional support | Yoo 2020 [[103](#_ENREF_103)] |
|  |  |  | Several issues raised: optimal timing of administering CP, availability of CP with SARS-CoV-2 neutralizing antibody titer ≥1:160, adverse reactions related to CP transfusion while recommending CP therapy as an alternative option in emergent situation of COVID-19 | Zhao and He 2020 [[104](#_ENREF_104)] |
|  |  |  | Highlights of some experiences in CP collection and infusion to treat COVID-19 patients in China | Zhu et al. 2020 [[105](#_ENREF_105)] |
|  |  | Neutral | Both PROs and CONs, a tried and tested approach to a short-term solution | Adriana et al. 2020 [[106](#_ENREF_106)] |
|  |  |  | Viewpoint of using a combination of monoclonal antibodies derived from convalescent human B cell hybridomas against multiple immunogenic targets of SARS-CoV-2 spike protein | Begum and Ray 2020 [[107](#_ENREF_107)] |
|  |  |  | Comments on the paper by Hegerova et al. and other clinical studies on the use of CP to treat COVID-19 in terms of their limitations due to concomitant therapies, various dosage and tittering, majority of patients with severe or life-threatening COVID-19 | Bloch 2020 [[108](#_ENREF_108)] |
|  |  |  | Response letter regarding the comments on the safety and efficacy of CP therapy for COVID-19 based on the information available at the time of submission, highlighting the need for RCTs | Brown 2020 [[109](#_ENREF_109)] |
|  |  |  | Implications of short duration of neutralizing antibody titers for immunity and ongoing efforts to deploy CP for prevention and therapy of COVID-19 | Casadevall et al. 2020 [[110](#_ENREF_110)] |
|  |  |  | Implications of the kinetics of viral load and the antibody responses of 23 hospitalized patients with mild and severe COVID-19 for the use of CP therapy | Casadevall et al. 2020 [[111](#_ENREF_111)] |
|  |  |  | Potential risks and ethical considerations as an immunologically based strategy, a tried and tested approach and perhaps helpful in the short term | Cunningham et al. 2020 [[112](#_ENREF_112)] |
|  |  |  | Risk–benefit analysis based on theoretical reasons and limited data available on the safety and efficacy of CP therapy for COVID-19 | Dhanasekaran et al. 2020 [[113](#_ENREF_113)] |
|  |  |  | Potential harm by CP to patients and overall worldwide health care response to COVID-19, suggesting an urgent need for high-quality randomized trials | Dzik 2020 [[114](#_ENREF_114)] |
|  |  |  | Criticism of US FDA’s authorisation for the emergency use of CP for COVID-19 and urgency for high quality evidence from large RCTs | Estcourt and Roberts 2020 [[115](#_ENREF_115)] |
|  |  |  | Recommendations on the development of hyperimmune immunoglobulin from CP without compromising the supply of CP for COVID-19 through the establishment of global networks and harmonisation between the major regulatory agencies | Farrugia 2020 [[116](#_ENREF_116)] |
|  |  |  | Current clinical studies may underestimate risk of antibody-dependent enhancement due to lack of representation of patients in the early phase of infection and confounding from multiple concurrent therapies and small patient numbers | Fleming and Raabe 2020 [[117](#_ENREF_117)] |
|  |  |  | Preference of blood group O donors for CP in COVID-19 for additional benefit over anti-SARS-CoV 2 neutralizing antibodies due to high anti-A isoagglutinin titer | Focosi 2020 [[118](#_ENREF_118)] |
|  |  |  | Built on the literature, questions raised regarding neutralizing antibodies, donors, testing and qualification of CP, timeframe for transfusing CP to recipients, quality of evidence and ethics of clinical trials while considering CP therapy as a rescue treatment in the absence of obvious form of treatment and soon-coming vaccine | Garraud 2020 [[119](#_ENREF_119)] |
|  |  |  | Issues on CP donor recruitment: availability, antibody tests, CP collection and use | Gniadek and Donnersberger 2020 [[120](#_ENREF_120)] |
|  |  |  | Comments on seven reported clinical studies of the efficacy of CP therapy for COVID-19, with respect to the anti-SARS-CoV-2 antibody level and disease severity of patients before the treatment, and the lack of a control group, and recommendation for a RCT | Han and Zhou 2020 [[121](#_ENREF_121)] |
|  |  |  | Comments on some of the clinical trials for treatment of COVID-19 with CP and recommendations for its use for the patients with an early infection and likely to progress to more severe illness | Katz [[122](#_ENREF_122)] |
|  |  |  | Built on literature of CP therapy for other viruses and a case series of COVID-19, a number of questions proposed to be answered | Langhi et al. 2020 [[123](#_ENREF_123)] |
|  |  |  | Potential sources for obtaining safer therapeutic plasma or autologous antibodies to treat COVID-19 | Lanza and Seghatchian 2020 [[124](#_ENREF_124)] |
|  |  |  | Comments on the US FDA approval for emergency use of CP and current evidence for its efficacy to treat COVID-19 | Mahase 2020 [[125](#_ENREF_125)] |
|  |  |  | Misleading claim of 35% reduced deaths by CP and insufficient evidence for any efficacy of CP therapy for COVID-19 | Mahase 2020 [[126](#_ENREF_126)] |
|  |  |  | Introduction to CP, its benefits and risks, how to donate | Malani et al. 2020 [[127](#_ENREF_127)] |
|  |  |  | Criticism on the review article of Brown and McCullough, regarding the inconsistent information in the text and Table for three cited clinical studies, cautions about the efficacy and safety of CP | Pawitan 2020 [[128](#_ENREF_128)] |
|  |  |  | Proposed use of virus neutralizing antibody from CP in the form of an isopathic preparation for treatment of COVID-19 | Prajapati 2020 [[129](#_ENREF_129)] |
|  |  |  | Historical use of CP therapy for other infectious diseases and limited clinical trials for COVID-19, several problems to be addressed | Saverino 2020 [[130](#_ENREF_130)] |
|  |  |  | Involvement of Antimicrobial Stewardship Programs in CP pre-authorization process to enhance the optimal use of CP | Stevens et al. 2020 [[131](#_ENREF_131)] |
|  |  |  | Selection of CP donors for COVID-19 with the highest levels of detectable neutralising antibody | Tedder and Semple 2020 [[132](#_ENREF_132)] |
|  |  |  | Evidence to support and cautions against CP therapy for COVID-19 based on past and current clinical studies, challenges around the collection of CP, and theoretical risks of CP transfusion | van den Berg et al. 2020 [[133](#_ENREF_133)] |
|  |  |  | Commentary on the influence of CP characteristics in particular levels of SARS-CoV-2 specific IgG antibodies on CP-associated outcomes | Verkerke et al. 2020 [[134](#_ENREF_134)] |
|  |  |  | Historical and current experiences with CP therapy for infectious diseases including COVID-19, mechanism of action of CP, challenges for clinical studies of safety and efficacy of CP therapy | Xi 2020 [[135](#_ENREF_135)] |
|  |  |  | Some questions raised for two clinical studies by Duan et al. and Shen et al. in terms of requirement for virus inactivation, optimal time to collect CP from a donor, transfusion volume and neutralizing antibody titer, previous severity of the CP donors | Zeng et al. 2020 [[136](#_ENREF_136)] |
|  |  |  | Built on the literature, questions raised regarding neutralizing antibodies, donors, testing and qualification of CP, timeframe for transfusing CP to recipients, quality of evidence and ethics of clinical trials while considering CP therapy as a rescue treatment in the absence of obvious form of treatment and soon-coming vaccine | Garraud 2020 [[119](#_ENREF_119)] |
|  |  |  | Potential of equine polyclonal antibodies as a sound alternative to CP for COVID-19 | Zylberman et al. 2020 [[137](#_ENREF_137)] |
|  |  | Negative | Potential treatment of severe COVID-19 with HLA‐E‐restricted unconventional CD8 T cells superior to SARS-CoV-2 specific and HLA‐matched cytotoxic T cells, both could be rapidly and cost‐effectively prepared in large numbers from convalescent COVID-19 patients | Caccamo 2020 [[138](#_ENREF_138)] |
|  |  |  | Concerns about the adverse effect and blood-borne pathogen transmission from CP therapy low- and middle-income nations because of their lack of healthcare infrastructure and regulations for collecting and administering blood products | Ferreira and Mostajo-Radji 2020 [[139](#_ENREF_139)] |
|  |  |  | Risk of contamination and hypersensitivity to proteins in CP and exaggerated benefits | Joob and Wiwanitkit 2020 [[140](#_ENREF_140)] |
|  |  |  | Safety issues associated with CP use, in particular thrombotic events with high risk of pulmonary embolism, and longer timeframe (>4 h) to evaluate the incidence of adverse events | Sanfilippo et al. 2020 [[141](#_ENREF_141)] |
|  |  |  | Cautiousness with CP for COVID-19 treatment due to potential pro-coagulant effects worsening underlying hypercoagulability and perfusion in vital organs | Sanfilippo et al. 2020 [[142](#_ENREF_142)] |
|  |  |  | Pathogen contamination and no protective immunity in CP, and other classic therapeutic options with likely lower risk for COVID-19 such as hydroxychloroquine | Wiwanitkit 2020 [[143](#_ENREF_143)] |
| Review | 46 | Rapid review | Use of CP for SARS, MERS, EBOV, H1N1, COVID-19 and summary of ongoing clinical trials | Barone and DeSimone 2020 [[144](#_ENREF_144)] |
|  |  |  | Introduction and challenges of CP therapy, and summary of ongoing clinical trials of CP therapy for COVID-19 | Majbour and El-Agnaf 2020 [[145](#_ENREF_145)] |
|  |  | State-of-the-art review | Clinical use of convalescent plasma for infectious diseases including 3 case series for COVID-19 (total 19 patients) and pathogen reduction of CP recommendations for establishing a convalescent plasma program, enhancement considerations for convalescent plasma, and considerations around pathogen reduction treatment of convalescent plasma | Brown and McCullough 2020 [[146](#_ENREF_146)] |
|  |  |  | Historical use of CP and current approaches for donor selection and CP collection, pooling technologies, pathogen inactivation systems, and banking of CP, results of published clinical studies of CP therapy, and list of ongoing registered clinical trials. | Focosi et al. 2020 [[147](#_ENREF_147)] |
|  |  | Scoping review | Clinical trials of CP for SARS, Influenza A/B, Ebola virus, COVID-19 and timing of CP treatment | Cao and Shi 2020 [[148](#_ENREF_148)] |
|  |  |  | Assessment of the feasibility to conduct a rapid and timely meta-analysis based on the review of the registered clinical trials of CP for COVID-19 identified on [ClinicalTrials.gov](http://clinicaltrials.gov/) and the WHO registry of COVID-19 studies ([www.who.int/ictrp/en/](http://www.who.int/ictrp/en/)) | Zheng et al. 2020 [[149](#_ENREF_149)] |
|  |  | Review of the evidence | Examination of the risk of antibody-dependent enhancement from CP-derived polyclonal hyperimmune globulin therapy and its mitigation based on literature search in English on PubMed from 1965 till March 2020 | de Alwis et al. 2020 [[150](#_ENREF_150)] |
|  |  |  | Existing clinical studies of passive immunization in particular CP therapy for COVID-19 and SARS-CoV-2 immunology | Fischer et al. 2020 [[151](#_ENREF_151)] |
|  |  |  | The mechanism of action and potential side effects of CP, evidence supporting its use from previous infectious diseases epidemic and current COVID-19 pandemic, and donor selection criteria | Mucha and Quraishy 2020 [[152](#_ENREF_152)] |
|  |  | Systematic review  and meta-analysis | An update on the review by Piechotta et al. 2020 [[153](#_ENREF_153)] with more completed and ongoing studies identified on 19 August 2020 | Chai et al. 2020 [[154](#_ENREF_154)] |
|  |  |  | Safety and efficacy of CP therapy for other severe respiratory viral infections to provide indirect evidence for CP therapy for COVID-19 | Devasenapathy et al. 2020 [[155](#_ENREF_155)] |
|  |  |  | Systematic search in WHO COVID‐19 Global Research Database, MEDLINE, Embase, Cochrane COVID‐19 Study Register, CDC COVID‐19 Research Article Database and trial registries on 4 June 2020 to identify completed and ongoing studies of the safety and efficacy of CP or hyperimmune immunoglobulin transfusion in the treatment of COVID-19 | Piechotta et al. 2020 [[153](#_ENREF_153)] |
|  |  |  | Systematic search in major electronic databases (PubMed, Medline, Google Scholar and MedRxiv) to identify available evidence on the CP for treatment of COVID-19 up to 10 July 2020 in accordance with the PRISMA guideline | Sarkar et al. 2020 [[156](#_ENREF_156)] |
|  |  |  | Systematic search in major electronic databases (PubMed, Web of  Science, Embase, and the Cochrane Library) to identify available evidence on the CP for treatment of different types of infectious including COVID-19 up to March 30, 2020 in accordance with the PRISMA guideline | Sun et al. 2020 [[157](#_ENREF_157)] |
|  |  | Overview | Pathophysiology of COVID-19 and summary of three clinical studies of CP treatment and discussion of low-dose radiation therapy for COVID-19 and combination therapy involving both | Abdollahi et al. 2020 [[158](#_ENREF_158)] |
|  |  |  | Passive immunotherapy, requirements for hyperimmune plasma donor and product, directions and methods of clinical use | Annamaria et al. 2020 [[159](#_ENREF_159)] |
|  |  |  | Explanation for how and why the CP can serve as a plausible therapeutic modality and current clinical trials for its use for COVID-19 treatment. | Anudeep et al.2020 [[160](#_ENREF_160)] |
|  |  |  | Evidence of benefit, regulatory considerations, logistical workflow (donor eligibility, donor recruitment, collections and  transfusion), and proposed clinical trials | Bloch et al. 2020 [[161](#_ENREF_161)] |
|  |  |  | Brief of CP therapy, monoclonal antibody therapy, and SARS-CoV-2 RBD mutations and infectivity | Kumar et al. 2020 [[162](#_ENREF_162)] |
|  |  |  | Perspectives of immunity (cross-reactivity, vaccination) and immune therapy (intravenous immunoglobulin, CP, monoclonal antibodies) in COVID-19 | Gasparyan et al. 2020 [[163](#_ENREF_163)] |
|  |  |  | Historical evidence for CP in previous infectious outbreaks including MERS and SARS-Cov-1 and implication for CP as an explicit option for containment of COVID-19 disease. | Iftikhar et al. 2020 [[164](#_ENREF_164)] |
|  |  |  | Mechanism of action, historical evidence for CP in previous infectious outbreaks, safety of CP therapy, and factors affecting the efficacy of CP therapy for COVID-19 | Li et al. 2020 [[165](#_ENREF_165)] |
|  |  |  | Following topics covered: the immune responses of COVID-19 patients, passive immunity therapy using immunoglobulin or CP including two clinical studies for COVID-19, potential adverse effects of CP, procedure for CP use | Lindholm et al. 2020 [[166](#_ENREF_166)] |
|  |  |  | Survey of current clinical trials of CP to treat COVID-19 infection and description of their characteristics including study design, patients population, outcomes, eligibility criteria for CP donors, CP collection, antibody titre and CP dose | Murphy et al.2020 [[167](#_ENREF_167)] |
|  |  |  | Background, immunological basis and clinical indications of CP treatment for COVID-19, some examples and future perspectives of clinical applications of CP therapy | Sayinalp et al. 2020 [[168](#_ENREF_168)] |
|  |  |  | Overview of RCTs of CP and hyperimmune intravenous globulin for treatment for severe influenza A and B and reported clinical studies on the use of CP in OCIVD-19 (two case reports, one case-control, one observational study and one RCT) and key considerations for passive immunization studies for COVID-19 | Subbarao et al. 2020 [[169](#_ENREF_169)] |
|  |  | Mixed studies review | Literature search using Elsevier, PubMed, Taylor & Francis, Springer, Nature and Google search engines, and consultation with experts, highlights of clinical studies of CP therapy for SARS–CoV-2 and other viruses infections, protocol of CP therapy for COVID-19 and any potential risks | Pawar et al. 2020 [[170](#_ENREF_170)] |
|  |  | Systematic review | Systematic search in electronic databases (PubMed, Embase, Google Scholar, Cochrane Library, and Medline) to identify clinical studies related to CP and COVID-19 up to June 2020 in accordance with the PRISMA guideline for a systematic review | Bakhtawar et al. 2020 [[171](#_ENREF_171)] |
|  |  |  | Analyses of three clinical studies in China involving 19 patients who received CP transfusion and recommendations for collection, testing and clinical use of COVID-19 CP identified by searching PubMed, Embase and Medline databases from December 8, 2019 (first breakout) to May 5, 2020 | Chen and Xia 2020 [[172](#_ENREF_172)] |
|  |  |  | Systematic search in major electronic databases (PubMed, Embase, and Medline) to identify available evidence on the CP for treatment of COVID-19 up to 19 April 2020 in accordance with the PRISMA guideline | Rajendran et al. 2020 [[173](#_ENREF_173)] |
|  |  |  | Systematic search in WHO COVID-19 Global Research Database, MEDLINE, Embase, Cochrane COVID‐19 study register, Centers for Disease Control and Prevention COVID-19 research article database and trials registries to identify ongoing studies and results of completed studies on 23 April 2020 for case-series, cohort, prospectively planned, and RCTs for CP therapy | Valk et al. 2020 [[174](#_ENREF_174)] |
|  |  |  | A PubMed search was conducted on 13 July 2020 to summarize the literature and identify future research regarding CP therapy for coronaviruses (SARS-CoV, MERS-CoV and, in particular, SARS-CoV-2) as well as currently registered RCTs for CP in COVID-19 from the World Health Organization International Clinical Trials Registry and clinicaltrials.gov | Wooding and Bach 2020 [[175](#_ENREF_175)] |
|  |  | Critical review | Summary of the results from RCTs published to date and analysis their flaws and biases to provide suggestions for next round of RCTs, with regard to CP specification, therapeutic dose, timing, control arm, disease stage, and outcome measures | Daniele and Albert 2020 [[176](#_ENREF_176)] |
|  |  |  | Various aspects and limitations of CP therapy for COVID-19 based on current literature | Nagoba et al. 2020 [[177](#_ENREF_177)] |
|  |  |  | Available evidence about CP in COVID-19 and other epidemics, mechanisms of action, registered trials on CP therapy for COVID-19, guidance from authorities for donor selection including neutralizing antibody titers, CP recipients and blood establishments. | Psaltopoulou et al. 2020 [[178](#_ENREF_178)] |
|  |  |  | CP to treat virus diseases and SARS‐CoV‐infected patients without significant adverse events, antibody‐mediated immunopathology and response in coronavirus diseases, CP collection and study design to evaluate the safety and efficacy of COVID‐19 CP | Tiberghien et al. 2020 [[179](#_ENREF_179)] |
|  |  | Literature review | Historical precedents and recent clinical studies of CP for infectious diseases and COVID-19, mechanism of action, safety, patient and donor eligibility, antibody titer measurement, and system development for CP therapy | Choi 2020 [[180](#_ENREF_180)] |
|  |  |  | Introduction of SARS-CoV-2, brief of diagnosis and treatment of COVID-19,  potential benefits and risks, mechanism of CP therapy, summary of eight completed clinical studies and challenges of CP therapy in patients with COVID-19 | Khulood et al. 2020 [[181](#_ENREF_181)] |
|  |  |  | Review of the clinical utility of convalescent blood products, primarily CP and immunoglobulins, and available evidence in the previous coronavirus epidemics and COVID-19 pandemic | Long et al. 2020 [[182](#_ENREF_182)] |
|  |  |  | Review of the mechanism, completed and ongoing clinical studies, challenges and future direction for CP therapy in the treatment of COVID-19 | Ouyang et al. 2020 [[183](#_ENREF_183)] |
|  |  |  | Concept of passive antibody therapy including CP therapy, strategy, mechanisms, risks, current evidence and ongoing clinical trials of CP therapy for COVID-19 | Piyush et al. 2020 [[184](#_ENREF_184)] |
|  |  |  | Clinical studies of CP in patients with respiratory infection by coronavirus (SARS, MERS, and SARS-CoV-2), associated adverse events, acquisition and plasma composition, antiviral mechanisms, and immunomodulation | Rojas et al. 2020 [[185](#_ENREF_185)] |
|  |  |  | Basic principles of passive immunization, with particular reference to CP, CP therapy during past epidemics and current pandemics, analysis of the possible side effects, summary of clinical studies and various aspects regarding CP therapy for COVID-19 | Selvi 2020 [[186](#_ENREF_186)] |
|  |  |  | Current status of various antibody-based immunotherapeutics such as CP, monoclonal and neutralizing antibodies, and intravenous immunoglobulins against COVID-19 with highlights of their advantages, disadvantages, and clinical utility | Sharun et al. 2020 [[187](#_ENREF_187)] |
|  |  |  | Clinical studies of CP used during previous viral outbreaks and pandemics, and potential use of CP during the present COVID-19 pandemic (three case series) with a risk benefit analysis, pros and cons of CP against COVID-19 | Sullivan and Roback 2020 [[188](#_ENREF_188)] |
|  |  |  | Historical examples and recent clinical studies of CP for infectious diseases and COVID-19, mechanism of action, regulations of CP collection, CP dose, patient selection, CP for prophylaxis, risks of CP administration | Yigenoglu et al. 2020 [[189](#_ENREF_189)] |
| Protocol/  Guidance | 19 | Preparation/production of CP | Requirements for CP donors, and the standards for preparation, qualification, storage, distribution and control of use of the product | Accorsi et al. 2020 [[190](#_ENREF_190)] |
|  |  | A study protocol for a non-randomized trial | A national collaborative multicenter phase II cohort study in Saudi Arabia for assessing feasibility, safety, and potential efficacy of CP in treating COVID-19 patients with severe disease in comparison with a propensity score matched control | Albalawi et al. 2020 [[191](#_ENREF_191)] |
|  |  | Clinical study and application of CP | Gaps in knowledge were identified as follows: study design, patient eligibility, dose, frequency and timing of administration, parameters to assess response to the treatment and long‐term outcome, adverse events, and CP application in less resourced countries as well as in paediatrics and neonates | Al-Riyami et al. 2020 [[192](#_ENREF_192)] |
|  |  | Conceptual framework | An intelligence-integrated concept to identify the most appropriate CP for corresponding prioritised patients with COVID-19 to help doctors hasten treatments | Albahri 2020 [[193](#_ENREF_193)] |
|  |  | expert opinion, survey of group members  and review of available evidence  expert opinion, survey of group members  and review of available evidence  Expert opinion, survey of group members and review of available evidence | Donor selection, recruitment, blood collections, processing and distribution pre-donation qualification of CCP donors (including antibody testing) and operational considerations pertaining to collection, storage and distribution of CCP | Bloch et al. 2020 [[194](#_ENREF_194)] |
|  |  | COVID‐19 CP program | Registration of hospitals and investigators with a national IND protocol, collaboration with a regional blood donor center, targeted recruitment of CP, IT support for CP ordering, distribution, and transfusion, prioritization of patients to receive CP, and evaluation of CP products including antibody characteristics and patient response to therapy | Blackall et al. 2020 [[195](#_ENREF_195)] |
|  |  |  | Donor identification and recruitment, quantification, scheduling, CP collection by apheresis, testing, processing, labeling, inventory management and distribution | Budhai et al. 2020 [[196](#_ENREF_196)] |
|  |  | Study protocol for RCTs | A structured summary of a study protocol of a phase II RCT to assess the safety, efficacy and dose response of CP transfusion in severe COVID-19 patients | Chowdhury et al. 2020 [[197](#_ENREF_197)] |
|  |  |  | A prospective, single-center, phase 2, RCT to evaluate the efficacy and safety of CP in hospitalized adults with severe SARS-CoV-2 infection | Eckhardt et al. 2020 [[198](#_ENREF_198)] |
|  |  |  | An open label phase II RCT with or without infusion of CP to assess the clinical outcome in high-risk patients with confirmed severe COVID-19 | Janssen et al. 2020 [[199](#_ENREF_199)] |
|  |  | Perspective document of the Working Party on Global Blood Safety of the International Society of Blood Transfusion | Key factors for eligibility criteria of CP donors; pre‐screening and pre‐donation testing of CP donors; criteria for CP collection; post‐donation treatment of CP; and recommendations for CP transfusion | Epstein and Burnouf 2020 [[200](#_ENREF_200)] |
|  |  | Commentary | The same quality and safety standards for collection and use of CP in low- and middle‐income countries as in the high-income countries | Epstein et al. 2020 [[201](#_ENREF_201)] |
|  |  | Guidance for treating early to moderate COVID-19 patients with CP | The guidance includes patient and donor selection criteria, plasma harvesting, plasma product specifications, dosage and precautions for CP collection and transfusion process and is to be adopted by Sudan's health authority | Hassan et al. 2020 [[202](#_ENREF_202)] |
|  |  | Initiative for provision of CP | Collaboration and resource management for CP collection, inventory development, clinical use and donor recruitment in Arkansas with constrained resources | Ipe et al. 2020 [[203](#_ENREF_203)] |
|  |  | A pilot program of CP collection | Criteria for CP donor screening and selection, collection procedures via plasmapheresis, CP testing for SARS-CoV-2 nucleic acid and S‐RBD-specific IgG antibody | Li et al. 2020 [[204](#_ENREF_204)] |
|  |  | Strategy and experience | Criteria for CP donor recruitment, collection and preparation of CP, laboratory examination of CP, guidance for clinical use, and three clinical cases of COVID-19 treatment with CP | Pei et al. 2020 [[205](#_ENREF_205)] |
|  |  | An one arm proof-of-concept clinical trial protocol | Efficacy of the administration of CP therapy for critically ill patients with COVID-19 in terms of their survival | Perotti et al. 2020 [[206](#_ENREF_206)] |
|  |  | An apheresis research project proposal | Suggestions for CP collection process, donor safety, clinical use, quality management, targeted patients | Seghatchianaand Lanza 2020 [[207](#_ENREF_207)] |
|  |  | Authority guide by Turkish Ministry of Health | Principles and criteria for collection, preparation and clinical use of CP, and a treatment follow-up process | Yilmaz et al. 2020 [[208](#_ENREF_208)] |
| In vitro testing of convalescent plasma | 35 | ELISA with recombinant antigen (e.g., spike protein sequences) as substrates | An ELISA that could detect different antibody types in serum and plasma, and correlate with neutralizing activity to identify highly reactive human donors for CP therapy | Amanat et al. 2020 [[209](#_ENREF_209)] |
|  |  |  | A high-throughput competitive assay that could simultaneously determine an individual's seropositivity against the SARS-CoV-2 Spike protein and estimate the neutralizing capacity of anti-Spike antibodies to block interaction with the human ACE2 required for viral entry, and be used to identify candidate sera for therapeutic use | Byrnes 2020 [[210](#_ENREF_210)] |
|  |  | ELISA plate-bound recombinant ACE2 and SARS-CoV-2 RBD | or antibodies and compounds  capable of inhibiting the binding  Plasma from COVID-19 convalescent patients approximately 10 weeks after confirmation of COVID-19 by RT-PCR with relatively mild symptoms and no need for hospitalization contained significant amount of SARS-CoV-2-specific IgG, but did not always inhibit the virus receptor binding | Gattinger et al. 2020 [[211](#_ENREF_211)] |
|  |  | ELISA based on SARS-CoV-2 RBD and NP | Anti-SARS-CoV-2 RBD antibodies (IgM, G and A) responses were significantly correlated to the disease severity in a cohort of 350 convalescent patients with previous COVID-19 infection, ranging from asymptomatic to critical cases | [[212](#_ENREF_212)] |
|  |  | Commercial LFA and ELISA targeting SARS-CoV-2 antigens | A combination of antigenic targets (NP, spike protein, S-RBD) may be required to improve the accuracy of IgG detection in CP donors | DomBourian et al. 2020 [[213](#_ENREF_213)] |
|  |  | Pseudovirus capture assay and virus neutralization (VN) assay | The capacity of CP to bind to SARS-CoV-2 spike protein correlated with neutralizing activity against recombinant viruses (Spearman correlation coefficients ρ >0.86), but did not always translate into neutralization | Ding et al. 2020 [[214](#_ENREF_214)] |
|  |  | VN assay using SARS-CoV-2 strain and monkey Vero-E6 cells | In vitro evaluation of potency of CP and potential anti-SARS-CoV-2 drug candidates for COVID-19 treatment | Ianevski et al. 2020 [[215](#_ENREF_215)] |
|  |  | VN assays based on different viruses | The neutralizing activity of CP and human monoclonal antibodies measured using pseudotyped and chimeric viruses correlated quantitatively with that measured using an authentic SARS-CoV-2 neutralization assay (ρ≥0.86) | Schmidt et al. 2020 [[216](#_ENREF_216)] |
|  |  | A modified cytopathogenic assay based on cell culture of Vero cells in the presence of plasma samples and SARS-CoV-2 | There were individual differences in the antibody level (neutralizing antibody titers <1:16 to >1:1024) and its changes over 12 to 60 days since onset of symptoms among 8 representative convalescent patients | Wang et al. 2020 [[217](#_ENREF_217)] |
|  |  | VN assay using mNeonGreen SARS-CoV-2 and Vero-E6 cells | A high-throughput fluorescence-based assay comparable to plaque reduction neutralizing assay, the gold standard of test for SARS-CoV-2 neutralizing activity, useful to identify donors with high-titers for CP for COVID-19 therapy | Muruato et al. 2020 [[218](#_ENREF_218)] |
|  |  | A lateral flow assay (LFA) testing platform | CP collected from adults who met all criteria for donating blood, had confirmed COVID19 by positive SARS-CoV-2 PCR test, had complete resolution of symptoms at least 14 days prior to donation, showed qualitatively diverse (strong, weak and negative) IgG and IgM profiles, with 87.3% and 50.8% being positive for IgG and IgM, respectively | Ragnesola et al. 2020 [[219](#_ENREF_219)] |
|  |  | A fluorescence immunoassay (CEFIA) and a microsphere immunoassay (MIA) | CP showed a wide range of antibody levels.  Agreement between the two immunoassays was 90.4%-94.5% | Yang et al. 2020 [[220](#_ENREF_220)] |
|  |  | Microarray | A microarray containing a panel of antigens from SARS-CoV-2 spike protein and nucleoprotein in addition to other human coronaviruses | de Assis et al. 2020 [[221](#_ENREF_221)] |
|  |  | Commercial CLIA for anti-SARS-CoV-2 IgM and IgG, and PCR test | 82% CP donors had positive IgG antibodies, 40% donors tested positive by PCR even symptom-free for ≥2 weeks. There was a decline in the IgG level over a short duration of 10 days. CP recipients with detectable plasma viral load had lower IgG levels; there was no relationship between plasma viral load, blood type or death. | Dulipsingh et al. 2020 [[222](#_ENREF_222)] |
|  |  | Commercial immunoassays based on SARS-CoV-2 SP and NP antigens | Anti-SARS-CoV-2 spike protein IgG antibody strength correlated with age and hospitalization for COVID-19, suggesting individuals who suffered severe COVID-19 disease symptoms may represent better CP donors. Total anti-SARS-CoV-2 nucleocapsid protein antibody strength correlated with time from symptom resolution to sample collection and symptom duration. | Ikegami et al. 2020 [[223](#_ENREF_223)] |
|  |  | Commercial CLIA for detecting RBD-specific  IgG, IgM and IgA levels | RBD-specific serum IgG, IgM and IgA COVID-19 convalescent patients continued to decline from 28 to 99 days after hospital discharge | Ma et al. 2020 [[224](#_ENREF_224)] |
|  |  | PCR based tests | SARS-CoV-2 neutralizing antibodies were detectable as early as 10 days after onset of symptoms and continue to rise, plateauing after 18 days and were not altered by amotosalen and UVA radiation to inactivate potentially contaminating infectious pathogens in CP | Danh et al. 2020 [[225](#_ENREF_225)] |
|  |  |  | Detectable viral RNA in older COVID-19 patients screened for CP donation even 12 to 24 days after symptom resolution | Hartman et al. 2020 [[226](#_ENREF_226)] |
|  |  | VN assays based on pseudotyped and live SARS-CoV-2 virus, and ELISA based on S-RBD and ACE2 | ELISA results had an overall agreement with authentic VN titers with a coefficient of determination of 0.6) and a high correlation with pseudotyped VN titers (*R^2^* = 0.76) in a cohort of 58 potential donors for CP therapy | Abe et al. 2020 [[227](#_ENREF_227)] |
|  |  | VN assays based on pseudotyped SARS-CoV-2 virus, and S-RBD-specific IgG, IgM, and IgA ELISA | The levels of S-RBD-specific IgG and IgA slightly decreased between 6 and 10 weeks after the onset of COVID-19 symptoms in contrast with a rapid decreased level of S-RBD-specific IgM. Similarly, the neutralization capacity of CP was significantly decreased a few weeks after the symptom onset. The loss of neutralizing activity over time correlated with the loss of anti-RBD IgM, IgA, and IgG antibodies, higher for IgM than for IgG and IgA | Beaudoin-Bussières et al. 2020 [[228](#_ENREF_228)] |
|  |  | VN assay and commercial anti-SARS-CoV-2 IgG ELISA | In 130 CP donors, higher levels of anti-spike avidity were associated with older age, male sex, and hospitalization. Neutralizing antibody titers correlated with anti-spike and anti-NP IgG avidity, respectively (ρ=0.386 and 0.211) | Benner et al. 2020 [[229](#_ENREF_229)] |
|  |  | VN assay using authentic SARS-CoV-2 and commercial anti-SARS-CoV-2 IgG ELISA | Amongst 250 donors studied a median of 67 days since symptom onset, 97% were seropositive on one or more assays. Sixty percent of donors had neutralizing antibody titers ≥1:80. Higher neutralizing antibody titer correlated with older age, male sex, fever during acute illness, and disease severity represented by hospitalization. The antibody titer declined in 37 of 41 paired specimens collected a median of 98 days (range, 77-120) apart. ELISA results corresponded well with the antibody titers | Boonyaratanakornkit et al. 2020 [[230](#_ENREF_230)] |
|  |  | VN assay using a SARS-CoV-2 strain and commercial ELISA and CLIA using virus antigens | There were positive correlations of varying strength (ρ= 0.37‐0.52) between antigen binding and VN assays in a cohort of 47 CP donors with a history of nonsevere COVID-19. The neutralization activity was the highest in the donors age 48-75 years compared to the younger age groups 19-37 and 38-47, but was not affected by sex fever and symptom duration | Gniadek et al. 2020 [[231](#_ENREF_231)] |
|  |  | VN assay and commercial ELISA and CLIA | There were significant positive correlations between VN assay values and ELISA or CLIA values (ρ=0.81-0.40). Some commercial assays may be useful to identify CP donors with high neutralizing antibodies | Patel et al. 2020 [[232](#_ENREF_232)] |
|  |  | SARS-CoV-2 infected cell lysate and spike protein ELISA and VN assay | In the study sample of 436 donations, more individuals with previously laboratory-diagnosed SARS-CoV-2 infection developed measurable antibody responses and neutralising antibodies than those with a self-diagnosed infection. Neutralising antibody levels declined within the first 3 months following diagnosis, which suggests the collection of CP with high neutralising antibody may be optimum within a short time window. Finally, commercial ELISA can perform effectively as surrogate assays for predicting neutralising antibody titres | Harvala et al. 2020 [[233](#_ENREF_233)] |
|  |  | Anti–NP SARS-CoV-2 IgM, IgG, and IgA ELISA and cytopathic effect–based VN test | Neutralizing antibody titers of ≥160 were found in 63.6% of 271 eligible CP donors recovered from mild/moderate COVID-19 (absence of symptoms for ≥14 days). Correlation between IgG signal/cut-off of ≥5.0 and neutralizing antibody of ≥160 was 82.4%. The neutralizing antibody titer was associated with donor's weight, days between disease onset and plasma collection, and IgG/IgM levels | Wendel et al. 2020 [[234](#_ENREF_234)] |
|  |  | VN assays based on pseudotyped and authentic SARS-CoV-2 strains, and anti-SARS-CoV-2 NP IgG ELISA | The highest neutralizing antibody titers were observed among ICU patients, followed by general hospitalized patients, and CP donors (>55% of CP samples (21/38) exhibited a titer <1:160). The pseudotype VN titer correlated with the ELISA result ((r=0.4192), but had no correlation with age | Zeng et al. 2020 [[235](#_ENREF_235)] |
|  |  | Pseudotyped VN assay, anti-SARS-CoV-2 IgG/IgM ELISA, fluorescent bead-based immunoassay | Hospitalized patients had up to 3000-fold higher antibody and neutralization titers compared to CP donors. VN titers correlated with IgG, IgM and IgA levels and increased with the subject age. There were no gender differences in the VN titer and IgG level. | Dogan et al. 2020 [[236](#_ENREF_236)] |
|  |  | Anti-SARS-CoV-2 IgG ELISA and VN assay | A range of neutralization titers from 8 to 1765 were seen in 100 CP units with a tendency of higher‐titer plasma units from donors with increased disease severity, of advanced age, and of male sex. The neutralization titer correlated with ELISA results (R^2^=0.2830) | [Jungbauer](https://onlinelibrary.wiley.com/action/doSearch?ContribAuthorStored=Jungbauer%2C+Christof) et al. 2020 [[237](#_ENREF_237)] |
|  |  | ELISA for NP-specific IgM/ IgG, and S-RBD-specific IgG, and VN assay | The S-RBD-specific IgG antibody reaches higher levels after 4 weeks from the onset of COVID-19 symptoms in potential CP donors with no symptoms for >2 weeks. N-specific IgM and IgG, and S-RBD-specific IgG levels had a negative and positive correlation with time from the onset of symptoms to the plasma donation (Pearson correlation r=−0.3591, 0.2635 and 0.4540, respectively). There is a positive correlation between the SARS-CoV-2 VN titer and the S-RBD-specific IgG titer (r=0.6222). A VN titer of 1:80 is approximately equivalent to a S-RBD-specific IgG titer of 1:1280. The antibody levels were not correlated to age, sex, or blood type. | Li et al. 2020 [[238](#_ENREF_238)] |
|  |  | Anti-SARS-CoV-2 IgG/IgM ELISA and neutralizing antibody assay | ELISA plates coated overnight with recombinant NP and S-RBD (100 ng/well) for detection of immunoglobin antibodies, pNL43Luci and GPpCAGGS co-transfected into 293T cells for the neutralizing activities of CP. There was a significant correlation between neutralizing antibody titers and AUC of anti-S-RBD IgG, but not of anti-NP IgG | Ni et al. 2020 [[239](#_ENREF_239)] |
|  |  | Anti-SARS-CoV-2 IgG/IgM ELISA and pseudotyped and authentic virus neutralization assay | Most CP obtained from individuals who recover from COVID-19 do not contain high levels of neutralizing activity with 33% undetectable | Robbiani et al. 2020 [[240](#_ENREF_240)] |
|  |  | Anti-SARS-CoV-2 IgG ELISA and virus neutralization (VN) assay | There was a strong positive correlation between both CP anti-S-RBD and anti-S- ectodomain IgG titers, and VN titer with more than 80% VN title ≥160 corresponding to IgG titer ≥1:1350 | Salazar et al. 2020 [[241](#_ENREF_241)] |
|  |  | VN assay, ELISA, CLIA, and LFA from different manufacturers | Neutralizing antibody titers of CP ranged from 1: <7.7 to 1:1765.0 with mean titer of 1:231 and a standard deviation of 331.9. The best correlations to the VN titer were obtained with the Euroimmun IgG ELISA assay (ρ=0.759) and the Wantai ELISA assay (ρ=0.729). | Weidner et al. 2020 [[242](#_ENREF_242)] |
|  |  | Biophysical antibody profiling | CP antibodies can elicit Fc-dependent functions beyond viral neutralization such as complement activation, phagocytosis and antibody-dependent cellular cytotoxicity against SARS-CoV-2 | Natarajan et al. 2020 [[243](#_ENREF_243)] |

ACE2=Angiotensin Converting Enzyme 2, ARDS=Acute Respiratory Distress Syndrome, AUC=Area under the Curve, CDC=Centers for Disease Control and Prevention, ELISA=Enzyme-Linked Immunosorbent Assay, CCP=COVID-19 Convalescent Plasma, CLIA=Chemiluminescent Immunoassay, HLA=Human Leucocyte Antigen, ICU=Intensive Care Unit, LFA=Lateral Flow Assay, MERS=Middle Eastern Respiratory Syndrome, NP=Nucleocapsid Protein, PCR=Polymerase Chain Reaction, RBD=Receptor Binding Domain, RCT=Randomized Controlled Trial, SARS=Severe Acute Respiratory Syndrome, SP=Spike Protein, S-RBD=Spike protein Receptor-Binding Domain, TRALI=Transfusion-Related Acute Lung Injury, UVA=Ultraviolet A, VN=Virus Neutralization

**References**

1. Al Helali AA, Saeed GA, Elholiby TI, Kukkady MA, Mazrouei SSA. Radiological and clinical improvement in a patient with COVID-19 pneumonia postconvalescent plasma transfusion: A case report. Radiol Case Rep 2020;15:2171-4. [doi: 10.1016/j.radcr.2020.07.031]

2. Anderson J, Schauer J, Bryant S, Graves CR. The use of convalescent plasma therapy and remdesivir in the successful management of a critically ill obstetric patient with novel coronavirus 2019 infection: A case report. Case Rep Womens Health 2020;27:e00221. [doi: 10.1016/j.crwh.2020.e00221]

3. Bao Y, Lin SY, Cheng ZH, Xia J, Sun YP, Zhao Q, et al. Clinical features of COVID-19 in a young man with massive cerebral hemorrhage—Case report. Sn Comprehensive Clinical Medicine 2020:1-7. [doi: 10.1007/s42399-020-00315-y]

4. Cinar OE, Sayinalp B, Aladag Karakulak E, Avsar Karatas A, Velet M, Inkaya AC, et al. Convalescent (immune) plasma treatment in a myelodysplastic COVID-19 patient with disseminated tuberculosis. Transfus Apher Sci 2020:102821. [doi: 10.1016/j.transci.2020.102821]

5. Clark E, Guilpain P, Filip IL, Pansu N, Le Bihan C, Cartron G, et al. Convalescent plasma for persisting COVID-19 following therapeutic lymphocyte depletion: a report of rapid recovery. Br J Haematol 2020;190:e154-e6. [doi: 10.1111/bjh.16981]

6. Figlerowicz M, Mania A, Lubarski K, Lewandowska Z, Sluzewski W, Derwich K, et al. First case of convalescent plasma transfusion in a child with COVID-19-associated severe aplastic anemia. Transfus Apher Sci 2020:102866. [doi: 10.1016/j.transci.2020.102866]

7. Grisolia G, Franchini M, Glingani C, Inglese F, Garuti M, Beccaria M, et al. Convalescent plasma for coronavirus disease 2019 in pregnancy: a case report and review. Am J Obstet Gynecol MFM 2020;2:100174. [doi: 10.1016/j.ajogmf.2020.100174]

8. Hahn M, Condori MEH, Totland A, Kristoffersen EK, Hervig TA. Pasient med alvorlig covid-19 behandlet med rekonvalesensplasma. Tidsskr Nor Laegeforen 2020;140. [doi: 10.4045/tidsskr.20.0501]

9. Hartman WR, Hess AS, Connor JP. Unusual cardiac presentation of COVID-19 and use of convalescent plasma. Case Reports in Cardiology 2020;2020:8863195. [doi: 10.1155/2020/8863195]

10. Im JH, Nahm CH, Baek JH, Kwon HY, Lee J-S. Convalescent plasma therapy in coronavirus disease 2019: a case report and suggestions to overcome obstacles. J Korean Med Sci 2020;35. [doi:

11. Jafari R, Jonaidi-Jafari N, Dehghanpoor F, Saburi A. Convalescent plasma therapy in a pregnant COVID-19 patient with a dramatic clinical and imaging response: A case report. World J Radiol 2020;12:137-41. [doi: 10.4329/wjr.v12.i7.137]

12. Jiang J, Miao Y, Zhao Y, Lu X, Zhou P, Zhou X, et al. Convalescent plasma therapy: Helpful treatment of COVID-19 in a kidney transplant recipient presenting with serve clinical manifestation and complex complications. Clin Transplant 2020:e14025. [doi: 10.1111/ctr.14025]

13. Karataş A, İnkaya AÇ, Demiroğlu H, Aksu S, Haziyev T, Çınar OE, et al. Prolonged viral shedding in a lymphoma patient with COVID-19 infection receiving convalescent plasma. Transfusion and Apheresis Science 2020;59:102871. [doi: https://doi.org/10.1016/j.transci.2020.102871]

14. Khan AM, Ajmal Z, Raval M, Tobin E. Concurrent diagnosis of acute myeloid leukemia and COVID-19: A management challenge. Cureus 2020;12:e9629. [doi: 10.7759/cureus.9629]

15. Kong Y, Cai C, Ling L, Zeng L, Wu M, Wu Y, et al. Successful treatment of a centenarian with coronavirus disease 2019 (COVID-19) using convalescent plasma. Transfus Apher Sci 2020:102820. [doi: 10.1016/j.transci.2020.102820]

16. Mira E, Yarce OA, Ortega C, Fernández S, Pascual NM, Gómez C, et al. Rapid recovery of a SARS-CoV-2 infected X-linked agammaglobulinemia patient after infusion of COVID-19 convalescent plasma. J Allergy Clin Immunol Pract 2020. [doi: 10.1016/j.jaip.2020.06.046]

17. Rodriguez Z, Shane AL, Verkerke H, Lough C, Zimmerman MG, Suthar M, et al. COVID-19 convalescent plasma clears SARS-CoV-2 refractory to remdesivir in an infant with congenital heart disease. Blood Adv 2020;4:4278-81. [doi: 10.1182/bloodadvances.2020002507]

18. Soleimani Z and Soleimani A. ADRS due to COVID-19 in midterm pregnancy: successful management with plasma transfusion and corticosteroids. J Matern Fetal Neonatal Med 2020:1-4. [doi: 10.1080/14767058.2020.1797669]

19. Xu T-m, Lin B, Chen C, Liu L-g, Xue Y. Non-optimal effectiveness of convalescent plasma transfusion and hydroxychloroquine in treating COVID-19: a case report. Virology Journal 2020;17:80. [doi: 10.1186/s12985-020-01354-6]

20. Zhang L, Pang R, Xue X, Bao J, Ye S, Dai Y, et al. Anti-SARS-CoV-2 virus antibody levels in convalescent plasma of six donors who have recovered from COVID-19. Aging (Albany NY) 2020;12:6536-42. [doi: 10.18632/aging.103102]

21. Ahn JY, Sohn Y, Lee SH, Cho Y, Hyun JH, Baek YJ, et al. Use of convalescent plasma therapy in two COVID-19 patients with acute respiratory distress syndrome in Korea. J Korean Med Sci 2020;35:e149. [doi: 10.3346/jkms.2020.35.e149]

22. Abdullah HM, Hama-Ali HH, Ahmed SN, Ali KM, Karadakhy KA, Mahmood SO, et al. Severe refractory COVID-19 patients responding to convalescent plasma; A case series. Annals of Medicine and Surgery 2020;56:125-7. [doi: https://doi.org/10.1016/j.amsu.2020.06.018]

23. Bradfute SB, Hurwitz I, Yingling AV, Ye C, Cheng Q, Noonan TP, et al. SARS-CoV-2 neutralizing antibody titers in convalescent plasma and recipients in New Mexico: An open treatment study in COVID-19 patients. J Infect Dis 2020;222:1620-8. [doi: 10.1093/infdis/jiaa505]

24. Diorio C, Anderson EM, McNerney KO, Goodwin EC, Chase JC, Bolton MJ, et al. Convalescent plasma for pediatric patients with SARS-CoV-2-associated acute respiratory distress syndrome. Pediatr Blood Cancer 2020:e28693. [doi: 10.1002/pbc.28693]

25. Enzmann MO, Erickson MP, Grindeland CJ, Lopez SMC, Hoover SE, Leedahl DD. Treatment and preliminary outcomes of 150 acute care patients with COVID-19 in a rural health system in the Dakotas. Epidemiol Infect 2020;148:e124. [doi: 10.1017/S0950268820001351]

26. Erkurt MA, Sarici A, Berber I, Kuku I, Kaya E, Ozgul M. Life-saving effect of convalescent plasma treatment in covid-19 disease: Clinical trial from eastern Anatolia. Transfus Apher Sci 2020:102867. [doi: 10.1016/j.transci.2020.102867]

27. Fung M, Nambiar A, Pandey S, Aldrich JM, Teraoka J, Freise C, et al. Treatment of immunocompromised COVID-19 patients with convalescent plasma. Transplant Infectious Disease 2020;n/a. [doi: 10.1111/tid.13477]

28. Gemİcİ A, Bİlgen H, ErdoĞan C, Kansu A, OlmuŞÇelİk O, BekÖz HS, et al. A single center cohort of 40 severe COVID-19 patients who were treated with convalescent plasma. Turk J Med Sci 2020. [doi: 10.3906/sag-2009-77]

29. Hartman W, Hess AS, Connor JP. Hospitalized COVID-19 patients treated with convalescent plasma in a mid-size city in the midwest. medRxiv 2020:2020.06.19.20135830. [doi: 10.1101/2020.06.19.20135830]

30. Ibrahim D, Dulipsingh L, Zapatka L, Eadie R, Crowell R, Williams K, et al. Factors associated with good patient outcomes following convalescent plasma in COVID-19: A prospective phase II clinical trial. Infectious Diseases and Therapy 2020. [doi: 10.1007/s40121-020-00341-2]

31. Ilona B, László G, Marienn R, Gabriella B, Lilla H, Botond L, et al. Az első két Sikeres, Convalescens Friss Fagyasztott plazmával történő terápia Hazai alkalmazása intenzív osztályon Kezelt, Kritikus állapotú, COVID-19-fertőzésben szenvedő Betegekben. Orv Hetil 2020;161:1111-21. [doi:

32. Jin H, Reed JC, Liu STH, Ho HE, Lopes JP, Ramsey NB, et al. Three patients with X-linked agammaglobulinemia hospitalized for COVID-19 improved with convalescent plasma. J Allergy Clin Immunol Pract 2020. [doi: 10.1016/j.jaip.2020.08.059]

33. Joyner MJ, Wright RS, Fairweather D, Senefeld JW, Bruno KA, Klassen SA, et al. Early safety indicators of COVID-19 convalescent plasma in 5,000 patients. J Clin Invest 2020;130:4791-7. [doi: 10.1172/JCI140200]

34. Joyner MJ, Bruno KA, Klassen SA, Kunze KL, Johnson PW, Lesser ER, et al. Safety update: COVID-19 convalescent plasma in 20,000 hospitalized patients. Mayo Clinic Proceedings 2020;95:1888-97. [doi: https://doi.org/10.1016/j.mayocp.2020.06.028]

35. Joyner MJ, Senefeld JW, Klassen SA, Mills JR, Johnson PW, Theel ES, et al. Effect of convalescent plasma on mortality among hospitalized patients with COVID-19: Initial three-month experience. medRxiv 2020. [doi: 10.1101/2020.08.12.20169359]

36. Liu M, Chen Z, Dai MY, Yang JH, Chen XB, Chen D, et al. Lessons learned from early compassionate use of convalescent plasma on critically ill patients with Covid-19. Transfusion 2020;in press. [doi: 10.1111/trf.15975]

37. Maor Y, Cohen D, Paran N, Israely T, Ezra V, Axelrod O, et al. Compassionate use of convalescent plasma for treatment of moderate and severe pneumonia in COVID-19 patients and association with IgG antibody levels in donated plasma. EClinicalMedicine 2020:100525. [doi: 10.1016/j.eclinm.2020.100525]

38. Naeem S, Gohh R, Bayliss G, Cosgrove C, Farmakiotis D, Merhi B, et al. Successful recovery from COVID-19 in three kidney transplant recipients who received convalescent plasma therapy. Transpl Infect Dis 2020:e13451. [doi: 10.1111/tid.13451]

39. Olivares-Gazca JC, Priesca-Marin JM, Ojeda-Laguna M, Garces-Eisele J, Soto-Olvera S, Palacios-Alonso A, et al. Infusion of convalescent plasma Is associated with clinical improvement in critically ill patients with Covid-19: A pilot study. Rev Invest Clin 2020;72:159-64. [doi: 10.24875/RIC.20000237]

40. Pal P, Ibrahim M, Niu A, Zwezdaryk KJ, Tatje E, Robinson IV WR, et al. Safety and efficacy of COVID-19 convalescent plasma in severe pulmonary disease: A report of 17 patients. Transfusion Medicine 2020;n/a. [doi: 10.1111/tme.12729]

41. Rahman F, Liu STH, Taimur S, Jacobs S, Sullivan T, Dunn D, et al. Treatment with convalescent plasma in solid organ transplant recipients with COVID-19: Experience at large transplant center in New York City. Clin Transplant 2020:e14089. [doi: 10.1111/ctr.14089]

42. Salazar E, Perez KK, Ashraf M, Chen J, Castillo B, Christensen PA, et al. Treatment of COVID-19 patients with convalescent plasma. Am J Pathol 2020. [doi: 10.1016/j.ajpath.2020.05.014]

43. Shen CG, Wang ZQ, Zhao F, Yang Y, Li JX, Yuan J, et al. Treatment of 5 critically Ill patients with COVID-19 with convalescent plasma. JAMA 2020;323:1582-9. [doi: 10.1001/jama.2020.4783]

44. Tremblay D, Seah C, Schneider T, Bhalla S, Feld J, Naymagon L, et al. Convalescent plasma for the treatment of severe COVID-19 infection in cancer patients. Cancer Med 2020. [doi: 10.1002/cam4.3457]

45. Wei B, Hang X, Xie Y, Zhang Y, Wang J, Cao X, et al. Long-term positive severe acute respiratory syndrome coronavirus 2 ribonucleic acid and therapeutic effect of antivirals in patients with coronavirus disease: Case reports. Rev Soc Bras Med Trop 2020;53:e20200372. [doi: 10.1590/0037-8682-0372-2020]

46. Wang M, Yang X, Yang F, Zhu X, Sun Z, Bao P, et al. Convalescent plasma therapy in critically ill coronavirus disease 2019 patients with persistently positive nucleic acid test, case series report. Medicine 2020;99:e21596. [doi: 10.1097/md.0000000000021596]

47. Wu Y, Hong K, Ruan L, Yang X, Zhang J, Xu J, et al. Patients with prolonged positivity of SARS-CoV-2 RNA benefit from convalescent plasma therapy: A retrospective study. Virol Sin 2020. [doi: 10.1007/s12250-020-00281-8]

48. Xi A, Zhuo M, Dai J, Ding Y, Ma X, Wang X, et al. Epidemiological and clinical characteristics of discharged patients infected with SARS-CoV-2 on the Qinghai Plateau. J Med Virol 2020;92:2528-35. [doi: 10.1002/jmv.26032]

49. Ye M, Fu D, Ren Y, Wang F, Wang D, Zhang F, et al. Treatment with convalescent plasma for COVID-19 patients in Wuhan, China. J Med Virol 2020. [doi: 10.1002/jmv.25882]

50. Zhang B, Liu S, Tan T, Huang W, Dong Y, Chen L, et al. Treatment with convalescent plasma for critically Ill patients with severe acute respiratory syndrome coronavirus 2 infection. Chest 2020. [doi: 10.1016/j.chest.2020.03.039]

51. Zeng H, Wang D, Nie J, Liang H, Gu J, Zhao A, et al. The efficacy assessment of convalescent plasma therapy for COVID-19 patients: a multi-center case series. Signal Transduct Target Ther 2020;5:219. [doi: 10.1038/s41392-020-00329-x]

52. Abolghasemi H, Eshghi P, Cheraghali AM, Imani Fooladi AA, Bolouki Moghaddam F, Imanizadeh S, et al. Clinical efficacy of convalescent plasma for treatment of COVID-19 infections: Results of a multicenter clinical study. Transfusion and Apheresis Science 2020;59:102875. [doi: https://doi.org/10.1016/j.transci.2020.102875]

53. Duan K, Liu B, Li C, Zhang H, Yu T, Qu J, et al. Effectiveness of convalescent plasma therapy in severe COVID-19 patients. Proc Natl Acad Sci U S A 2020;117:9490-6. [doi: 10.1073/pnas.2004168117]

54. Hegerova L, Gooley TA, Sweerus KA, Maree C, Bailey N, Bailey M, et al. Use of convalescent plasma in hospitalized patients with COVID-19: case series. Blood 2020;136:759-62. [doi: 10.1182/blood.2020006964]

55. Liu STH, Lin HM, Baine I, Wajnberg A, Gumprecht JP, Rahman F, et al. Convalescent plasma treatment of severe COVID-19: a propensity score-matched control study. Nat Med 2020:2020.05.20.20102236. [doi: 10.1038/s41591-020-1088-9]

56. Perotti C, Baldanti F, Bruno R, Del Fante C, Seminari E, Casari S, et al. Mortality reduction in 46 severe Covid-19 patients treated with hyperimmune plasma. A proof of concept single arm multicenter trial. Haematologica 2020;105:2834-40. [doi: 10.3324/haematol.2020.261784]

57. Rasheed AM, Fatak DF, Hashim HA, Maulood MF, Kabah KK, Almusawi YA, et al. The therapeutic potential of convalescent plasma therapy on treating critically-ill COVID-19 patients residing in respiratory care units in hospitals in Baghdad, Iraq. Infez Med 2020;28:357-66. [doi:

58. Rogers R, Shehadeh F, Mylona E, Rich J, Neill M, Touzard-Romo F, et al. Convalescent plasma for patients with severe COVID-19: a matched cohort study. medRxiv 2020:2020.08.18.20177402. [doi: 10.1101/2020.08.18.20177402]

59. Salazar E, Christensen PA, Graviss EA, Nguyen DT, Castillo B, Chen J, et al. Treatment of COVID-19 patients with convalescent plasma reveals a signal of significantly decreased mortality. Am J Pathol 2020;190:2290-303. [doi: https://doi.org/10.1016/j.ajpath.2020.08.001]

60. Xia X, Li K, Wu L, Wang Z, Zhu M, Huang B, et al. Improved clinical symptoms and mortality on severe/critical COVID-19 patients utilizing convalescent plasma transfusion. Blood 2020;136:755-9. [doi: 10.1182/blood.2020007079]

61. Xiao K, Lin Y, Fan Z, Wen Y, Huang H, Wang M, et al. Effect of transfusion convalescent recovery plasma in patients with coronavirus disease 2019. Zhong Nan Da Xue Xue Bao Yi Xue Ban 2020;45:565-70. [doi: 10.11817/j.issn.1672-7347.2020.200318]

62. Zeng QL, Yu ZJ, Gou JJ, Li GM, Ma SH, Zhang GF, et al. Effect of convalescent plasma therapy on viral shedding and survival in patients with coronavirus disease 2019. J Infect Dis 2020;222:38-43. [doi: 10.1093/infdis/jiaa228]

63. Gharbharan A, Jordans CCE, GeurtsvanKessel C, den Hollander JG, Karim F, Mollema FPN, et al. Convalescent plasma for COVID-19. A randomized clinical trial. medRxiv 2020:2020.07.01.20139857. [doi: 10.1101/2020.07.01.20139857]

64. Li L, Zhang W, Hu Y, Tong X, Zheng S, Yang J, et al. Effect of convalescent plasma therapy on time to clinical improvement in patients with severe and life-threatening COVID-19: A randomized clinical trial. JAMA 2020;324:460-70. [doi: 10.1001/jama.2020.10044]

65. Alghamdi AN and Abdel-Moneim AS. Convalescent plasma: A potential life-saving therapy for coronavirus disease 2019 (COVID-19). Front Public Health 2020;8:437. [doi: 10.3389/fpubh.2020.00437]

66. Alzoughool F and Alanagreh L. Coronavirus drugs: Using plasma from recovered patients as a treatment for COVID-19. Int J Risk Saf Med 2020;31:47-51. [doi: 10.3233/JRS-201017]

67. Borlongan MC and Sanberg PR. The disillusioned comfort with COVID-19 and the potential of convalescent plasma and cell therapy. Cell Transplant 2020;29:963689720940719. [doi: 10.1177/0963689720940719]

68. Cantore I and Valente P. Convalescent plasma from COVID 19 patients enhances intensive care unit survival rate. A preliminary report. Transfus Apher Sci 2020:102848. [doi: 10.1016/j.transci.2020.102848]

69. Casadevall A, Joyner MJ, Pirofski LA. A randomized trial of convalescent plasma for COVID-19-potentially hopeful signals. JAMA 2020;324:455-7. [doi: 10.1001/jama.2020.10218]

70. Casadevall A and Pirofski LA. The convalescent sera option for containing COVID-19. J Clin Invest 2020;130:1545-8. [doi: 10.1172/JCI138003]

71. Casadevall A and Tobian AAR. Convalescent plasma for COVID-19 - encouraging signals of efficacy. Br J Haematol 2021;192:681-2. [doi: 10.1111/bjh.17270]

72. Chen L, Xiong J, Bao L, Shi Y. Convalescent plasma as a potential therapy for COVID-19. Lancet Infect Dis 2020;20:398-400. [doi: 10.1016/S1473-3099(20)30141-9]

73. Cheraghali AM, Abolghasemi H, Eshghi P. Management of COVID-19 virus infection by convalescent plasma. Iran J Allergy Asthma Immunol 2020;19:3-6. [doi: 10.18502/ijaai.v19i(s1.r1).2847]

74. Gazzaruso C, Valenti C, Coppola A, Gallotti P. Impact of convalescent and nonimmune plasma on mortality of patients with COVID-19. A potential role for antithrombin. Clin Microbiol Infect 2020. [doi: 10.1016/j.cmi.2020.09.007]

75. Farhat RM, Mousa MA, Daas EJ, Glassberg MK. Treatment of COVID-19: Perspective on convalescent plasma transfusion. Front Med (Lausanne) 2020;7:435. [doi: 10.3389/fmed.2020.00435]

76. Focosi D, Tuccori M, Antonelli G, Maggi F. What is the optimal usage of coronavirus disease 2019 convalescent plasma donations? Clin Microbiol Infect 2020;27:S1198-743X(20)30589-9. [doi: 10.1016/j.cmi.2020.09.036]

77. Franchini M. Why should we use convalescent plasma for COVID-19? Eur J Intern Med 2020. [doi: 10.1016/j.ejim.2020.05.019]

78. Franchini M, Del Fante C, Klersy C, Glingani C, Percivalle E, Baldanti F, et al. Challenges in the production of convalescent hyperimmune plasma in the age of COVID-19. Semin Thromb Hemost 2020. [doi: 10.1055/s-0040-1713433]

79. Franchini M, Marano G, Velati C, Pati I, Pupella S, Liumbruno GM. Vox Sanguinis international forum on hospital transfusion services' response to COVID-19. Vox Sang 2020. [doi: 10.1111/vox.12967]

80. Franchini M, Marano G, Velati C, Pati I, Pupella S, Maria Liumbruno G. Operational protocol for donation of anti-COVID-19 convalescent plasma in Italy. Vox Sang 2020. [doi: 10.1111/vox.12940]

81. Islam A, Rafiq S, Karim S, Laher I, Rashid H. Convalescent plasma therapy in the treatment of COVID-19: Practical considerations: Correspondence. Int J Surg 2020;79:204-5. [doi: 10.1016/j.ijsu.2020.05.079]

82. Kesici S, Yavuz S, Bayrakci B. Get rid of the bad first: Therapeutic plasma exchange with convalescent plasma for severe COVID-19. Proc Natl Acad Sci U S A 2020;117:12526-7. [doi: 10.1073/pnas.2006691117]

83. Knudson CM and Jackson JB. COVID-19 convalescent plasma: phase 2. Transfusion 2020;60:1332-3. [doi: 10.1111/trf.15842]

84. Kumar S, Sharma V, Priya K. Battle against COVID-19: Efficacy of convalescent plasma as an emergency therapy. Am J Emerg Med 2020. [doi: 10.1016/j.ajem.2020.05.101]

85. McAllister F, Mantegazza A, Garzon F, Rotbaum V, Remondino G, Vazquez Larsson M, et al. Use of convalescent plasma for COVID-19 treatment. History and evidence. Medicina (B Aires) 2020;80 Suppl 3:82-6. [doi:

86. Montelongo-Jauregui D, Vila T, Sultan AS, Jabra-Rizk MA. Convalescent serum therapy for COVID-19: A 19th century remedy for a 21st century disease. 2020;16:e1008735. [doi: 10.1371/journal.ppat.1008735]

87. Morabito CJ and Gangadharan B. Active Therapy with Passive Immunotherapy May Be Effective in the Fight against COVID-19. Clin Transl Sci 2020;13:835-7. [doi: 10.1111/cts.12816]

88. Pau AK, Aberg J, Baker J, Belperio PS, Coopersmith C, Crew P, et al. Convalescent plasma for the treatment of COVID-19: Perspectives of the National Institutes of Health COVID-19 treatment guidelines panel. Ann Intern Med 2020;0:null. [doi: 10.7326/m20-6448 %m 32976026]

89. Perez-Cameo C and Marin-Lahoz J. Serosurveys and convalescent plasma in COVID-19. EClinicalMedicine 2020;23:100370. [doi: 10.1016/j.eclinm.2020.100370]

90. Rabelo-da-Ponte FD, Silvello D, Scherer JN, Ayala AR, Klamt F. Convalescent plasma therapy on patients with severe or life-threatening COVID-19: A metadata analysis. J Infect Dis 2020;222:1575-8. [doi: 10.1093/infdis/jiaa509]

91. Roback JD and Guarner J. Convalescent plasma to treat COVID-19: Possibilities and challenges. JAMA 2020;323:1561-2. [doi: 10.1001/jama.2020.4940]

92. Roberts DJ, Miflin G, Estcourt L. Convalescent plasma for COVID-19: Back to the future. Transfus Med 2020;30:174-6. [doi: 10.1111/tme.12700]

93. Rubin R. Testing an old therapy against a new disease: Convalescent plasma for COVID-19. JAMA 2020. [doi: 10.1001/jama.2020.7456]

94. Sabando Velez BE, Plaza Meneses C, Felix M, Vanegas E, Mata VL, Romero Castillo H, et al. A practical approach for the compassionate use of convalescent plasma in patients with severe COVID-19 in developing countries. J Infect Dev Ctries 2020;14:737-41. [doi: 10.3855/jidc.12827]

95. Sahu KK, Jindal V, Siddiqui AD, Cerny J, Gerber JM. Convalescent plasma therapy: A passive therapy for an aggressive COVID-19. J Med Virol 2020. [doi: 10.1002/jmv.26047]

96. Sheikh S and Baig MA. Convalescent Plasma: Promise for COVID-19 Pandemic. J Coll Physicians Surg Pak 2020;30:88. [doi: 10.29271/jcpsp.2020.Supp1.S88]

97. Sheridan C. Convalescent serum lines up as first-choice treatment for coronavirus. Nature biotechnology 2020. [doi:

98. Syal K. COVID-19: Herd immunity and convalescent plasma transfer therapy. J Med Virol 2020. [doi: 10.1002/jmv.25870]

99. Teixeira da Silva JA. Convalescent plasma: A possible treatment of COVID-19 in India. Med J Armed Forces India 2020. [doi: 10.1016/j.mjafi.2020.04.006]

100. The Lancet H. The resurgence of convalescent plasma therapy. The Lancet. Haematology 2020;7:e353-e. [doi: 10.1016/s2352-3026(20)30117-4]

101. Tonn T, Corman VM, Johnsen M, Richter A, Rodionov RN, Drosten C, et al. Stability and neutralising capacity of SARS-CoV-2-specific antibodies in convalescent plasma. Lancet Microbe 2020;1:e63. [doi: 10.1016/S2666-5247(20)30037-9]

102. Wong HK and Lee CK. Pivotal role of convalescent plasma in managing emerging infectious diseases. Vox Sang 2020;n/a. [doi: 10.1111/vox.12927]

103. Yoo JH. Convalescent plasma therapy for corona rirus disease 2019: a long way to go but worth trying. J Korean Med Sci 2020;35:e150-e. [doi: 10.3346/jkms.2020.35.e150]

104. Zhao Q and He Y. Challenges of convalescent plasma therapy on COVID-19. J Clin Virol 2020;127:104358. [doi: 10.1016/j.jcv.2020.104358]

105. Zhu M, Hu K, Zhu Z. Use of convalescent plasma in COVID-19 patients in China. Transfus Clin Biol 2020. [doi: 10.1016/j.tracli.2020.05.001]

106. Adriana T and Marco M. Immunoglobulins or convalescent plasma to tackle COVID-19: buying time to save lives current situation and perspectives. Swiss Med Wkly 2020;150. [doi: ARTN w2026410.4414/smw.2020.20264]

107. Begum F and Ray U. Polymonoclonal (not polyclonal) antibodies derived from convalescent human B cell hybridomas might be a better therapeutic option than single target monoclonal antibodies. ACS Pharmacol Transl Sci 2020;3:786-7. [doi: 10.1021/acsptsci.0c00084]

108. Bloch EM. Convalescent plasma to treat COVID-19. Blood 2020;136:654-5. [doi: 10.1182/blood.2020007714]

109. Brown B. Response Letter: Treatment for emerging viruses: Convalescent plasma and COVID-19. Transfus Apher Sci 2020:102929. [doi: 10.1016/j.transci.2020.102929]

110. Casadevall A, Joyner MJ, Pirofski L-a. Implications of coronavirus disease 2019 (COVID-19) antibody dynamics for immunity and convalescent plasma therapy. Clinical Infectious Diseases 2020. [doi: 10.1093/cid/ciaa1213]

111. Casadevall A, Joyner MJ, Pirofski LA. SARS-CoV-2 viral load and antibody responses: the case for convalescent plasma therapy. J Clin Invest 2020;130:5112-4. [doi: 10.1172/JCI139760]

112. Cunningham AC, Goh HP, Koh D. Treatment of COVID-19: old tricks for new challenges. Critical Care 2020;24. [doi: ARTN 9110.1186/s13054-020-2818-6]

113. Dhanasekaran S, Vajravelu LK, Venkatesalu V. Risk-benefit analysis on the clinical significance of convalescent plasma therapy in the management of COVID-19. Postgrad Med J 2020. [doi: 10.1136/postgradmedj-2020-138056]

114. Dzik S. COVID-19 Convalescent Plasma: Now Is the Time for Better Science. Transfus Med Rev 2020. [doi: 10.1016/j.tmrv.2020.04.002]

115. Estcourt LJ and Roberts DJ. Convalescent plasma for covid-19. BMJ 2020;370:m3516. [doi: 10.1136/bmj.m3516]

116. Farrugia A. Plasma from donors convalescent from SARS-CoV-2 infection-A matter of priorities. Transfus Clin Biol 2020;27:167-8. [doi: 10.1016/j.tracli.2020.05.002]

117. Fleming AB and Raabe V. Current studies of convalescent plasma therapy for COVID-19 may underestimate risk of antibody-dependent enhancement. J Clin Virol 2020;127:104388. [doi: 10.1016/j.jcv.2020.104388]

118. Focosi D. Anti-A Isohemagglutinin titers and SARS-CoV2 neutralization: implications for children and convalescent plasma selection. Br J Haematol 2020. [doi: 10.1111/bjh.16932]

119. Garraud O. Passive immunotherapy with convalescent plasma against COVID-19? What about the evidence base and clinical trials? Transfusion and Apheresis Science 2020;59:102858. [doi: https://doi.org/10.1016/j.transci.2020.102858]

120. Gniadek TJ and Donnersberger D. COVID-19 convalescent plasma donor recruitment: beware the Faustian bargains. Transfusion 2020. [doi: 10.1111/trf.15871]

121. Han G and Zhou YH. Thinking more about therapy with convalescent plasma for COVID-19 patients. Hum Vaccin Immunother 2020. [doi: 10.1080/21645515.2020.1787073]

122. Katz LM. (A Little) Clarity on convalescent plasma for Covid-19. New England Journal of Medicine 2021;384:666-8. [doi: 10.1056/NEJMe2035678]

123. Langhi DMJ, Santis GC, Bordin JO. COVID-19 convalescent plasma transfusion. Hematol Transfus Cell Ther 2020;42:113-5. [doi: 10.1016/j.htct.2020.04.003]

124. Lanza F and Seghatchian J. Reflection on passive immunotherapy in those who need most: some novel strategic arguments for obtaining safer therapeutic plasma or autologous antibodies from recovered COVID-19 infected patients. Br J Haematol 2020;190:e27-e9. [doi: 10.1111/bjh.16814]

125. Mahase E. Covid-19: US approves emergency use of convalescent plasma despite warnings over lack of evidence. BMJ 2020;370:m3327. [doi: 10.1136/bmj.m3327]

126. Mahase E. Covid-19: US FDA fires spokesperson over misleading claims about convalescent plasma. BMJ 2020;370:m3400. [doi: 10.1136/bmj.m3400]

127. Malani AN, Sherbeck JP, Malani PN. Convalescent plasma and COVID-19. JAMA 2020. [doi: 10.1001/jama.2020.10699]

128. Pawitan AJ. Convalescent plasma for COVID-19 considerations. Transfus Apher Sci 2020:102927. [doi: 10.1016/j.transci.2020.102927]

129. Prajapati S. Isopathic remedy prepared from convalescent plasma as a therapeutic option for COVID-19? Homeopathy 2020;109:184-5. [doi: 10.1055/s-0040-1714061]

130. Saverino D. Hyper-immune/convalescent plasma: an old option and a valid strategy for treatment of COVID-19? Minerva Med 2020. [doi: 10.23736/S0026-4806.20.06616-1]

131. Stevens MP, Patel PK, Nori P. Antimicrobial Stewardship Programs and convalescent plasma for COVID-19: A new paradigm for pre-authorization? Infect Control Hosp Epidemiol 2020:1-4. [doi: 10.1017/ice.2020.459]

132. Tedder RS and Semple MG. Appropriate selection of convalescent plasma donors for COVID-19. Lancet Infect Dis 2020:S1473-3099(20)30470-9. [doi: 10.1016/s1473-3099(20)30470-9]

133. Van den Berg K, Vermeulen M, Glatt TN, Wasserman S, Barrett CL, Peter J, et al. COVID-19: Convalescent plasma as a potential therapy. S Afr Med J 2020;110:562-3. [doi:

134. Verkerke HP and Maier CL. Towards characterized convalescent plasma for COVID-19: The dose matters. EClinicalMedicine 2020;26. [doi: 10.1016/j.eclinm.2020.100545]

135. Xi Y. Convalescent plasma therapy for COVID-19: a tried-and-true old strategy? Signal Transduct Target Ther 2020;5:203. [doi: 10.1038/s41392-020-00310-8]

136. Zeng F, Chen X, Deng G. Convalescent plasma for patients with COVID-19. Proc Natl Acad Sci U S A 2020;117:12528. [doi: 10.1073/pnas.2006961117]

137. Zylberman V, Sanguineti S, Pontoriero AV, Higa SV, Cerutti ML, Morrone Seijo SM, et al. Development of a hyperimmune equine serum therapy for COVID-19 in Argentina. Medicina (B Aires) 2020;80 Suppl 3:1-6. [doi:

138. Caccamo N, Sullivan LC, Brooks AG, Dieli F. Harnessing HLA-E-restricted CD8 T lymphocytes for adoptive cell therapy of severe COVID-19 patients. Br J Haematol 2020. [doi: 10.1111/bjh.16895]

139. Ferreira LMR and Mostajo-Radji MA. Plasma-based COVID-19 treatments in low- and middle-income nations pose a high risk of an HIV epidemic. NPJ Vaccines 2020;5:58. [doi: 10.1038/s41541-020-0209-2]

140. Joob B and Wiwanitkit V. Convalescent plasma and covid-19 treatment. Minerva Med 2020. [doi: 10.23736/S0026-4806.20.06670-7]

141. Sanfilippo F, La Rosa V, Oliveri F, Astuto M. Convalescent plasma for COVID-19: the risk of pulmonary embolism should not be underestimated! Critical Care 2020;24:531. [doi: 10.1186/s13054-020-03236-3]

142. Sanfilippo F, La Rosa V, Oliveri F, Astuto M. COVID-19, hypercoagulability and cautiousness with convalescent plasma. Am J Respir Crit Care Med 2020. [doi: 10.1164/rccm.202008-3139LE]

143. Wiwanitkit V. Convalescent plasma therapy in the treatment of COVID-19: Some considerations: Correspondence. Int J Surg 2020;80:26. [doi: 10.1016/j.ijsu.2020.06.029]

144. Barone P and DeSimone RA. Convalescent plasma to treat coronavirus disease 2019 (COVID-19): considerations for clinical trial design. Transfusion 2020;60:1123-7. [doi: 10.1111/trf.15843]

145. Majbour N and El-Agnaf O. Plasma-derived therapy: can the survivors of COVID-19 help the defenseless? Diagnosis (Berl) 2020;7:373-6. [doi: 10.1515/dx-2020-0053]

146. Brown BL and McCullough J. Treatment for emerging viruses: Convalescent plasma and COVID-19. Transfus Apher Sci 2020;59:102790. [doi: 10.1016/j.transci.2020.102790]

147. Focosi D, Tang J, Anderson A, Tuccori M. Convalescent plasma therapy for COVID-19: State of the Art. Preprints 2020. [doi:

148. Cao HL and Shi Y. Convalescent plasma: possible therapy for novel coronavirus disease 2019. Transfusion 2020;60:1078-83. [doi: 10.1111/trf.15797]

149. Zheng K, Liao G, Lalu MM, Tinmouth A, Fergusson DA, Allan DS. A scoping review of registered clinical trials of convalescent plasma for COVID-19 and a framework for accelerated synthesis of trial evidence (FAST Evidence). Transfus Med Rev 2020;34:158-64. [doi: 10.1016/j.tmrv.2020.06.005]

150. de Alwis R, Chen S, Gan ES, Ooi EE. Impact of immune enhancement on Covid-19 polyclonal hyperimmune globulin therapy and vaccine development. EBioMedicine 2020;55:102768. [doi: 10.1016/j.ebiom.2020.102768]

151. Fischer JC, Zanker K, van Griensven M, Schneider M, Kindgen-Milles D, Knoefel WT, et al. The role of passive immunization in the age of SARS-CoV-2: an update. Eur J Med Res 2020;25:16. [doi: 10.1186/s40001-020-00414-5]

152. Mucha SR and Quraishy N. Convalescent plasma for COVID-19. Cleve Clin J Med 2020. [doi: 10.3949/ccjm.87a.ccc056]

153. Piechotta V, Chai KL, Valk SJ, Doree C, Monsef I, Wood EM, et al. Convalescent plasma or hyperimmune immunoglobulin for people with COVID‐19: a living systematic review. Cochrane Database of Systematic Reviews 2020;7:CD013600. [doi: 10.1002/14651858.CD013600.pub2]

154. Chai KL, Valk SJ, Piechotta V, Kimber C, Monsef I, Doree C, et al. Convalescent plasma or hyperimmune immunoglobulin for people with COVID‐19: a living systematic review. Cochrane Database of Systematic Reviews 2020;10:CD013600. [doi: 10.1002/14651858.CD013600.pub3]

155. Devasenapathy N, Ye Z, Loeb M, Fang F, Najafabadi BT, Xiao Y, et al. Efficacy and safety of convalescent plasma for severe COVID-19 based on evidence in other severe respiratory viral infections: a systematic review and meta-analysis. CMAJ 2020. [doi: 10.1503/cmaj.200642]

156. Sarkar S, Soni KD, Khanna P. Convalescent plasma is a clutch at straws in COVID-19 management! A systematic review and meta-analysis. J Med Virol 2020;n/a. [doi: 10.1002/jmv.26408]

157. Sun M, Xu Y, He H, Zhang L, Wang X, Qiu Q, et al. A potentially effective treatment for COVID-19: A systematic review and meta-analysis of convalescent plasma therapy in treating severe infectious disease. International Journal of Infectious Diseases 2020;98:334-46. [doi: 10.1016/j.ijid.2020.06.107]

158. Abdollahi H, Shiri I, Bevelacqua JJ, Jafarzadeh A, Rahmim A, Zaidi H, et al. Low dose radiation therapy and convalescent plasma: How a hybrid method may maximize benefits for COVID-19 patients. Journal of Biomedical Physics and Engineering 2020;10:387-94. [doi: 10.31661/jbpe.v0i0.2006-1125]

159. Annamaria P, Eugenia Q, Paolo S. Anti-SARS-CoV-2 hyperimmune plasma workflow. Transfus Apher Sci 2020:102850. [doi: 10.1016/j.transci.2020.102850]

160. Anudeep T, Jeyaraman M, Shetty DU, Raj H, Ajay S, Rajeswari S, et al. Convalescent Plasma as a plausible therapeutic option in nCOVID-19–A Review. J Clin Trials 2020;10:No:1000409. [doi:

161. Bloch EM, Shoham S, Casadevall A, Sachais BS, Shaz B, Winters JL, et al. Deployment of convalescent plasma for the prevention and treatment of COVID-19. J Clin Invest 2020;130:2757-65. [doi: 10.1172/JCI138745]

162. Kumar GV, Jeyanthi V, Ramakrishnan S. A short review on antibody therapy for COVID-19. New Microbes New Infect 2020:100682. [doi: 10.1016/j.nmni.2020.100682]

163. Gasparyan AY, Misra DP, Yessirkepov M, Zimba O. Perspectives of immune therapy in coronavirus disease 2019. J Korean Med Sci 2020;35:e176. [doi: 10.3346/jkms.2020.35.e176]

164. Iftikhar A, Jabeen F, Manzoor M, Younis T, Shaheen M. Passive immunization: Paradoxical and traditional method for new pandemic challenge COVID-19. Acta Microbiol Immunol Hung 2020;67:87-90. [doi: 10.1556/030.2020.01199]

165. Li S, Zhao H, Sun Y, Wang P, Li H, Duan M. Application of convalescent plasma for the treatment of adult patients with coronavirus disease 2019. Zhonghua Wei Zhong Bing Ji Jiu Yi Xue 2020;32:646-51. [doi: 10.3760/cma.j.cn121430-20200601-00479]

166. Lindholm PF, Ramsey G, Kwaan HC. Passive immunity for coronavirus disease 2019: A commentary on therapeutic aspects including convalescent plasma. Semin Thromb Hemost 2020. [doi: 10.1055/s-0040-1712157]

167. Murphy M, Estcourt L, Grant-Casey J, Dzik S. International Survey of Trials of Convalescent Plasma to Treat COVID-19 Infection. Transfus Med Rev 2020;34:151-7. [doi: https://doi.org/10.1016/j.tmrv.2020.06.003]

168. Sayinalp B, Çinar OE, HaznedaroĞlu İ C. Perspectives for the immune plasma treatment of COVID-19. Turk J Med Sci 2020. [doi: 10.3906/sag-2005-410]

169. Subbarao K, Mordant F, Rudraraju R. Convalescent plasma treatment for COVID-19: tempering expectations with the influenza experience. Eur J Immunol 2020. [doi: 10.1002/eji.202048723]

170. Pawar AY, Hiray AP, Sonawane DD, Bhambar RS, Derle DV, Ahire YS. Convalescent plasma: A possible treatment protocol for COVID- 19 patients suffering from diabetes or underlying liver diseases. Diabetes Metab Syndr 2020;14:665-9. [doi: 10.1016/j.dsx.2020.05.023]

171. Bakhtawar N, Usman M, Khan MMU. Convalescent plasma therapy and its effects on COVID-19 patient outcomes: A systematic review of current literature. Cureus 2020;12:e9535. [doi: 10.7759/cureus.9535]

172. Chen B and Xia R. Early experience with convalescent plasma as immunotherapy for COVID-19 in China: Knowns and unknowns. Vox Sang 2020. [doi: 10.1111/vox.12968]

173. Rajendran K, Krishnasamy N, Rangarajan J, Rathinam J, Natarajan M, Ramachandran A. Convalescent plasma transfusion for the treatment of COVID-19: Systematic review. J Med Virol 2020. [doi: 10.1002/jmv.25961]

174. Valk SJ, Piechotta V, Chai KL, Doree C, Monsef I, Wood EM, et al. Convalescent plasma or hyperimmune immunoglobulin for people with COVID-19: a rapid review. Cochrane Database Syst Rev 2020;5:CD013600. [doi: 10.1002/14651858.CD013600]

175. Wooding DJ and Bach H. Treatment of COVID-19 with convalescent plasma: lessons from past coronavirus outbreaks. Clin Microbiol Infect 2020. [doi: 10.1016/j.cmi.2020.08.005]

176. Daniele F and Albert F. The art of the possible in approaching efficacy trials for COVID19 convalescent plasma. Int J Infect Dis 2020;102:244-6. [doi: 10.1016/j.ijid.2020.10.074]

177. Nagoba B, Gavkare A, Jamadar N, Mumbre S, Selkar S. Positive aspects, negative aspects and limitations of plasma therapy with special reference to COVID-19. J Infect Public Health 2020. [doi: 10.1016/j.jiph.2020.08.011]

178. Psaltopoulou T, Sergentanis TN, Pappa V, Politou M, Terpos E, Tsiodras S, et al. The emerging role of convalescent plasma in the treatment of COVID-19. HemaSphere 2020;4:e409. [doi: 10.1097/hs9.0000000000000409]

179. Tiberghien P, de Lamballerie X, Morel P, Gallian P, Lacombe K, Yazdanpanah Y. Collecting and evaluating convalescent plasma for COVID-19 treatment: why and how? Vox Sang 2020. [doi: 10.1111/vox.12926]

180. Choi JY. Convalescent plasma therapy for coronavirus disease 2019. Infect Chemother 2020;52:307-16. [doi:

181. Khulood D, Adil MS, Sultana R, Nimra. Convalescent plasma appears efficacious and safe in COVID-19. Therapeutic Advances in Infectious Disease 2020:First Published September 28, 2020. [doi: 10.1177/2049936120957931]

182. Long CVK, Sayed A, Karki P, Acharya Y. Convalescent blood products in COVID-19: A narrative review. Therapeutic Advances in Infectious Disease 2020;7:Article first published online: September 22, 2020. [doi: 10.1177/2049936120960646]

183. Ouyang J, Isnard S, Lin J, Fombuena B, Peng X, Routy J-P, et al. Convalescent plasma: The relay baton in the race for coronavirus disease 2019 treatment. Front Immunol 2020;11. [doi: 10.3389/fimmu.2020.570063]

184. Piyush R, Rajarshi K, Khan R, Ray S. Convalescent plasma therapy: a promising coronavirus disease 2019 treatment strategy. Open Biol 2020;10:200174. [doi: 10.1098/rsob.200174]

185. Rojas M, Rodriguez Y, Monsalve DM, Acosta-Ampudia Y, Camacho B, Gallo JE, et al. Convalescent plasma in Covid-19: Possible mechanisms of action. Autoimmun Rev 2020;19:102554. [doi: 10.1016/j.autrev.2020.102554]

186. Selvi V. Convalescent plasma: A challenging tool to treat COVID-19 patients—A Lesson from the past and new perspectives. BioMed Research International 2020;2020:2606058. [doi: 10.1155/2020/2606058]

187. Sharun K, Tiwari R, Iqbal Yatoo M, Patel SK, Natesan S, Dhama J, et al. Antibody-based immunotherapeutics and use of convalescent plasma to counter COVID-19: advances and prospects. Expert Opin Biol Ther 2020;20:1033-46. [doi: 10.1080/14712598.2020.1796963]

188. Sullivan HC and Roback JD. Convalescent plasma: Therapeutic hope or hopeless strategy in the SARS-CoV-2 pandemic. Transfus Med Rev 2020. [doi: 10.1016/j.tmrv.2020.04.001]

189. Yigenoglu TN, Hacibekiroglu T, Berber I, Dal MS, Basturk A, Namdaroglu S, et al. Convalescent plasma therapy in patients with COVID-19. J Clin Apher 2020;35:367-73. [doi: 10.1002/jca.21806]

190. Accorsi P, Berti P, de Angelis V, De Silvestro G, Mascaretti L, Ostuni A. Position paper on the preparation of immune plasma to be used in the treatment of patients with COVID-19. Blood Transfus 2020;18:163-6. [doi: 10.2450/2020.0124-20]

191. Albalawi M, Zaidi SZA, AlShehry N, AlAskar A, Zaidi ARZ, Abdallah RNM, et al. Safety and efficacy of convalescent plasma to treat severe COVID-19: Protocol for the Saudi collaborative multi-center phase II study. JMIR Res Protoc 2020. [doi: 10.2196/23543]

192. Al-Riyami AZ, Schafer R, van der Berg K, Bloch EM, Escourt LJ, Goel R, et al. Clinical use of Convalescent Plasma in the COVID-19 pandemic; a transfusion-focussed gap analysis with recommendations for future research priorities. Vox Sang 2020. [doi: 10.1111/vox.12973]

193. Albahri OS, Al-Obaidi JR, Zaidan AA, Albahri AS, Zaidan BB, Salih MM, et al. Helping doctors hasten COVID-19 treatment: Towards a rescue framework for the transfusion of best convalescent plasma to the most critical patients based on biological requirements via ml and novel MCDM methods. Comput Methods Programs Biomed 2020;196:105617. [doi: 10.1016/j.cmpb.2020.105617]

194. Bloch EM, Goel R, Wendel S, Burnouf T, Al-Riyami AZ, Ang AL, et al. Guidance for the procurement of COVID-19 convalescent plasma: Differences between high and wow-middle income countries. Vox Sang 2020. [doi: 10.1111/vox.12970]

195. Blackall D, Wulff S, Roettger T, Jacobs L, Lacasse A, Patri M, et al. Rapid establishment of a COVID-19 convalescent plasma program in a regional health care delivery network. Transfusion 2020. [doi: 10.1111/trf.16026]

196. Budhai A, Wu AA, Hall L, Strauss D, Paradiso S, Alberigo J, et al. How did we rapidly implement a convalescent plasma program? Transfusion 2020;60:1348-55. [doi: 10.1111/trf.15910]

197. Chowdhury FR, Hoque A, Chowdhury FUH, Amin MR, Rahim A, Rahman MM, et al. Convalescent plasma transfusion therapy in severe COVID-19 patients- a safety, efficacy and dose response study: A structured summary of a study protocol of a phase II randomized controlled trial. Trials 2020;21:883. [doi: 10.1186/s13063-020-04734-z]

198. Eckhardt CM, Cummings MJ, Rajagopalan KN, Borden S, Bitan ZC, Wolf A, et al. Evaluating the efficacy and safety of human anti-SARS-CoV-2 convalescent plasma in severely ill adults with COVID-19: A structured summary of a study protocol for a randomized controlled trial. Trials 2020;21:499. [doi: 10.1186/s13063-020-04422-y]

199. Janssen M, Schäkel U, Djuka Fokou C, Krisam J, Stermann J, Kriegsmann K, et al. A randomized open label phase-II clinical trial with or without infusion of plasma from subjects after convalescence of SARS-CoV-2 infection in high-risk patients with confirmed severe SARS-CoV-2 disease (RECOVER): A structured summary of a study protocol for a randomised controlled trial. Trials 2020;21:828. [doi: 10.1186/s13063-020-04735-y]

200. Epstein J and Burnouf T. Points to consider in the preparation and transfusion of COVID-19 convalescent plasma. Vox Sang 2020. [doi: 10.1111/vox.12939]

201. Epstein J, Smid M, Wendel S, Somuah D, Burnouf T. Use of COVID-19 convalescent plasma in low- and middle-income countries: a call for ethical principles and the assurance of quality and safety. Vox Sang 2020. [doi: 10.1111/vox.12964]

202. Hassan MO, Osman AA, Elbasit HEA, Hassan HE, Rufai H, Satti MMM, et al. Convalescent plasma as a treatment modality for coronavirus disease 2019 in Sudan. Transfus Apher Sci 2020:102918. [doi: 10.1016/j.transci.2020.102918]

203. Ipe TS, Le T, Quinn B, Kellar S, Clark M, Carlisle S, et al. Provision of COVID-19 convalescent plasma in a resource-constrained state. Transfusion 2020;n/a. [doi: 10.1111/trf.16118]

204. Li L, Yang R, Wang J, Lv Q, Ren M, Zhao L, et al. Feasibility of a pilot program for COVID-19 convalescent plasma collection in Wuhan, China. Transfusion 2020. [doi: 10.1111/trf.15921]

205. Pei S, Yuan X, Zhang Z, Yao R, Xie Y, Shen M, et al. Convalescent plasma to treat COVID-19: Chinese strategy and experiences. medRxiv 2020:2020.04.07.20056440. [doi: 10.1101/2020.04.07.20056440]

206. Perotti C, Del Fante C, Baldanti F, Franchini M, Percivalle E, Vecchio Nepita E, et al. Plasma from donors recovered from the new Coronavirus 2019 as therapy for critical patients with COVID-19 (COVID-19 plasma study): a multicentre study protocol. Intern Emerg Med 2020;15:819-24. [doi: 10.1007/s11739-020-02384-2]

207. Seghatchian J and Lanza F. Convalescent plasma, an apheresis research project targeting and motivating the fully recovered COVID 19 patients: A rousing message of clinical benefit to both donors and recipients alike. Transfus Apher Sci 2020;59:102794. [doi: 10.1016/j.transci.2020.102794]

208. Yilmaz S, Ertugrul Oruc N, Ozcebe OI, Azap A, Cetin AT, Yenicesu I, et al. Regulatory consideration on preparation and clinical use of COVID-19 convalescent plasma. Transfus Apher Sci 2020:102846. [doi: 10.1016/j.transci.2020.102846]

209. Amanat F, Stadlbauer D, Strohmeier S, Nguyen THO, Chromikova V, McMahon M, et al. A serological assay to detect SARS-CoV-2 seroconversion in humans. Nat Med 2020;26:1033-6. [doi: 10.1038/s41591-020-0913-5]

210. Byrnes JR, Zhou XX, Lui I, Elledge SK, Glasgow JE, Lim SA, et al. A SARS-CoV-2 serological assay to determine the presence of blocking antibodies that compete for human ACE2 binding. medRxiv 2020. [doi: 10.1101/2020.05.27.20114652]

211. Gattinger P, Borochova K, Dorofeeva Y, Henning R, Kiss R, Kratzer B, et al. Antibodies in serum of convalescent patients following mild COVID-19 do not always prevent virus receptor binding. Allergy 2020. [doi: 10.1111/all.14523]

212. Hansen CB, Jarlhelt I, Pérez-Alós L, Hummelshøj Landsy L, Loftager M, Rosbjerg A, et al. SARS-CoV-2 antibody responses are correlated to disease severity in COVID-19 convalescent individuals. The Journal of Immunology 2021;206:109-17. [doi: 10.4049/jimmunol.2000898]

213. DomBourian MG, Annen K, Huey L, Andersen G, Merkel PA, Jung S, et al. Analysis of COVID-19 convalescent plasma for SARS-CoV-2 IgG using two commercial immunoassays. J Immunol Methods 2020:112837. [doi: 10.1016/j.jim.2020.112837]

214. Ding S, Laumaea A, Gasser R, Medjahed H, Pancera M, Stamatatos L, et al. Antibody binding to SARS-CoV-2 S glycoprotein correlates with, but does not predict neutralization. bioRxiv 2020. [doi: 10.1101/2020.09.08.287482]

215. Ianevski A, Yao R, Fenstad MH, Biza S, Zusinaite E, Reisberg T, et al. Potential antiviral options against SARS-CoV-2 infection. Viruses 2020;12. [doi: 10.3390/v12060642]

216. Schmidt F, Weisblum Y, Muecksch F, Hoffmann HH, Michailidis E, Lorenzi JCC, et al. Measuring SARS-CoV-2 neutralizing antibody activity using pseudotyped and chimeric viruses. J Exp Med 2020;217. [doi: 10.1084/jem.20201181]

217. Wang X, Guo X, Xin Q, Pan Y, Hu Y, Li J, et al. Neutralizing antibodies responses to SARS-CoV-2 in COVID-19 inpatients and convalescent patients. Clinical Infectious Diseases 2020;71:2688-94. [doi: 10.1093/cid/ciaa721]

218. Muruato AE, Fontes-Garfias CR, Ren P, Garcia-Blanco MA, Menachery VD, Xie X, et al. A high-throughput neutralizing antibody assay for COVID-19 diagnosis and vaccine evaluation. bioRxiv 2020. [doi: 10.1101/2020.05.21.109546]

219. Ragnesola B, Jin D, Lamb CC, Shaz BH, Hillyer CD, Luchsinger LL. COVID19 antibody detection using lateral flow assay tests in a cohort of convalescent plasma donors. BMC Res Notes 2020;13:372. [doi: 10.1186/s13104-020-05212-0]

220. Yang HS, Racine-Brzostek SE, Lee WT, Hunt D, Yee J, Chen Z, et al. SARS-CoV-2 antibody characterization in emergency department, hospitalized and convalescent patients by two semi-quantitative immunoassays. Clin Chim Acta 2020;509:117-25. [doi: 10.1016/j.cca.2020.06.004]

221. de Assis RR, Jain A, Nakajima R, Jasinskas A, Felgner J, Obiero JM, et al. Analysis of SARS-CoV-2 antibodies in COVID-19 convalescent plasma using a coronavirus antigen microarray. bioRxiv 2020. [doi: 10.1101/2020.04.15.043364]

222. Dulipsingh L, Ibrahim D, Schaefer EJ, Crowell R, Diffenderfer MR, Williams K, et al. SARS-CoV-2 serology and virology trends in donors and recipients of convalescent plasma. Transfus Apher Sci 2020:102922. [doi: 10.1016/j.transci.2020.102922]

223. Ikegami S, Benirschke R, Flanagan T, Tanna N, Klein T, Elue R, et al. Persistence of SARS-CoV-2 nasopharyngeal swab PCR positivity in COVID-19 convalescent plasma donors. Transfusion 2020. [doi: 10.1111/trf.16015]

224. Ma H, Zhao D, Zeng W, Yang Y, Hu X, Zhou P, et al. Decline of SARS-CoV-2-specific IgG, IgM and IgA in convalescent COVID-19 patients within 100 days after hospital discharge. Science China Life Sciences 2020. [doi: 10.1007/s11427-020-1805-0]

225. Danh K, Karp DG, Robinson PV, Seftel D, Stone M, Simmons G, et al. Detection of SARS-CoV-2 neutralizing antibodies with a cell-free PCR assay. medRxiv 2020. [doi: 10.1101/2020.05.28.20105692]

226. Hartman WR, Hess AS, Connor J. Persistent viral RNA shedding after COVID-19 symptom resolution in older convalescent plasma donors. Transfusion 2020. [doi: 10.1111/trf.15927]

227. Abe KT, Li Z, Samson R, Samavarchi-Tehrani P, Valcourt EJ, Wood H, et al. A simple protein-based surrogate neutralization assay for SARS-CoV-2. JCI Insight 2020;5. [doi: 10.1172/jci.insight.142362]

228. Beaudoin-Bussières G, Laumaea A, Anand SP, Prévost J, Gasser R, Goyette G, et al. Decline of humoral responses against SARS-CoV-2 spike in convalescent individuals. mBio 2020;11:e02590-20. [doi: 10.1128/mBio.02590-20]

229. Benner SE, Patel EU, Laeyendecker O, Pekosz A, Littlefield K, Eby Y, et al. SARS-CoV-2 antibody avidity responses in COVID-19 patients and convalescent plasma donors. J Infect Dis 2020;222:1974-84. [doi: 10.1093/infdis/jiaa581]

230. Boonyaratanakornkit J, Morishima C, Selke S, Zamora D, McGuffin S, Shapiro AE, et al. Clinical, laboratory, and temporal predictors of neutralizing antibodies to SARS-CoV-2 after COVID-19. medRxiv 2020:2020.10.06.20207472. [doi: 10.1101/2020.10.06.20207472]

231. Gniadek TJ, Thiede JM, Matchett WE, Gress AR, Pape KA, Fiege JK, et al. SARS-CoV-2 neutralization and serology testing of COVID-19 convalescent plasma from donors with non-severe disease. Transfusion 2020. [doi: 10.1111/trf.16101]

232. Patel E, Bloch EM, Clarke W, Hsieh YH, Boon D, Eby YJ, et al. Comparative performance of five commercially available serologic assays to detect antibodies to SARS-CoV-2 and identify individuals with high neutralizing titers. medRxiv 2020. [doi: 10.1101/2020.08.31.20184788]

233. Harvala H, Mehew J, Robb ML, Ijaz S, Dicks S, Patel M, et al. Convalescent plasma treatment for SARS-CoV-2 infection: analysis of the first 436 donors in England, 22 April to 12 May 2020. Euro Surveill 2020;25. [doi: 10.2807/1560-7917.es.2020.25.28.2001260]

234. Wendel S, Kutner JM, Machado R, Fontao-Wendel R, Bub C, Fachini R, et al. Screening for SARS-CoV-2 antibodies in convalescent plasma in Brazil: Preliminary lessons from a voluntary convalescent donor program. Transfusion 2020. [doi: 10.1111/trf.16065]

235. Zeng C, Evans JP, Pearson R, Qu P, Zheng YM, Robinson RT, et al. Neutralizing antibody against SARS-CoV-2 spike in COVID-19 patients, health care workers and convalescent plasma donors: a cohort study using a rapid and sensitive high-throughput neutralization assay. medRxiv 2020. [doi: 10.1101/2020.08.02.20166819]

236. Dogan M, Kozhaya L, Placek L, Gunter C, Yigit M, Hardy R, et al. Novel SARS-CoV-2 specific antibody and neutralization assays reveal wide range of humoral immune response during COVID-19. medRxiv 2020. [doi: 10.1101/2020.07.07.20148106]

237. Jungbauer C, Weseslindtner L, Weidner L, Gansdorfer S, Farcet MR, Gschaider-Reichhart E, et al. Characterization of 100 sequential SARS-CoV-2 convalescent plasma donations. Transfusion 2021;61:12-6. [doi: 10.1111/trf.16119]

238. Li L, Tong X, Chen H, He R, Lv Q, Yang R, et al. Characteristics and serological patterns of COVID-19 convalescent plasma donors: optimal donors and timing of donation. Transfusion 2020;60:1765-72. [doi: 10.1111/trf.15918]

239. Ni L, Ye F, Cheng ML, Feng Y, Deng YQ, Zhao H, et al. Detection of SARS-CoV-2-specific humoral and cellular immunity in COVID-19 convalescent individuals. Immunity 2020;52:971-7.e3. [doi: 10.1016/j.immuni.2020.04.023]

240. Robbiani DF, Gaebler C, Muecksch F, Lorenzi JCC, Wang Z, Cho A, et al. Convergent antibody responses to SARS-CoV-2 infection in convalescent individuals. bioRxiv 2020. [doi: 10.1101/2020.05.13.092619]

241. Salazar E, Kuchipudi SV, Christensen PA, Eagar TN, Yi X, Zhao P, et al. Relationship between anti-spike protein antibody titers and SARS-CoV-2 in vitro virus neutralization in convalescent plasma. bioRxiv 2020. [doi: 10.1101/2020.06.08.138990]

242. Weidner L, Gansdorfer S, Unterweger S, Weseslindtner L, Drexler C, Farcet M, et al. Quantification of SARS-CoV-2 antibodies with eight commercially available immunoassays. J Clin Virol 2020;129:104540. [doi: 10.1016/j.jcv.2020.104540]

243. Natarajan H, Crowley AR, Butler SE, Xu S, Weiner JA, Bloch EM, et al. SARS-CoV-2 antibody signatures robustly predict diverse antiviral functions relevant for convalescent plasma therapy. medRxiv 2020:2020.09.16.20196154. [doi: 10.1101/2020.09.16.20196154]
